# First evidence of an extensive Acheulean large cutting tool accumulation in Europe from Porto Maior (Galicia, Spain)

**DOI:** 10.1038/s41598-018-21320-1

**Published:** 2018-02-15

**Authors:** E. Méndez-Quintas, M. Santonja, A. Pérez-González, M. Duval, M. Demuro, L. J. Arnold

**Affiliations:** 1 Centro Nacional de Investigación sobre la Evolución Humana (CENIEH), Paseo de Atapuerca, 3, 09002 Burgos, Spain; 2 Escuela Interuniversitaria de Posgrado en Evolución Humana, Universidad de Burgos, Juan de Austria 1, 09001 Burgos, Spain; 3 Australian Research Centre for Human Evolution. Environmental Futures Research Institute, Griffith University, 170 Kessels Road, Nathan, QLD 4111 Australia; 4 School of Physical Sciences, Environment Institute, and Institute for Photonics and Advanced Sensing (IPAS), University of Adelaide, North Terrace Campus, Adelaide, SA 5005 Australia

## Abstract

We describe a European Acheulean site characterised by an extensive accumulation of large cutting tools (LCT). This type of Lower Paleolithic assemblage, with dense LCT accumulations, has only been found on the African continent and in the Near East until now. The identification of a site with large accumulations of LCTs favours the hypothesis of an African origin for the Acheulean of Southwest Europe. The lithic tool-bearing deposits date back to 293–205 thousand years ago. Our chronological findings confirm temporal overlap between sites with clear “African” Acheulean affinities and Early Middle Paleolithic sites found elsewhere in the region. These complex technological patterns could be consistent with the potential coexistence of different human species in south-western Europe during the Middle Pleistocene.

## Introduction

Understanding the chronology and origin of the European Acheulean is of fundamental importance for unravelling the Middle Pleistocene human occupation dynamics of the continent. Different explanations have been proposed for the emergence of the Acheulean stone tool tradition in Europe. The most widely supported explanation argues that the origin of the European Acheulean is linked to an “Out of Africa” scenario^1,2^, potentially one invoking a migration route through the Strait of Gibraltar^3–6^. An alternative explanation, albeit less popular, proposes local re-invention of the Acheulean, without a direct connection with the African technocomplex^7,8^ Most of the available dating evidence indicates that the Acheulean appeared in Europe from Marine Isotope Stage (MIS) 12 onwards and lasted until MIS 6 (534–130 ka)^3,4,9–11^, which is significantly shorter than the documented age range of the African Acheulean technocomplex (∼1.7–0.5 Ma)^5,12,13^. Scarce evidence of pre-MIS 12 Acheulean occupation sites in Europe have also been reported^14–18^, but some of them are not exempt from interpretative problems regarding their chronology or their technological identification. This is the case, for example, at La Solana del Zamborino, whose chronology has been recently revised to <500 thousand years ago (ka)^19^, as well as Cueva Negra and La Boella, which have both yielded too few diagnostic lithic elements to be unequivocally considered as Acheulean assemblages^3,20^. According to these data, any hypothetical re-invention of Acheulean technology in Europe would have occurred one million years after the emergence of the Acheulean in Africa in a different environmental and techno-cultural context, and most likely by a human species that was very different from the African inventor (*Homo ergaster*).

The broad-scale technological affinities between the Acheulean tradition of southwest (SW) Europe and the African Acheulean have been widely documented, especially those identified in the large Atlantic basins of the Iberian Peninsula and Aquitanian region (SW France)^2,4,21–23^. However, affinities between the types and functionality of Acheulean occupation sites on the two continents are less certain. Important differences also exist in the specific technological characteristics of the Acheulean tradition across Europe. In particular, there are notable differences in the occurrences of cleavers and the use of large flake blanks (LFB *sensu* Sharon^24^) for the configuration of large cutting tools (LCTs) between the Iberian Acheulean tradition and Acheulean industries found in northwest (NW) Europe^3–5,9,21,23^. Against this complex geographic backdrop and the ongoing debate surrounding the origins of the European Acheulean, we present important new archaeological evidence from the site of Porto Maior (Galicia, Spain). This represents the first European site to document an Acheulean occupation pattern comparable to that known exclusively from Africa, characterised by extensive accumulations of LCTs.

## Results

### Geomorphologic and morpho-stratigraphic setting

**Figure 1:**
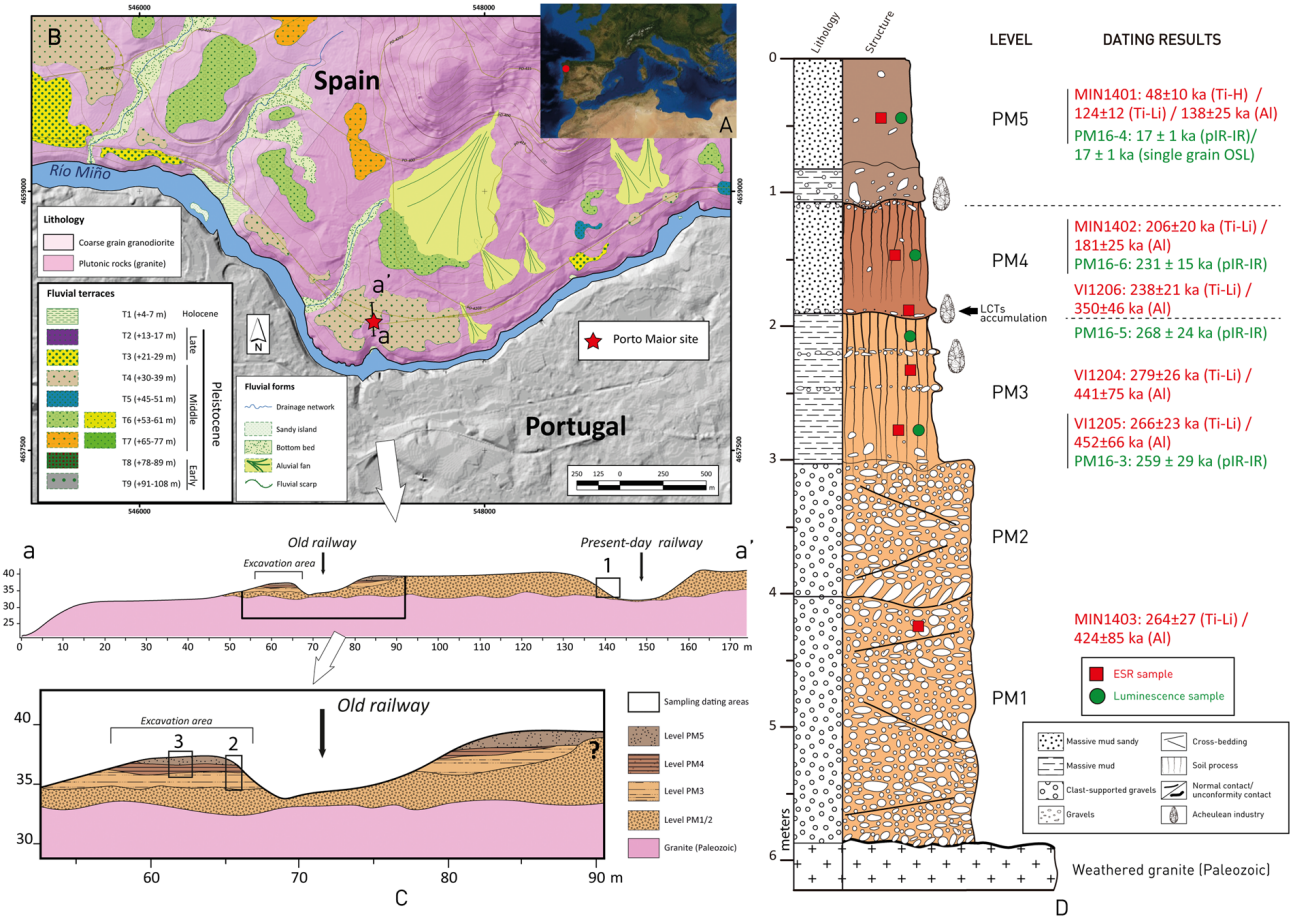
(**A**) Geographical location of Porto Maior. (**B**) Geomorphologic position of the site in relation to the terrace system of the lower Miño River basin. (**C**) Stratigraphic schematic section of the site and its relation with the T4 (+34 m) fluvial terrace deposits. The numbers represent the sampled zones. (**D**) Stratigraphy of the Porto Maior site with the main archaeological levels, as well as the ESR and luminescence dating results. This map was created with software ArcMap 10.4.1 and Adobe Illustrator CC.

Porto Maior site (As Neves, Pontevedra) is located in the Miño River basin and corresponds to a >6 m-thick fluvial terrace (T4 terrace in the regional fluvial sequence) located +34 m above the current level of the Miño River (Figs 1 and S2). The fluvial sequence rests on chemically weathered granitic bedrock. In total, 5 stratigraphic levels have been identified and numbered from the bottom upwards as PM1 to PM5 (see *SI Geomorphologic and stratigraphic details*). Of these levels, PM1 and PM2 are purely fluvial facies consisting of clast-supported gravels (quartzites, quartzes and weathered granitic pebbles). Levels PM3 and PM4 are fluvial units with massive facies of silts and sands (overbank environment), both are affected by pedogenesis. PM4 sits unconformably on PM3 and has an accumulation of LCTs at its base, which is anthropic in origin. The base of PM5 comprises a colluvium gravel deposit with re-deposited lithic industry. In contrast, the upper section of PM5 is composed of fine loam sediments of aeolian origin (Figs 1 and S3).

### Chronological framework

Chronologies for the Porto Maior site were obtained using a combination of electron spin resonance (ESR) dating of optically bleached quartz^25,26^ and post infrared-infrared stimulated (pIR-IR) luminescence dating applied to K-rich feldspar grains^27,28^. In addition, optically stimulated luminescence (OSL) dating of individual quartz grains^29^ was applied to the uppermost unit preserved at the site (PM5). The lack of preserved organic and faunal remains precluded the use of other numerical and relative dating techniques. A total of 6 ESR and 4 luminescence dating sediment samples were collected from deposits bracketing the main LCT accumulation; 9 samples were taken from the archaeology-bearing upper section of the sediment sequence (PM3-PM5) and one sample was collected from the basal unit PM1.

In accordance with the Multiple Centre (MC) approach^26^, two replicate ESR ages (Al and Ti-Li centre ages) were obtained for each quartz sample from units PM1 to PM4 (Fig. 1). The Al centre ESR ages range from 181 ± 25 ka to 452 ± 66 ka and, with the exception of sample MIN1402, are systematically older than the corresponding Ti-Li centre ESR ages, which fall within a much narrower range from 206 ± 20 ka to 279 ± 26 ka (*SI ESR dating of optically bleached quartz grains*). Out of the two replicate ESR datasets, the ages obtained for the more rapidly and completely bleached Ti-Li centre signals are considered to provide the most reliable burial estimate for the deposits, while the ESR ages obtained using the more slowly and incompletely bleached Al centre signals are interpreted as providing a maximum chronology^26^. In comparison, the three pIR-IR ages obtained for units PM3 and PM4 range from 231 ± 15 ka to 268 ± 24 ka. These bracketing ages for the *in situ* LCT accumulations are statistically indistinguishable at 1σ and are consistent with the paired Ti-Li ESR ages for units PM3 and PM4, suggesting relatively rapid sedimentation rates for these deposits. Consequently, a weighted mean ESR and luminescence age of 268 ± 12 ka may be derived for PM3 and the lower archaeological level. A second weighted mean ESR and luminescence age of 226 ± 10 ka may be obtained for PM4 and the associated archaeological level.

Unit PM5, which is devoid of *in situ* artefacts, yielded non-overlapping pIR-IR and Ti-Li centre ESR ages of 17 ± 1 ka and 124 ± 12 ka, respectively. Unlike the other samples, an additional ESR age of 48 ± 10 ka was obtained for MIN1401 using the Ti-H centre, which is usually considered to provide a more appropriate ESR dating signal for Late Pleistocene samples^30^. This Ti-H age is significantly younger than the Ti-Li and Al centre ESR ages for unit PM5, but it remains systematically higher than the corresponding pIR-IR age. Although incomplete bleaching of the Ti-H signal cannot be excluded for this sample, the observed difference in ages might simply be explained by the fact that the ESR and luminescence samples were collected from two different zones within the excavation area. The latter was positioned closer to the surface and might represent a younger depositional event. However, given the potential complications of applying ESR quartz dating over low dose ranges of <100–200 Gy^25,26^, we consider the pIR-IR age of 17 ± 1 ka to provide a more accurate depositional constraint for unit PM5. The reliability of this pIR-IR age is further supported by a replicate single-grain OSL age of 17 ± 1 ka for the same sample.

The combined chronological results obtained using different methods and minerals reveal a coherent and robust model of deposition for the *in situ* LCT accumulations at Porto Maior. The bracketing sedimentary units of PM3-PM4 were deposited between 293–205 ka (2σ confidence interval of the weighted mean ages), during the latter part of MIS 8 and the beginning of MIS 7 (Fig. 1). The basal unit of the sediment sequence (PM1) was also deposited during MIS 8, which is compatible with the geomorphological position of the fluvial terrace (T4 + 30–39 m) in this portion of the valley. The younger (MIS 2) age for unit PM5 is consistent with a major change in sedimentary processes between PM4 and PM5, and potentially indicates the presence of a prolonged sedimentary hiatus during the Late Pleistocene.

### The LCT accumulation of level PM4

**Figure 2:**
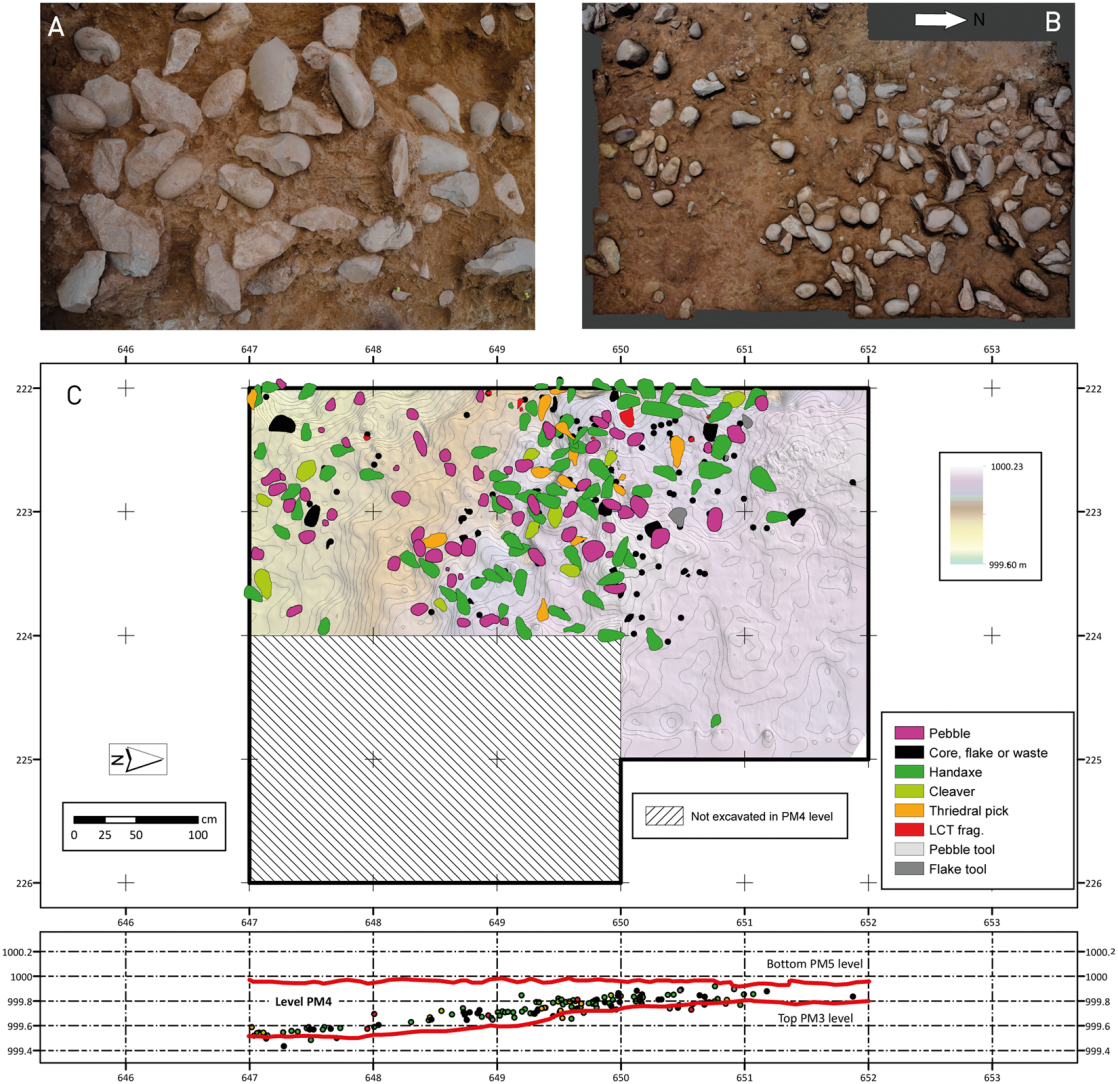
(**A**) Detail of the main archaeological concentration in level PM4. (**B**) Partial photogrammetric model of LCTs accumulation. (**C**) Distribution of the main assemblage (MA) lithic industry and stones. The main technological categories are differentiated. This map was created with software ArcMap 10.4.1 and Adobe Illustrator CC.

**Figure 3:**
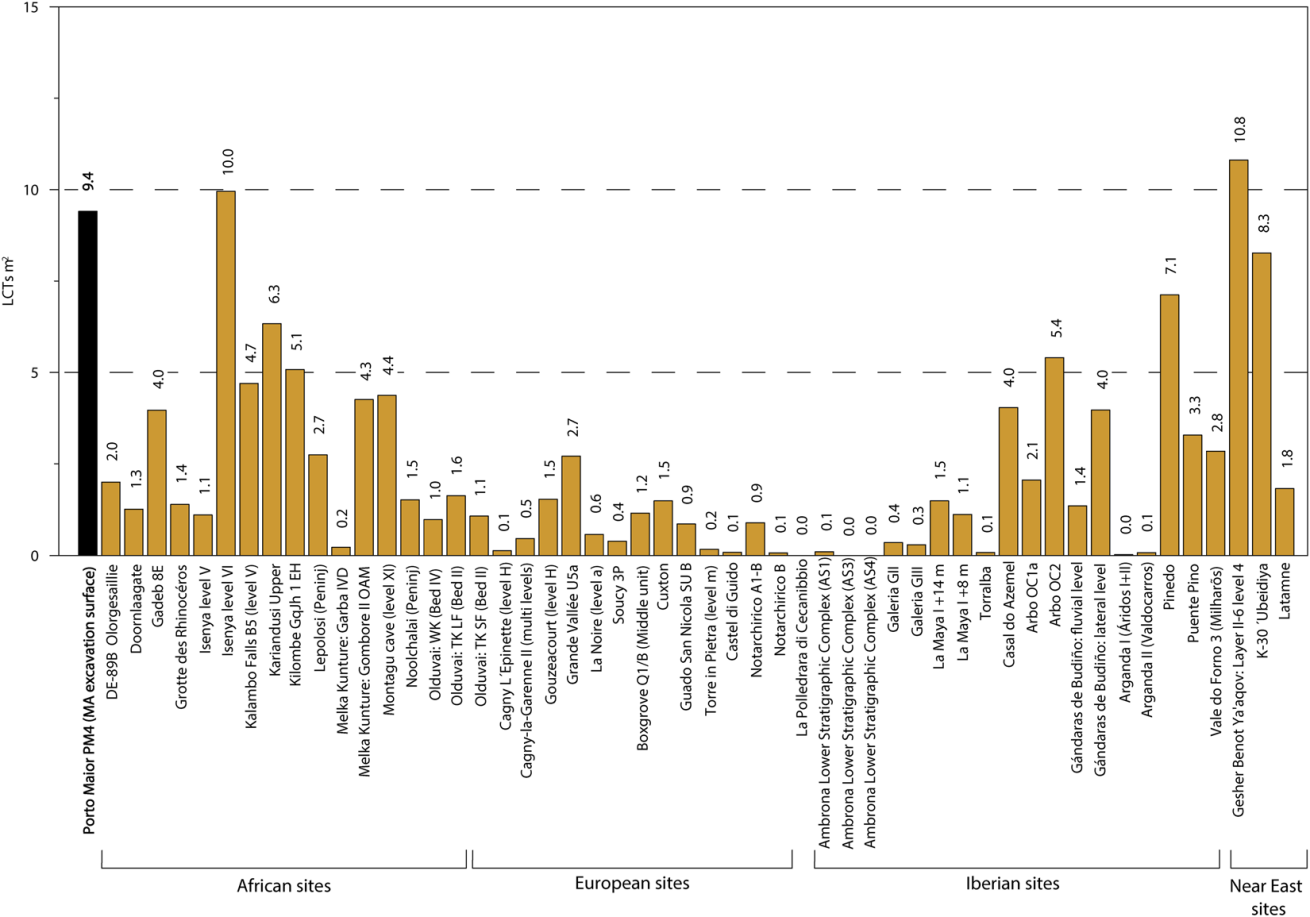
Density of LCTs for different Acheulean sites from Africa, Near East and Europe (see references in *SI Overview of extra-European sites with large LCT accumulations*).

A total of 3698 lithic artefacts were recovered from the site as part of excavations and superficial prospection (3077 and 621 pieces, respectively). Most of the recovered material from the 17.5 m^2^ excavation area came from the upper level PM5 (2614 pieces), but these are redeposited Acheulean materials in allochthonous position. In the lower level of PM3, a small assemblage of lithic industry was recovered (161 pieces), also with Acheulean characteristics. The 11.8 m^2^ excavated area in level PM4 produced 290 lithic artefacts and exposed a large concentration of Large Cutting Tools (LCTs) with an inhomogeneous distribution through the excavated surface (Fig. 2). From these 290 implements we distinguish 159 pieces that do not have fluvial abrasion (main assemblage; MA), while 131 pieces have fluvial abrasion (fluvial rolling assemblage; FRA) (*SI Site formation*). These taphonomic findings indicate that the MA material is most likely in an autochthonous position and not the result of fluvial transportation. This interpretation is further supported by the lack of a preferred orientation for the MA material (Fig. S14b, Table S14), as well as the dominant size range (Figs S15 and 16) and the preserved spatial patterns (Fig. S17). Consequently, the MA assemblage, which essentially comprises handaxes, cleavers and trihedral picks, is considered to represent *in situ* evidence of human activities at this level. The density of the MA is 13.4 pieces per m^2^, which appears to be relatively high in comparison to other Iberian Acheulean sites in fluvial environments^4^. The density of LCTs in level PM4 at Porto Maior is 9.5 pieces per m^2^, and represents one of the highest densities of LCTs recorded on a global scale (Fig. 3, Table S18). These high lithic densities appear to reflect the types of human activities undertaken at the site, and are reinforced by the enlarged excavation surfaces considered in this study.

### The lithic industry of the MA collection

The MA material is made up of 159 pieces with a total weight of 118.1 kg. The techno-typology is dominated by LCTs (69.8% of the pieces and 83.0% of the total weight). All other technological categories have limited representation with 17.0% of flakes, 5.0% of waste, 4.4% of cores and 3.1% of flake tools. None of the pieces have macroscopic signs of percussion that could be interpreted as hammer or anvil stones (Table 1).

**Table 1:** Classification of lithic implements from PM4 level.

		FLUVIAL ALTERATION					Total
			R0	R1	R1–2	R2	
LITHIC IMPLEMENTS	“*Manuport*”	*n*	0	0	1	0	**1**
		%	*0.0*%	*0.0*%	*100.0*%	*0.0*%	*0.3*%
	Flakes	*n*	27	39	33	1	**100**
		%	*27.0*%	*39.0*%	*33.0*%	*1.0*%	*34.5%*
	Waste	*n*	8	16	5	1	**30**
		*%*	*26.7%*	*53.3%*	*16.7%*	*3.3%*	*10.3%*
	Cores	*n*	7	6	3	0	**16**
		*%*	*43.8%*	*37.5%*	*18.8%*	*0.0%*	*5.5%*
	Flake tools	*n*	5	5	10	0	**20**
		*%*	*25.0%*	*25.0%*	*50.0%*	*0.0%*	*6.9%*
	Handaxes	*n*	80	5	1	0	**86**
		*%*	*93.1%*	*5.8%*	*1.2%*	*0.0%*	*29.7%*
	Cleavers	*n*	9	2	2	0	**13**
		*%*	*69.2%*	*15.4%*	*15.4%*	*0.0%*	*4.5%*
	Trihedral picks	*n*	12	0	0	0	**12**
		*%*	*100%*	*0.0%*	*0.0%*	*0.0%*	*4.1%*
	Choppers	*n*	1	0	0	0	**1**
		*%*	*100.0%*	*0.0%*	*0.0%*	*0.0%*	*0.3%*
	LCT fragments	*n*	10	0	1	0	**11**
		*%*	*90.9%*	*0.0%*	*9.1%*	*0.0%*	*3.8%*
**Total**		*n*	**159**	**73**	**56**	**2**	**290**
		*%*	*54,8%*	*25.2%*	*19.3%*	*0.7%*	*100.0%*

The material is organized according to their state of conservation. The R0 material constitutes the MA, while the R1, R1–2 and R2 belong to the FRA.

The selected raw material was almost exclusively quartzite (91.9%). A reduced number of tools were identified as being knapped on quartz, and these were composed essentially of flakes and waste (n = 13). Quartzite and quartz cobbles are abundant in the Miño River fluvial load and represent the likely raw material source for the site.

The “*chaînes opératoires*” analysis shows that one set of pieces is characterised almost exclusively by elements related to the use and discard phase (LCTs and some flake tools). The number of pieces associated with the acquisition and production phases –flakes, waste or cores- is insignificant (Table 1). The site contains a large number of LCTs, without elements pertaining to the configuration process (flakes, large blanks or cores). These characteristics suggest that the macro-tools were configured elsewhere and were brought to the site for usage and subsequent abandonment. The provenance of 41 pebbles, with a mean length of 136 mm, whose presence in this sedimentary context does not have a geological explanation, is yet to be determined.

The high frequency of handaxes (n = 80; 65.0%) in relation to trihedral picks (n = 12; 9.7%) and cleavers (n = 9; 7.3%) is noteworthy. LCTs fragments (8.1%), flake tools (4.0%) and choppers (0.8%) have a particularly reduced presence. Six of the flakes can be related to maintenance of the LCTs. In four cases, these flakes correspond to “*éclat de taille de biface”* and in two cases they correspond to “*coup de tranchet”* flakes, but they cannot be reassembled with any of the identified LCTs (*SI Technological features of the LCTs implements*).

**Figure 4:**
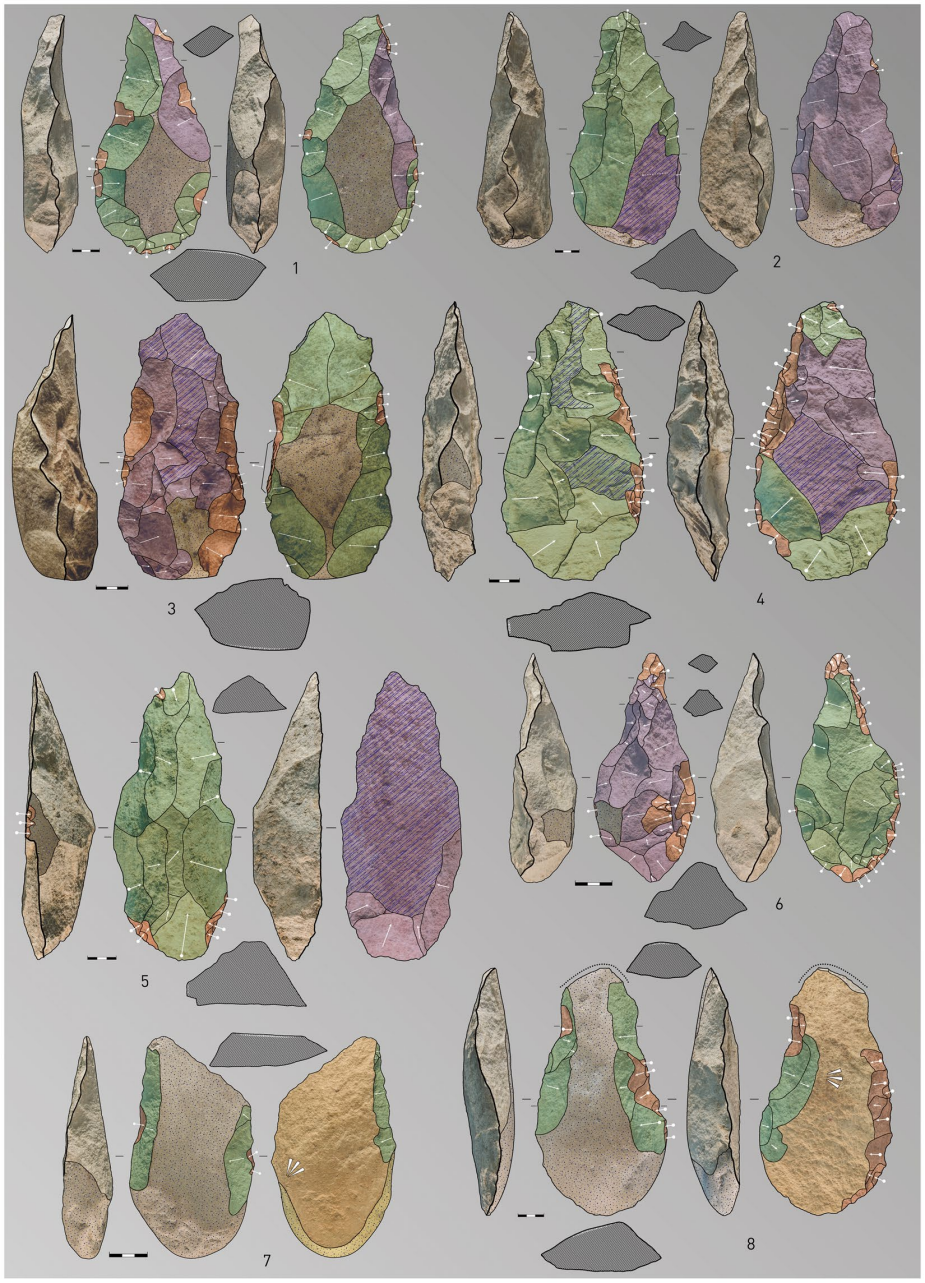
Examples of LCTs recovered from level PM4 at Porto Maior, 1–4: handaxes, 5–6: trihedral pick and 7–8: cleavers (each scale-bar represents 3 cm). See legend in Fig. S20 for an explanation of the colour scheme. Drawing and photo by E. Méndez-Quintas.

**Figure 5:**
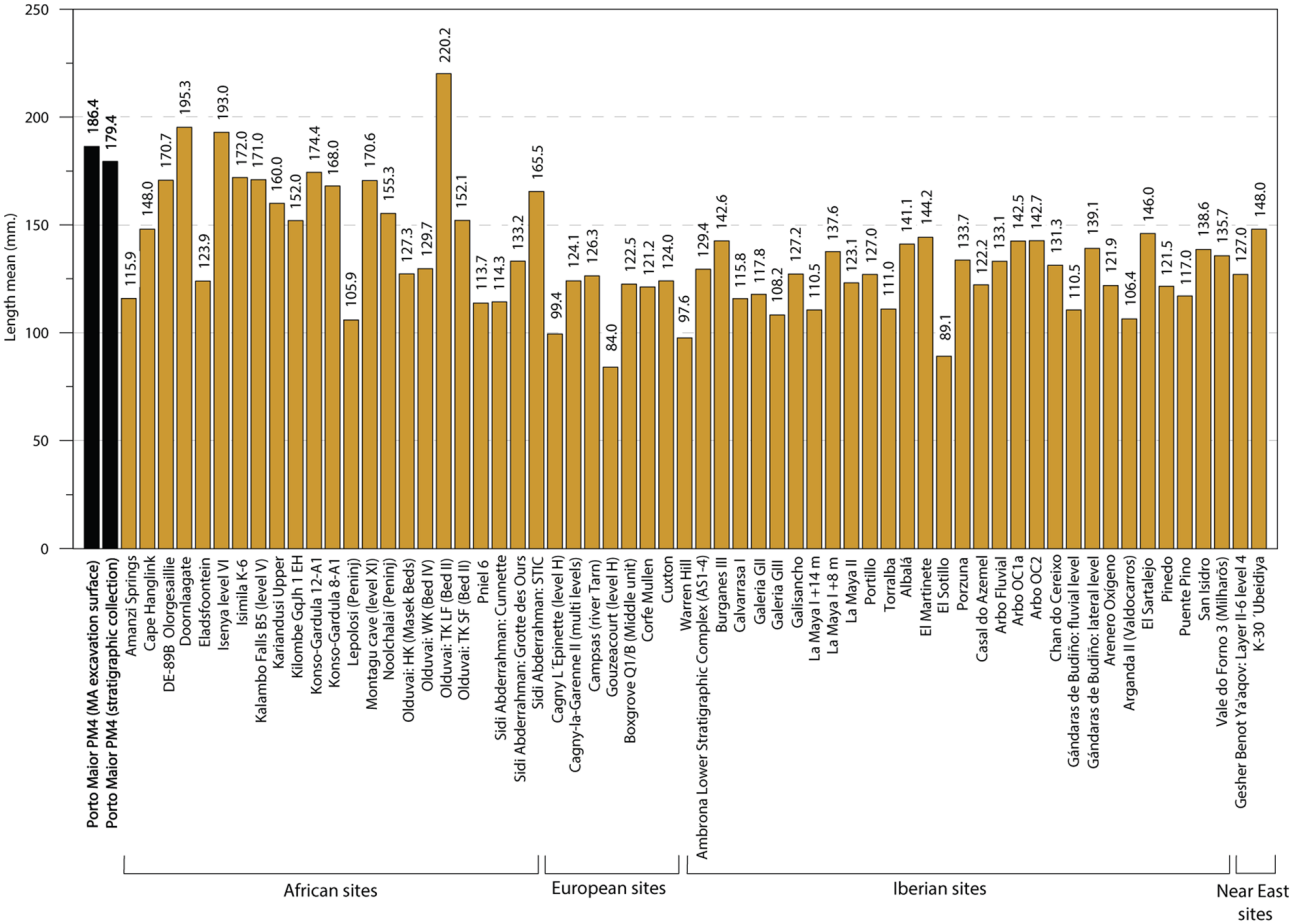
Mean length for different Acheulean handaxe assemblages from Africa, Near East and Europe (see references in *SI Overview of extra-European sites with large LCT accumulations*).

**Figure 6:**
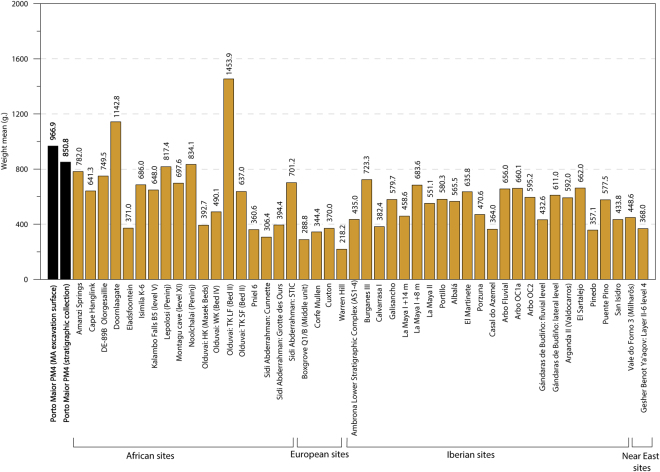
Mean weight for different Acheulean handaxe assemblages from Africa, Near East and Europe (see references in *SI Overview of extra-European sites with large LCT accumulations*).

The identified handaxes (n = 80), which are all on quartzite, are the most frequent LCT. Common morphologies include lanceolates (43.5%), amygdaloids (27.5%) and transverse cutting edge (15%)^31^. Other types of morphologies (oval or triangular) are very limited in occurrence (Fig. 4. 1–4; Figs S18–20). The frequency of cleavers is significantly less compared to handaxes (ratio of handaxes to cleavers is 8:1). Seven cleavers (77.8%) were assessed as being type O, one as type I (11.1%)^32^ and one with intermediate characteristics between type O and I (11.1%) (Fig. 4. 7–8). Trihedral picks are present in abundance, more so than cleavers, with 12 pieces shaped in quartzite (Fig. 4. 5–6). These LCTs are larger than the macro-tools from other assemblages in Iberia and Europe; their mean size is more similar to those measured on LCTs from African and Near Eastern collections (Figs 5 and 6). At present, the LCTs of Porto Maior level PM4 of Porto Maior represent the largest lithic artefacts found on the European continent (Figs 5 and 6*;* Table S18).

### Use-wear analysis of LCT implements

Preliminary analysis of the functionality of the LCTs has revealed additional evidence for use-wear. For 13 of the 101 LCTs (12.9%) from the MA collection, we have identified edge damage related to micro-flakes, scarring and rounding. The most significant alterations are damage to the distal edges of some cleavers (Fig. S23). For four of the handaxes, we observe traces of scarring and rounding, mainly on their distal edges. The same patterns are also observed in two trihedral picks, with some micro-flakes damages on their points. However, further use-wear studies are needed on the Porto Maior assemblage to advance our understanding of site functionality.

This type of use wear has been identified elsewhere for tools made of similar raw materials, and has been linked with the processing of hard material, mainly wood or bone, as well as the breakage of carcasses^33–35^. The presence of use-wear suggests that these LCTs were used for specific activities developed on site, before their subsequent abandonment.

## Discussion

The LCTs identified in Iberian and European Acheulean sites consistently have smaller sizes and weights than their African counterparts. In contrast, the technical features and size of the LCTs at Porto Maior (mean length = 186.4 mm) show strong parallelisms with Plio-Pleistocene African Acheulean collections (Figs 5 and 6*;* Table S17). Similarly, the density of tools recorded in Porto Maior level PM4 at Porto Maior is one of the highest globally for this type of occupation (9.5 LCTs/m^2^) and closely matches the densities reported for LCT accumulations in Africa and the Near East (Fig. 3; *SI Overview of extra-European sites with large LCT accumulations*). European sites with large numbers of handaxes are common (e.g., the different sectors at Boxgrove^36–38^, Soucy 3P^39^ and Grande Vallée^40^), but these sites have lower densities of archaeological finds; in part because they are usually excavated over extensive areas and are associated with a high number of knapping products and faunal remains. The highest densities of LCTs recorded in Iberia are Casal do Azemel^41^ and Pinedo^42^, although the latter has a significant component of re-deposited material and its LCT accumulation has been derived from a thick (>5 m) vertical sedimentary sequence. In the vast majority of SW European sites, however, the density of LCTs is clearly lower than those observed in African and Near Eastern sites. Therefore, Porto Maior stands out as the first archaeological site in Europe (Fig. S25) for which both the technological features of the Acheulean LCT industry and the type of tool accumulation displays unequivocal affinities with the Acheulean technocomplex of Africa.

The large size of the LCTs at Porto Maior could be explained by the corresponding large morphology and size of the existing pebbles in the fluvial load of the Miño River (Fig. S24). However, similarly large cobbles are common in other Iberian fluvial systems, which are devoid of comparable LCT assemblages^43,44^; hence it seems possible that the size of the PM4 LCTs is directly related to the functionality of the site. The interpretation of the function of such Plio-Pleistocene archaeological sites has generated vigorous academic debate over recent decades^45^. Most of the postulated hypotheses have been developed on sites associated with the first members of the genus *Homo*, and within pre-Acheulean African contexts. Sites with large accumulations of LCTs have received less attention, and have sometimes been considered to be the result of natural sedimentary processes^46–48^. However, a number of researchers have argued that these extensive LCT accumulations are the product of deliberate economic activities (mainly carcass consumption)^49,50^. Such economic activities can be diverse depending on the site^49–51^, as has become evident in recent excavations of the TK site in Olduvai^52,53^. The use-wear analysis undertaken on 13 LCTs from Porto Maior indicate that this site was likely used to process hard material, mainly wood and bones, as described elsewhere for a number of African or European sites^34,54^. This evidence is consistent with targeted economic exploitation of LCT resources at Porto Maior. It indicates that the functionality of this site parallels that of similar LCT sites in Africa, although the lack of faunal remains precludes further interpretation.

The age of the LCT accumulation at Porto Maior provides new insight into the spatial complexity of technological traditions existing across SW Europe during the Middle Pleistocene. The first human occupation of the Iberian Peninsula occurred more than 1 Ma^55–57^, and was characterised by non-Acheulean core and flake industries. Some researchers consider that the first Acheulean presence in the region is recorded at the site of La Boella (Tarragona), which has been dated to between 1.0 and 0.8 Ma^58^. However, the material recovered from this site comprises a limited assemblage and cannot be unequivocally assigned to the Acheulean technocomplex^3^. Another controversial example of the early Acheulean occupation of SW Europe comes from the site of Cueva Negra del Estrecho del Río Quipar (Murcia), which exhibits a series of stratigraphic, chronological and archaeological complications^15,20,59,60^. Further examples that may have broadly analogous chronologies include several small assemblages of lithic industry associated with fluvial terraces of the large rivers in the Iberian plateau at heights of more than +40 m^10,21^; some of which contain elements that would suggest an Acheulean attribution. Outside the Iberian Peninsula, several Acheulean sites older than 660–400 ka^9,17,61^ have also been reported. Recently, new chronologies have been published for a diverse number of Acheulean sites of “European facies” from NW Europe^9,18^. The numerical age control available for these sites would place the earliest Acheulean in this region and Italy to pre-MIS 12^9,17,18^. Importantly, however, the technological characteristics at these sites are different to those observed in SW Europe (Iberia and Aquitanian region), whose distinguishing features include the use of large flakes as blanks (LFB industries) for the LCTs and the presence of flake cleavers^4,5,23^.

The first widespread and unequivocal evidence for Acheulean technology in the south-western part of the European continent, including the Iberian Peninsula, emerges after MIS 13, and prevails until MIS 6, or even MIS 5^10,62,63^. In this interval of around 400 ka, the number of verified Acheulean sites increases exponentially over time^2,4^. The majority of these Acheulean sites are associated with the middle terraces of the large Atlantic basins (Duero, Tajo, Guadiana and Guadalquivir), which exhibit elevations of +40−20 m^4,10,21^, as well as a smaller number of cave and open-air sites not directly associated with specific fluvial terrace heights^4,21^. Dating of the archaeological deposits in these Iberian basins confirms an age range of 400–200 ka for the expansion of the Acheulean in the region^64–67^.

Within this geographic and chronological context, level PM4 of the Porto Maior site is the only known European example of an LFB Acheulean accumulation with pronounced African affinities. The occupation age range of 293–205 ka for Porto Maior is consistent with that obtained for “non-African” Acheulean assemblages from middle terrace (+40−20 m) sites of the large Iberian rivers^3,10,62,68^. It is apparent, therefore, that different LFB Acheulean assemblages were being simultaneously exploited by different geographic populations of SW Europe during the late Middle Pleistocene. Adding to this technological complexity is the fact that the age of Porto Maior level PM4 also overlaps with sites displaying Early Middle Paleolithic (EMP) industries, which are technologically different and not directly related to the LFB Acheulean^2,3,69–71^. Broadly contemporaneous EMP sites in Iberia include the middle stratigraphic unit of Ambrona, Level TD10.1–2 at Gran Dolina (Atapuerca), Bolomor Cave and Cuesta de la Bajada^3,72,73^. Our dating study of the LCT accumulation at Porto Maior provides a new line of evidence for temporal overlap between these two techno-complexes in SW Europe^3,74^.

These chronological findings have important implications for understanding the complex human occupation history of the continent. It is increasingly clear from the palaeoanthropological record that a mosaic of human populations of different geographic origins, and with varied biological and technological attributions, likely co-existed in Europe at various times during the Middle Pleistocene^75–77^. The extensive LCT assemblage at Porto Maior contrasts with those of contemporaneous LFB Acheulean sites and EMP sites in the region, and similarly suggests the co-existence of culturally distinct human populations of different geographical origins. In this sense, the African affinities of the LCT assemblage at Porto Maior may be consistent with a technology brought in by an “intrusive” population, which differed from the core and flake industries of established human groups in SW Europe.

The archaeological site of Porto Maior (Level PM4) represents the first European site characterised by an extensive concentration of *in situ* LCTs with similar technological and occupation characteristics as those found exclusively in Acheulean sites from Africa and the Near East. At present, the LCTs at Porto Maior are among the largest lithic artefacts found on the European continent. The accumulation layer is within an overbank facies that was deposited with insufficient energy to displace large lithic tools in a significant way. This geomorphic context, and the lack of a preferred orientation for the MA material, supports the anthropic origin of the LCT assemblage. The two independently derived chronological datasets (ESR and luminescence) for Porto Maior provide consistent ages and indicate that these deposits accumulated sometime between 293–205 ka, though the exact duration of site occupation is difficult to resolve beyond our existing dating uncertainties. Use wear analysis of the MA material shows that the LCTs were used for the processing of hard materials, such as bone and wood, or for the processing of carcasses. These results indicate that tool accumulation was likely the result of specific activities undertaken on-site, and was not merely the product of re-deposition via natural sedimentary processes.

The unique archaeological findings from Porto Maior provide a new line of evidence to support the relationship between the Iberian and African Acheulean industries. Until now, this affinity has been exclusively founded on technological similarities. For the first time, the cultural connection between the two continents can be extended to include the type of occupation site (extensive LCT accumulations), thereby providing additional insight into the origin of the European Acheulean technology. The MIS 8-7 chronology obtained for Porto Maior sequence confirms the co-existence of the LCT Acheulean and EMP industries in SW Europe^3,9,74,78^. This situation would potentially suggest the co-existence of different human species in southwest Europe during the Middle Pleistocene; a scenario also reflected by emerging palaeoanthropological evidence from European fossil hominin sites^75,79–81^.

## Methods

### Archaeology and stratigraphy

The archaeological investigations of Porto Maior were undertaken over two seasons in 2012 and 2014, and consisted of excavations, stratigraphic analyses and the collection of dating samples from the excavated levels. Likewise, geomorphologic work was undertaken to provide the geological context of the site at a regional scale. These studies were based on the detection and description of fluvial forms using digital elevation models (DEM) derived from LIDAR data. Archaeological materials were studied by means of taphonomic, technological, typological and metric criteria. The maps of the site were obtained from a combination of topographic and photogrammetry of close object techniques. Data were processed using GIS software, which produced high-precision cartographies and three-dimensional reconstructions. The use-wear analyses included the observation of the cutting edges, which was made using a binocular stereomicroscope Motic SMZ171. Images were taken with a microscope adapter and Nikon D7000 photographic camera with objective lens Sigma 105/2,8 DG Macro OS.

### Geochronology

ESR dating of quartz grains was carried out at CENIEH (Spain) following the Multiple Centre approach as described in^25^. Quartz grains were dated using the standard Multiple Aliquots Additive (MAA) dose method. Each natural sample was divided into several multiple-grain aliquots, which were gamma irradiated up to ca. 40 kGy. For each sample, the ESR signals of both the Aluminium (Al) and Titanium (Ti) centres were obtained from repeated measurements at low temperature. Equivalent Dose values were obtained for each centre in order to check their consistency, and evaluate any possible incomplete bleaching of the Al signal during sediment transportation, as described in^25^. The total dose rate value was derived from a combination of *in situ* and laboratory measurements. External gamma dose rate was derived from *in situ* measurements with a NaI probe by using the “threshold technique”^82^. Radioelement (U, Th, K) concentrations of the sediment were determined by ICP-MS analysis and used to derive external alpha and beta dose rate components using the dose rate conversion factors from^83^. Additional High Resolution Gamma Spectrometry (HRGS) analysis did not show evidence of significant disequilibrium in the U-238 decay chain. Internal dose rate was assumed to be 50 ± 30 uGy/a, based on previous works. Values were corrected with beta and alpha attenuation values for spherical grains^84,85^ and water attenuation formulae from^86^. ESR and luminescence age calculations were performed using consistent water content values: 20 ± 5% (wet weight) was considered for all but one ESR sample, and 13 ± 5% for the uppermost ESR sample. The cosmic dose rate was calculated using formulae from^87^, with depth, altitude and latitude corrections. ESR age calculations were performed using a non-commercial SCILAB based software, and final errors are given at 1σ error. Full details about the experimental conditions and the analytical procedure are provided in Supplementary Information.

Luminescence dating was conducted at the University of Adelaide’s Prescott Environmental Luminescence Laboratory. Luminescence dating samples were collected from levels PM3, PM4 and PM5 at Porto Maior. Post infrared (IR) IR stimulated luminescence (pIR-IR) dating of coarse K-rich feldspar grains was applied to all samples^27,28^. Additionally, single-grain optically stimulated luminescence (OSL) of quartz grains^29^ was applied to sample PM16-4 from PM5. Samples were prepared using standard procedures under subdued red lighting to isolate the coarse (90–125 μm) K-rich feldspar and (212–250 μm) quartz fractions (see *SI Luminescence dating* for details). Dose recovery tests were performed for the pIR-IR signal using the two protocols outlined in Table S7 and the rejection criteria described in^88^ (*SI Luminescence dating*). The elevated temperature IR measurements made at 225 °C (pIR-IR_225_ protocol) produced a dose recovery ratio of 0.97 ± 0.02 in agreement with unity at 1σ (Table S8), while the elevated temperature IR measurements made at 290 °C (pIR-IR_290_ protocol) resulted in dose overestimation (dose recovery ratio = 1.29 ± 0.04). Therefore, the pIR-IR_225_ protocol was used to date the samples in this study (see *SI Luminescence dating* for details). In total, 6 to 7 160-grain aliquots of K-feldspars were measured for each sample. The natural D_e_ datasets exhibited low overdispersion values (5–22%) and were not significantly positively skewed, indicating full bleaching of the dating signal prior to burial. Mean D_e_ values and final burial ages were calculated using the central age model^89^. Dose rate evaluations were undertaken using a combination of *in situ* gamma-ray spectrometry and low-level beta counting (*SI Luminescence dating* for details). Gamma spectrometry data was analysed using the ‘windows’ method described in^90^ to obtain concentrations of K, U and Th. Dose rates were calculated using the conversion factors of ^83^, accounting for beta attenuation^84^. For the fluvial samples (PM16-3, PM16-5 and PM16-6) the long-term water content was taken as being equivalent to 50% of the saturated values measured for each sample (*SI Luminescence dating*), which ranged between 20% and 29% of dry weight. For the aeolian sample (PM16-4), a long-term water content of 15 ± 5% was used following similar values adopted in other luminescence dating studies of wind-blown (loess) deposits from Europe (*SI Luminescence dating*). An uncertainty of 20% was added to the water content to account for any potential minor variations during the past. Final dose rates are shown in Table S5.

## Electronic supplementary material

Supporting Information
