# Supplementary material for: First evidence of an extensive Acheulean large cutting tool accumulation in Europe from Porto Maior (Galicia, Spain)
Source: Sci Rep. 2018 Feb 15;8:3082. doi: 10.1038/s41598-018-21320-1 (PMC5814561; doi:10.1038/s41598-018-21320-1)
Supplement: Supplementary file 1 — Supporting Information [file 41598_2018_21320_MOESM1_ESM.pdf]

Supporting Information

First evidence of an extensive Acheulean large cutting tool accumulation in Europe from Porto Maior (Galicia, Spain)

E. Méndez-Quintas[1,2*], M. Santonja[1], A. Pérez-González[1], M. Duval[3], M. Demuro[4], and L. J. Arnold[4]

[1] Centro Nacional de Investigación sobre la Evolución Humana (CENIEH). Paseo de Atapuerca, 3. 09002 Burgos, Spain.

[2] Escuela Interuniversitaria de Posgrado en Evolución Humana, Universidad de Burgos, Juan de Austria 1, 09001 Burgos, Spain

[3] Australian Research Centre for Human Evolution. Environmental Futures Research Institute, Griffith University, 170 Kessels Road Nathan, QLD 4111, Australia

[4] School of Physical Sciences, Environment Institute, and Institute for Photonics and Advanced Sensing (IPAS), University of Adelaide, North Terrace Campus, Adelaide SA 5005, Australia.

SI Geomorphologic and stratigraphic context

The Miño River is the most important hydrographic network in NW Iberia with an area of ~17,000 km^2 and a length of 350 km (Fig. S1). Within the lower Miño River basin, and in the surroundings of Porto Maior site, 9 stepped fluvial terraces have been identified, with the following (relative) heights above river level estimated during the summer: T1 (+4-7 m), T2 (+13-17 m), T3 (+21-29 m), T4 (+30-39 m), T5 (+45-51 m), T6 (+53-61 m), T7 (+65-77 m), T8 (+78-89 m) and T9 (+91-108 m) (Fig. 1, Fig. S2). These terraces were formed due to the effects of tectonic uplift and climatic fluctuations during the Plio-Pleistocene. The sedimentary deposits of these terraces are characterized by thick sequences made of clast-supported crudely-bedded gravels, mainly quartzite and quartzes with minor components of granites (*Gh* facies), with internal planar cross-bedded facies (*Gp*) or cross-bedding (*Gt*), that alternate with fine to very coarse sands or pebbles with planar (*Sp*) or cross-bedded (*Sp*) structures. Also recognized are fine-coarse Sm facies with massive structure or faint laminations, similar to *Fsm* facies of silts and muds with massive structure, occasionally displaying mud cracks (*Fm* facies). The facies architecture indicates that the fluvial style is of a braided river system, with deposits dominated by gravels [1,2].

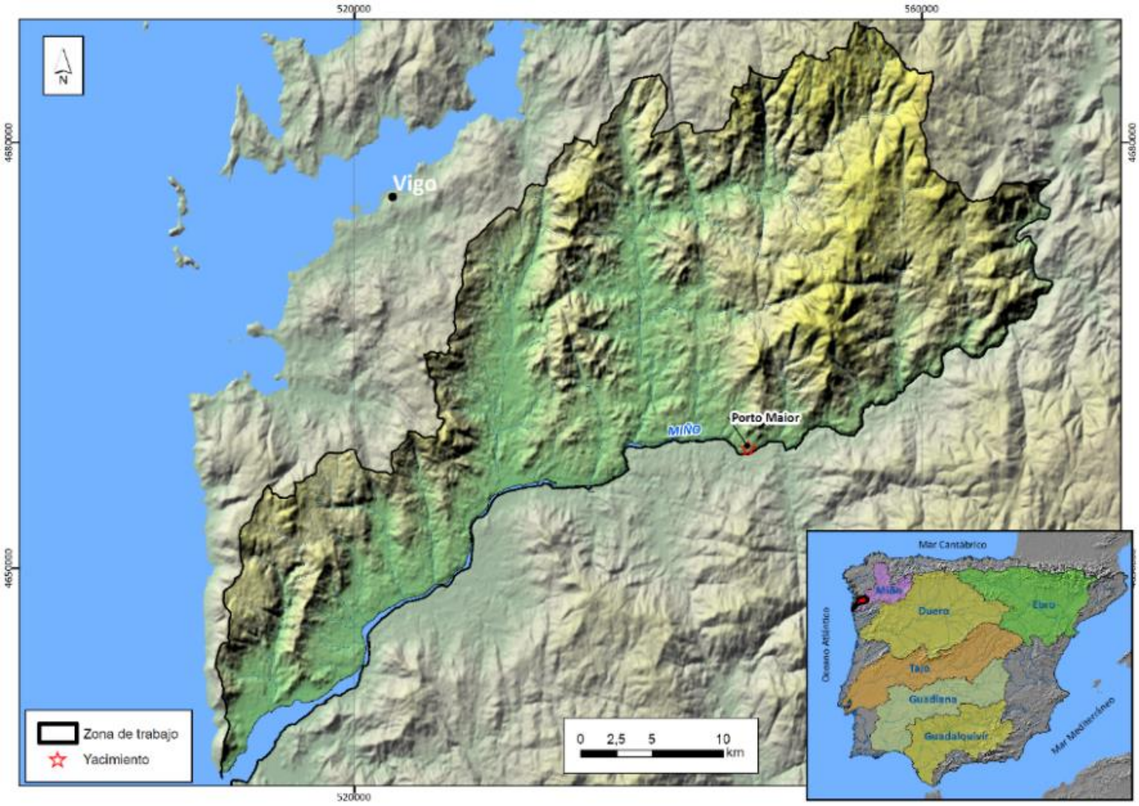

Fig. S 1. Site in the geographic context of the lower Miño basin. This map was created with software ArcMap 10.4.1.

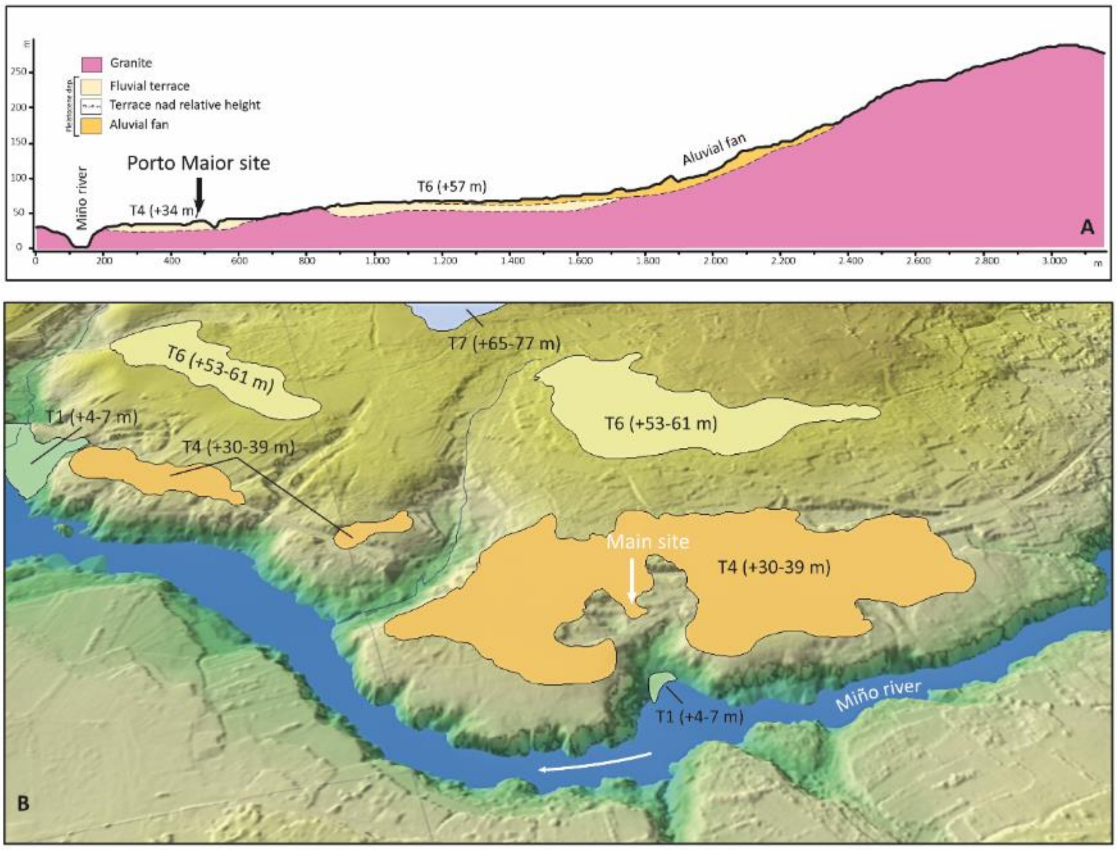

Fig. S 2. (A) Geomorphological profile with the position of the main fluvial terrace level in the surroundings of the Porto Maior site. (B) Main outcrop of fluvial terrace over a surface elevation model obtained from LIDAR data. This map was created with software ArcMap 10.4.1 and Adobe Illustrator CC.

In total, 5 stratigraphic levels have been identified at Porto Maior, named PM1 to PM5 from bottom to top. PM1 and PM2 are clast-supported gravels and most of the sand matrix fraction is coarse to very coarse (0.5-2mm) (Fig. S3). The clay fraction amounts to between 20 and 25 %, and has originated from processes of illuviation (horizon Bt) that developed after the sedimentation of level PM3. The colours of the sand-clay matrix are yellow (10YR 5/6) or reddish brownish (5YR 5/4). Level PM2 is more affected by reduction processes, and exhibits a pale yellow (2.5Y 7/4) and olive (5Y 5/6) colour. PM2 is a gravel facies *Gh* or *Gp* with horizontal planar stratification and clast imbrications. Level PM3 has a uniform textural composition, and is composed of muds with percentages of sand that range between 35 % and 40 %; although fine to median sand (between 0.5 and 0.05 mm) dominates. In these massive muddy deposits (Fsm and Fm facies), levels of low-thickness gravel are recognized associated with small flat channels, of limited lateral development. These channels would have traversed the overbank facies deposits, which in turn would have been affected by waterlogging and argilluviation

processes. The colours of this level are pale yellow (2.5Y 7/4) and yellowish red (5YR 5/6). Level PM4 has the same characteristics as the underlying unit (PM3). It is composed of a mud-sandy massive, with sand contents, which ranging between 42 % and 62 %, and being dominated by the fine to median grain size fraction. This sedimentary level is reddish yellow (7.5YR 6/6) and is also affected by a distinct illuviation process, with the development of a Bt horizon. Level PM5 is light yellowish brown (10YR 6/4). The base of the unit is composed of colluvium formed by heterometric gravels, while the upper sections of the unit are composed of silts (0.2-0.05 mm) of aeolian origin.

Our chronological results suggest that the processes of illuviation affecting the stratigraphic sequence of Porto Maior took place during the MIS 7 interglacial. The oldest of the processes of clay illuviation that affects PM1, PM2 and PM3, might correspond to isotopic sub-stage 7e. The second phase of illuviation affecting horizon PM4 likely took place during isotopic event 7c, during the second half of MIS 7 (isotopic sub-stages by [3]). The aeolian deposits at the top of PM5, were deposited during MIS 2, corresponding approximately with the decline of the Last Glacial Maximum (LGM). This first correlation attempt should, however, be considered with caution in the absence of additional high resolution proxies and given the existing dating uncertainties.

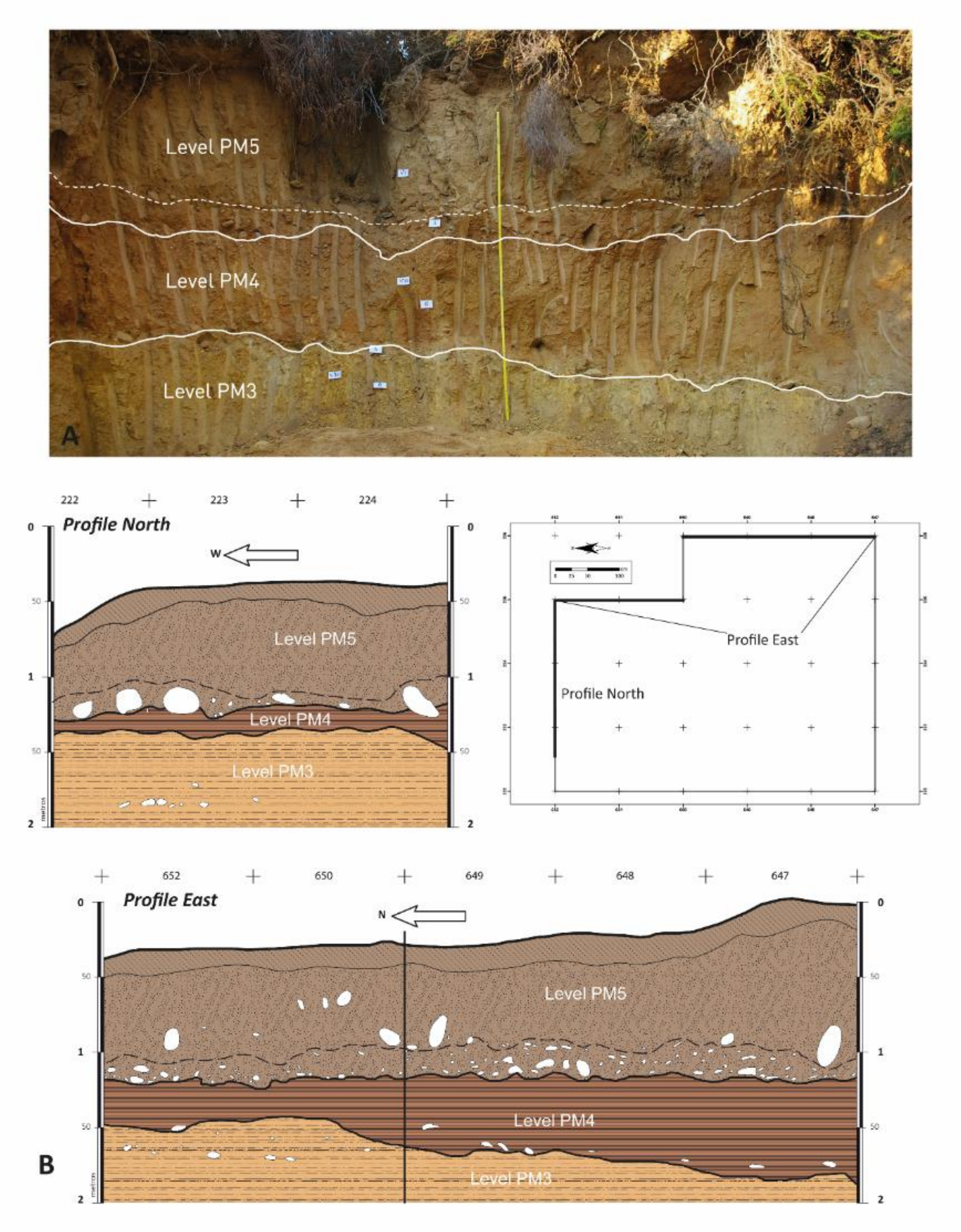

Fig. S 3. (A) Detail of the upper sequence showing levels PM3 to PM5. (B) Stratigraphic profiles in the excavation area. The concentration of LCT is located in the lower part of level PM4, above PM3. Drawing and photo by E. Méndez-Quintas

SI ESR dating of optically bleached quartz grains

Material

Several sediment samples were collected from Porto Maior site in two different fieldwork campaigns (Fig. S4): 3 samples (VI1204, VI1205 and VI1206) in October 2012, and 3 more samples (MIN1401, MIN1402 and MIN1403) during summer 2014. MIN1403 was taken from PM1 unit at the base of the local sequence in an outcrop located near the actual railway (zone 1 in Fig. 1), i.e. a few hundreds of m North of the excavation area. In contrast, the other samples were collected in the vicinity (zone 3) or within (zone 2) the main excavation area (Fig. S4): VI1204 and VI1205 belong to PM3, VI1206 and MIN1402 to PM4 and finally MIN1401 was collected from the top part of PM5.

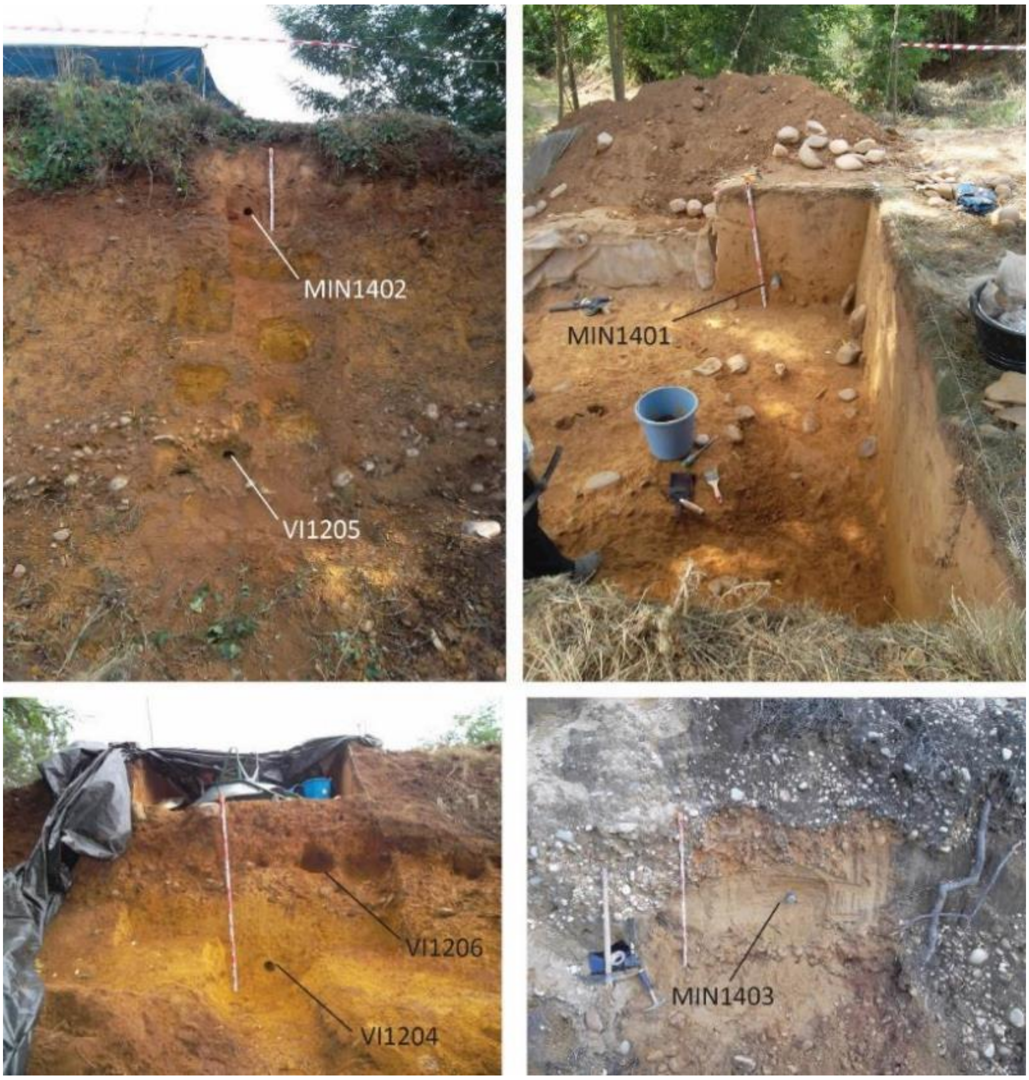

Fig. S 4. Pictures of the ESR sediment samples. MIN1403 was collected from an outcrop located close to the actual railway (zone 1 of Fig. 1). MIN1401, VI1204 and VI1206 was taken from the main excavation area, while MIN1402 and VI1205

were collected from a perpendicular outcrop, a few meters away from the main excavation area. Drawing and photo by M. Duval.

Methods

Sediment samples were prepared in the laboratory under conditions of limited illumination following the standard procedure at CENIEH [4]. The 100-200 µm size fraction was collected after wet sieving. HCl (36%) was used to dissolve carbonates and H_2O_2 (30%) to eliminate organic matter. Heavy minerals and feldspars were removed with Sodium Polytungstate solutions at d=2.72 and d=2.62 g/ml, respectively. Then, magnetic minerals were eliminated using neodymiun magnets. The resulting samples were treated with HF (40%) for 40 minutes to eliminate the remaining feldspars and to etch quartz grains. Finally, HCl (18%) was added in order to remove any soluble fluoride.

Quartz grains were dated by using the standard Multiple Aliquots Additive (MAA) dose method. Each natural sample was divided into 14 multiple grain aliquots. Twelve aliquots for each sample were irradiated using ^{137}Cs Gammacell-1000 source (dose rate = 6.90 Gy/min) to the following doses: 50.0, 100.1, 200.1, 400.3, 800.5, 1601.2, 3202.1, 6003.9, 10006.4, 17010.9, 27017.3 and 40025.7 Gy. One aliquot was kept unirradiated (natural aliquot), while the last aliquot was exposed to a SOL2 (Dr Hönle) solar light simulator for about 1440 h, in order to evaluate the non-bleachable residual ESR signals of the Aluminium centre.

ESR measurements were carried out at CENIEH (Burgos, Spain), with an EMXmicro 6/1Bruker X-band ESR spectrometer coupled to a standard rectangular ER 4102ST cavity. To ensure constant experimental conditions over time, the temperature of the water circulating in the magnet is controlled and stabilized at 18 ºC by a water-cooled Thermo Scientific NESLAB ThermoFlex 3500 chiller, and the temperature of the room is kept constant at 20 ºC by an air conditioning unit. ESR measurements were performed at low temperature (~90 K) using a ER4141VT Digital Temperature control system based on liquid nitrogen cooling. Further details about the setup and about its stability over time can be found [5].

In accordance with the Multiple Centre method defined by [6], the ESR signals of both the Al and Ti centres were measured. For the first one, the following acquisition parameters were used: 10 mW microwave power, 1024 points resolution, 20 mT sweep width, 100 kHz modulation frequency, 0.1 mT modulation amplitude, 40 ms conversion time, 10 ms time constant and 1 scan. In contrast, the ESR signal associated to Ti centre was measured as follows: 5 mW microwave power, 1024 points resolution, 20 mT sweep

width, 100 kHz modulation frequency, 0.1 mT modulation amplitude, 60 ms conversion time,10 ms time constant and 2 to 30 scans (depending on the aliquot). Each of the 14 aliquots (one natural, one optically bleached and eleven gamma irradiated aliquots) of a given sample were measured 3 times after a ~120° rotation in the cavity for both Al and Ti signals in order to consider angular dependence of the signal due to sample heterogeneity. The only exception was MIN1401: the very low ESR intensity of the Ti signals required a higher number of scans (up to 30), resulting in an already very long measurement time (>5 hrs). Consequently, no rotation were considered for this sample. Then, all measurements were repeated three times over distinct days in order to check the repeatability of the D_E values. Consequently, three sets of 13 data points were obtained for each signal measured (Ti and Al) in a given quartz sample.

The ESR intensity of the Al signal was extracted from peak-to-peak amplitude measurements between the top of the first peak (g=2.0185) and the bottom of the 16th peak (g=1.9928) [7]. Following the conclusions from [8], the ESR intensity of the Ti-Li centre was preferentially evaluated by measuring the peak-to-baseline amplitude around g=1.913-1.915 (option D). Peak-to-peak amplitude measurement between g=1.979 and the bottom of the peak at g=1.913 was also performed for comparison (option A). The intensity of the Ti-H centre was evaluated by taking the peak-to-baseline amplitude around g=1.915.

For each aliquot, ESR intensities of Al and Ti centres were corrected by the corresponding receiver gain value, number of scans, mass and a temperature correction factor [5]. The fitting procedures were carried out with the Microcal OriginPro 9.5 software using a Levenberg-Marquardt algorithm by chi-square minimization. For the Al centre, an exponential+linear function (EXP+LIN) was fitted through the experimental points (see equation in [4]), and data were weighted by the inverse of the squared ESR intensity ($1/I^2$). D_E values were obtained by extrapolating the EXP+LIN function to the residual intensity (so-called Total bleach method,[9]). For the Ti centre, we used the function labelled as Ti-2 in [8], in order to describe the non-monotonic dose dependence of the ESR signal at high doses. Data were weighted by the inverse of the squared experimental error ($1/s^2$) and D_E values were obtained by back extrapolation to the Y=0. For each sample, final dose response curves (DRCs) were obtained by pooling all the repeated measurements in a single plot, as recommended by [10].

The total dose rate value was derived from a combination of *in situ* and laboratory measurements. External gamma dose rate were derived from *in situ* measurements by using the “threshold technique” [11]. For each dated samples, the corresponding

radioelement (U, Th, K) concentrations in the sediment were determined by ICP-MS analysis of about 5g of dry raw sediment. In addition, ~150 g of this same raw sediment, previously dried and powdered, were analysed by High Resolution Gamma Spectrometry (HRGS) using a Canberra Extended Range (XTra) HpGe detector in order to identify possible disequilibrium in the U-238 decay chain. Concentration values were used to derive external alpha and beta dose rate components using the dose rate conversion factors from [12]. Dose rate values were calculated assuming a mean grain size of 150 µm, and an assumed thickness removed by HF etching of 20 µm. Internal dose rate was assumed to be 50±30 uGy/a, based on the work from [13] and assuming an alpha efficiency k of 0.15±0.10 [14]. Values were corrected with beta and alpha attenuation values for spherical grains [15,16] and water attenuation formulae from [17]. Current water contents were evaluated in the laboratory by drying the sediment at 50ºC in an oven during three weeks. Results vary within relatively narrow range from 8.5 to 13.2% (wet weight) among the samples. For a matter of consistency with the luminescence dating procedures, a value of 20±5% (wet weight) was considered in the ESR age calculation of all but one sample, which is equivalent to the 20-28% (dry wet) used for the OSL and pIR-IR samples. For the uppermost sample MIN1403 (unit PM1), a water content value of 13±5% was considered instead, which is also similar to the value of 15±5% (dry weight) adopted for the luminescence sample collected from this unit (PM16-4). The cosmic dose rate was calculated using formulae from [18], with depth, altitude and latitude corrections [19].

ESR age calculation were performed using a non-commercial SCILAB based software, with error calculations based on Monte Carlo simulations, considering the following sources of uncertainties: concentrations, depth, water content, gamma dose rate, beta dose attenuation, D_E values. ESR ages are given at 1σ error.

Results and Discussion

1.1. D_E determination

1.1.1. Al centre

ESR data and dose response curves (DRCs) derived from the measurement of the Al centre are provided in Table S1 and Fig. S5, respectively. Bleaching coefficients values vary between 35% and 50%, suggesting somewhat similar bleaching conditions for all samples. As a comparison, these values are lower than those obtained from other samples located within the Iberian Peninsula: at Cuesta de la Bajada or in the Alcanadre terraces, those values were around 55-60% instead [4,8].

Al centre												
				EXP+LIN function D_{max}=27 kGy			EXP+LIN function D_{max}=40 kGy			SSE function D_{max}=3.2 kGy		
Sample	Bleaching Coefficient (%)	Repeatability of the ESR intensities (%)	Repeatability of the D_E estimates (%)	Adjusted r^2	D_E value (Gy)	Reliability	Adjusted r^2	D_E value (Gy)	Reliability	Adjusted r^2	D_E value (Gy)	Reliability
VI1204	48.6±0.9	0.6%	9.7%	0.984	1544±232	Good	0.987	1658±217	Good	0.941	1377±252	Poor
VI1205	43.0±0.8	3.4%	27.8%	0.990	1491±185	Excellent	0.985	2146±237	Good	0.968	1561±222	Poor
VI1206	42.0±1.4	1.0%	11.6%	0.989	1241±130	Good	0.991	1247±119	Excellent	0.955	1182±186	Poor
MIN1401	36.1±0.8	2.0%	1.9%	0.981	484±81	Good	0.973	955±145	Regular	0.963	515±161	Poor
MIN1402	42.4±1.0	3.7%	11.8%	0.989	648±69	Good	0.983	907±92	Good	0.976	596±59	Regular
MIN1403	37.8±0.7	0.8%	8.4%	0.970	1671±308	Regular	0.971	2171±317	Regular	0.905	782±167	Poor

Table. S 1. ESR data derived from the measurement of the Al centre. Repeatability of the ESR intensities was assessed through the variability of the mean ESR intensity obtained after each day of measurements. Similarly, the repeatability of the D_E values corresponds to the variability of the D_E values calculated for each day of measurement. Following the criteria defined by [10], the reliability of the fitting results is assessed through the value of the adjusted r2: poor for $r^2<0.97$, regular for $0.97<r^2<0.98$, good if $0.98<r^2<0.99$, excellent for $r^2>0.99$.

The quality of the ESR data collected is within the standards in comparison with previous studies. Measurement repeatability for a given sample ranges from 0.6% (excellent) to 3.4% (regular). Consequently, 2 samples show an excellent or good D_E repeatability <10%, while 3 other samples are within 10-15% repeatability, and one sample above (VI1205).

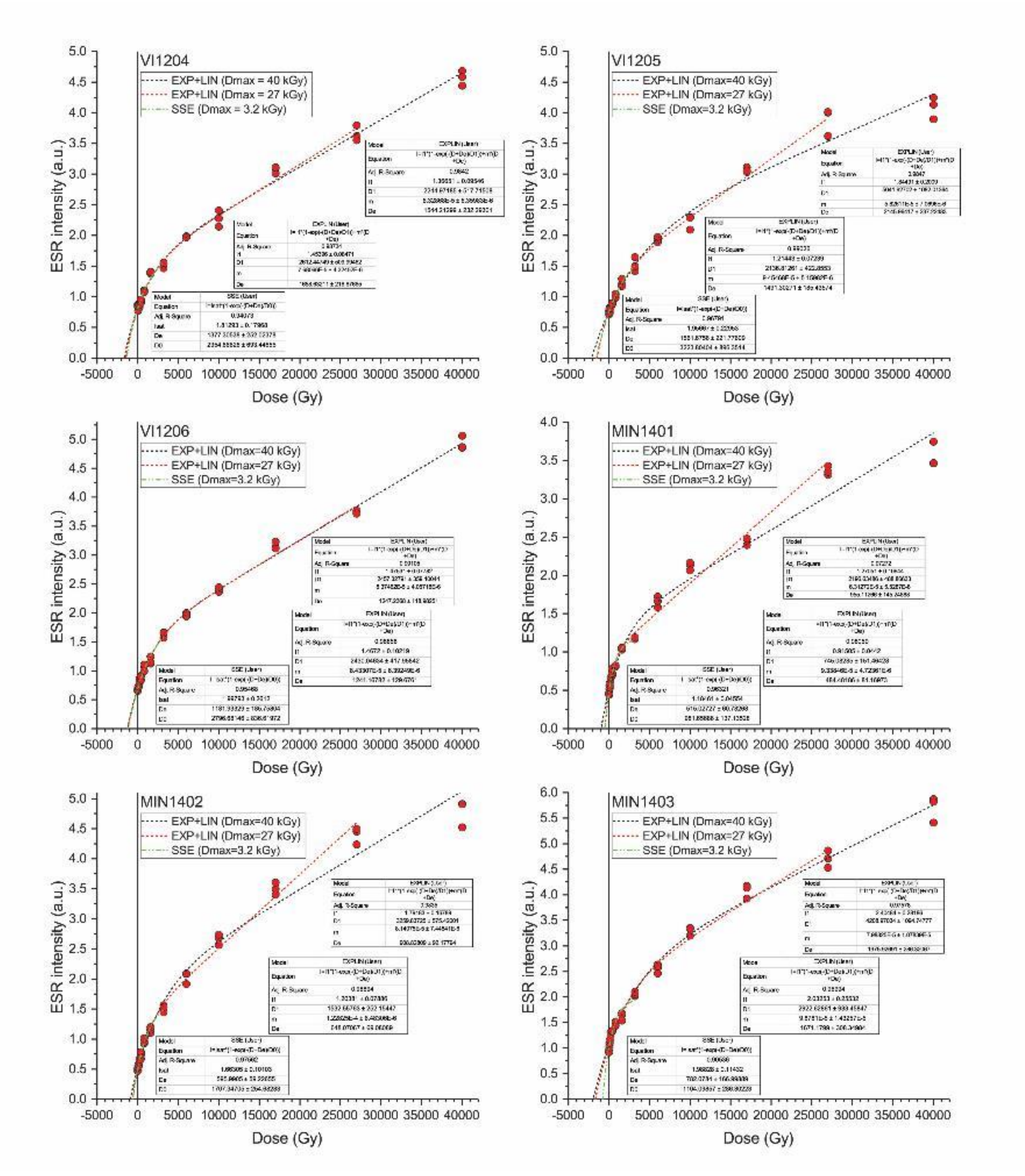

Fig. S 5. Dose Response curves derived from the ESR signal of the Al centre measured in the quartz samples from Porto Maior. Three different fittings were performed (see further details in the text): (i) EXP+LIN function with D_{max} = 40 kGy, (ii) EXP+LIN function with D_{max} = 27 kGy and (iii) SSE function with D_{max} = 3.2 kGy.

ESR DRCs obtained for some the Porto Maior samples do not show the standard linear behaviour at high irradiation dose and there may be an issue with the fitting of the most irradiated points with the linear term of the function (see VI1205, MIN1401 and MIN1402 in Fig. 5). To evaluate whether the maximum irradiation dose selected may have a significant impact on the calculated D_E, fittings were carried out using a standard EXP+LIN function with D_{max}=40 kGy and 27 kGy. For 2/6 samples, the D_E values remain within error, but in most cases D_E results derived from D_{max}=27 kGy are significantly lower than those from D_{max}=40 kGy, between -15 and -49% (Table S1). We consider the results derived from the former as being more reliable, for the following reasons:

- Goodness-of-fits for the six samples is on average better with D_{max}=27 kGy.
- We used a SSE function fitted through the first part of the DRCs (D_{max}=3.2 kGy) in order to evaluate the impact of high irradiation doses on the final D_E results. Despite the relatively low goodness-of-fit, fitting results provide some interesting indications. Leaving sample MIN1403 aside, resulting D_E values are consistent within 10% with those obtained for EXP+LIN and D_{max}=27 kGy. In contrast the relative difference may reach 46% with D_{max}=40 kGy for the same data set.

Consequently, ESR age calculations were performed using D_E values derived from the use of an EXP+LIN function with D_{max}=27 kGy and by pooling all the repeated ESR measurements of a given sample in a single DRC (Table S1), as recommended by [10]. With only one sample (MIN1403) showing an adjusted r^2 value <0.98, goodness-of-fit is overall good, suggesting thus reliable fitting results. We suspect the D_E value obtained for MIN1403 to be quite overestimated, given the significant difference observed between the EXP+LIN and SSE fittings, although it is unclear whether the SSE results is the correct one given the poor goodness-of-fit achieved.

1.1.2. Ti centres

Following the recommendations by [8], ESR intensities of the Ti-Li centre was assessed through options A and D. Both options actually provide D_E estimates within standard errors. Consequently, option D was finally used for the final dose evaluation. Fitting was carried out using a Ti-2 function with weighting by $1/s^2$ (s = experimental error). ESR DRCs and fitting results are displayed in Fig. S6 and Table S2, respectively.

Ti-Li centre					
Sample	Repeatability ESR intensities (%)	D_E repeatability (%)	Adj. r^2	Ti-2 (opt.D) D_E (Gy)	Reliability
VI1204	1.6%	14.7%	0.986	970±49	Good
VI1205	3.8%	10.1%	0.992	870±30	Excellent
VI1206	3.1%	9.8%	0.984	839±34	Good
MIN1401	2.5%	10.8%	0.980	432±28	Good
MIN1402	2.4%	4.6%	0.975	729±41	Regular
MIN1403	0.9%	4.7%	0.977	1034±66	Regular

Table. S 2. ESR data derived from the measurement of the Ti-Li centre. Following the criteria defined by [10], the reliability of the fitting results is assessed through the value of the adjusted r2: poor for r2<0.97, regular for 0.97<r2<0.98, good if 0.98<r2<0.99, excellent for r2>0.99.

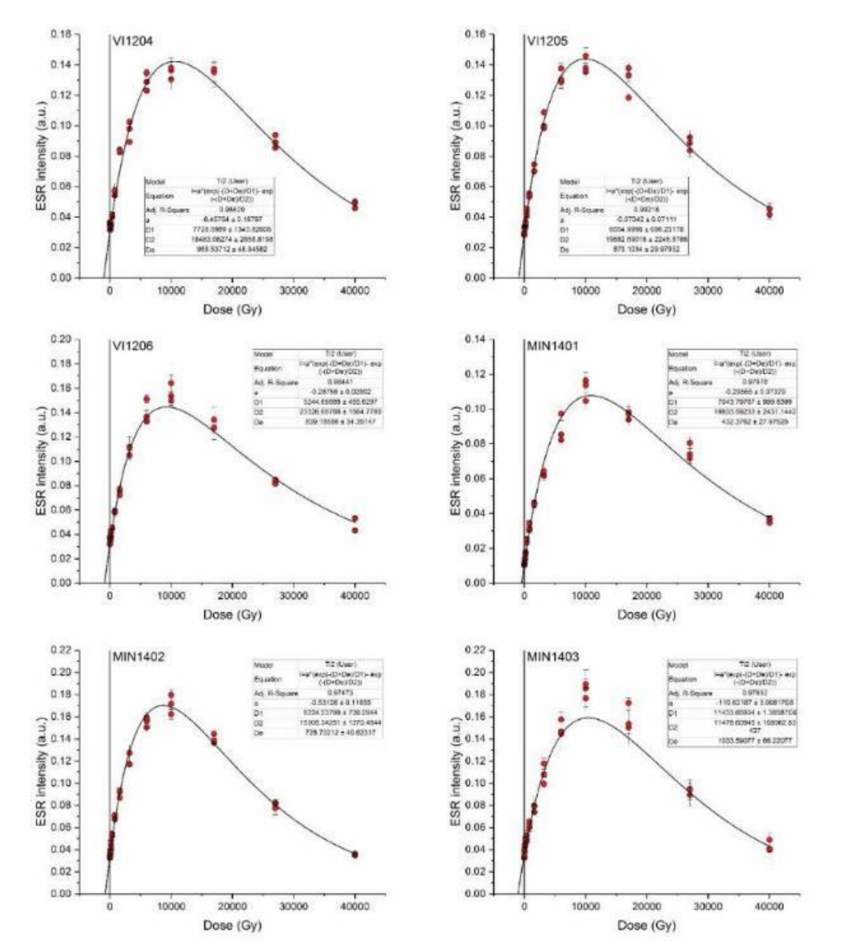

Fig. S 6. Dose Response curves derived from the ESR signal of the Ti-Li centre (option D) measured in the quartz samples from Porto Maior

The quality of the ESR data collected for the Ti-Li centre appears to be overall similar to those obtained for the Al centre (average intensity repeatability: 2.4 vs. 1.9 %; average D_E repeatability: 9.1 vs 11.9%). nIn terms of goodness-of-fit, all samples show a adjusted r^2 value > 0.97 ensuring meaningful fitting results. It should be mentioned here that the ESR DRC of VI1204 shows the peculiarity of an unexpectedly high ESR intensity for the natural point, i.e. higher than that of the points irradiated to $\leq$ 200 Gy. This has, however, a limited impact on the D_E result, as the fitting carried out without the natural point would only decrease the dose estimate by 5%.

Finally, it should also be mentioned here that the Ti-H centre was evaluated as well for this set of samples. Recent dating studies have shown the potential of this centre for late Middle Pleistocene chronologies [4]. However, the samples from Porto Maior exhibit an overall low ESR signal intensity for the Ti-H centre, about 0.20-0.30 the intensity of option A, i.e. in the range of the observations by [8], and much lower than the Cuesta de la Bajada samples [4]. As a consequence, measurement repeatability is far below the acceptable level, and fitting results are simply unreliable. To summarise, no meaningful value could be extracted for the Ti-H for any of the samples, with the exception of MIN1401. This sample shows the highest Ti-H signal intensity (33% of that of the option A). Although significantly worse than for other signals, the goodness-of-fit achieved for MIN1401 is nevertheless somewhat acceptable, as this is the only sample with an adjusted r^2 value >0.90. This results in a D_E estimate of 166$\pm$33 Gy (see Fig. S7).

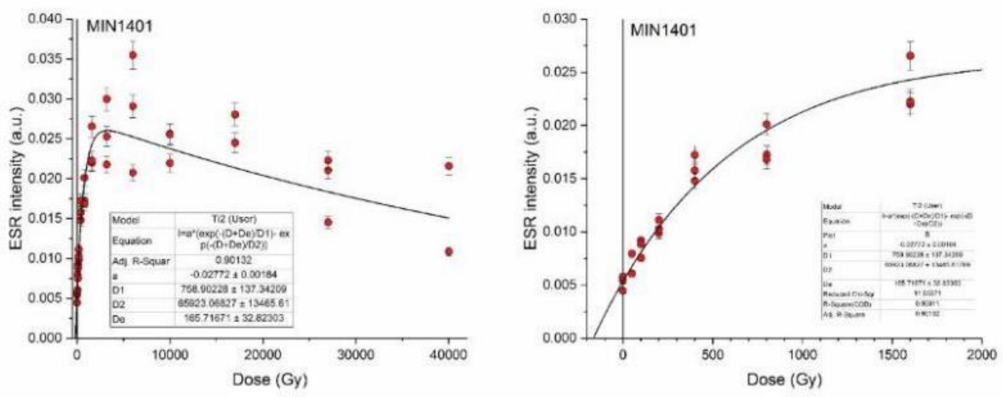

Fig. S 7. Dose Response curve derived from the measurement of ESR signal of the Ti-H centre measured in MIN1401.

1.1.3. <u>D_E comparison</u>

The comparison of the D_E values derived from the Al and Ti-Li centres shows that only 2 samples have consistent results (MIN1401 and MIN1402). The Al centre systematically

provides higher D_E values by 50 to 70% for the other samples, although it should be mentioned that in the case of MIN1403 the Al-D_E value is suspected to be overestimated. In accordance with the basic principles of the Multiple Centres approach, this suggests that for these samples the signal of the Al centre has been incompletely reset during sediment transportation. Consequently, the Ti-Li centre most likely provides the best estimation for the burial dose of the samples.

1.2. Dose rate evaluation

Sediment was analysed *in situ* as well as in the laboratory by a range of different techniques. ICP-MS and HRGS analyses overall provide highly consistent results (Table S5). Some of the differences observed may be due to some inherent variability within the sediment, as ICP and HRGS analyses were performed on ~10 g and ~150 g of raw sediment, respectively. HRGS analysis do not show any significant disequilibrium within the U-238 series decay chain, except perhaps for sample VI1204 with a slight Rn loss (15%). Final dose rate evaluations for ESR age calculations were carried out using the ICP results for the alpha and beta components. However, it should be mentioned here that the use of HRGS data would virtually make no difference, as the relative difference on the beta dose rates would not exceed 5%.

Sample	Unit	ICP-MS measurements			High Resolution Gamma Spectrometry (HRGS)			
		U-238 (Bq/kg)	Th-232 (Bq/kg)	K-40 (Bq/kg)	U-238 (Bq/kg)	Rn-222 (Bq/kg)	Th-232 (Bq/kg)	K-40 (Bq/kg)
VI1204	PM3	68.3±2.5	75.8±3.2	1.79±0.05	72.4±6.7	61.5±3.9	81.0±3.9	1.70±0.06
VI1205	PM3	75.3±3.2	77.9±3.3	1.77±0.05	74.8±6.7	73.7±4.7	76.3±4.4	1.69±0.06
VI1206	PM4	89.5±3.2	70.6±3.0	1.71±0.05	n.m.	n.m.	n.m.	n.m.
MIN1401	PM5	49.2+1.9	63.4+2.7	1.82+0.05	n.m.	n.m.	n.m.	n.m.
MIN1402	PM4	66.1+2.4	82.2+3.4	1.82+0.05	75.4±6.7	73.1±4.7	86.7±5.0	1.82±0.06
MIN1403	PM1	131.0+4.6	35.7+1.5	2.16+0.06	121.4±10.8	124.6±7.9	38.5±2.3	2.19±0.07

Table. S 3. Comparison of the radionuclide contents measured by ICP and HRGS. To facilitate comparison, concentrations obtained by ICP-MS measurements have been converted into activities values. Key: n.m.= not measured.

1.3. ESR age estimates

ESR age estimates were calculated using the D_E values derived from both the Al and Ti-Li centres, gamma dose rates measured *in situ* and beta dose rate derived from ICP-MS analyses (Table S4).

Sample	MIN1403	VI1204	VI1205	VI1206	MIN1402	MIN1401
Unit	PM1	PM3	PM3	PM4	PM4	PM5
Depth (m)	4±1	2.0±0.5	2.5±0.5	1.0±0.5	1.0±0.5	1.0±0.5
Assumed Water (AW) content (wet weight %)	20±5	20±5	20±5	20±5	20±5	13±5
Internal dose rate (µGy/a)	50±30	50±30	50±30	50±30	50±30	50±30
Alpha dose rate (µGy /a)	100±84	98±82	103±86	103±87	101±85	88±74
Beta dose rate (µGy/a)	2367±193	1855±149	1903±153	1946±156	1882±153	1855±139
Gamma dose rate (µGy/a)	1309±120	1344±123	1100±100	1272±116	1359±125	1322±115
Cosmic dose rate (µGy/a)	111±10	151±15	140±13	179±18	179±18	179±18
Total dose rate (µGy/a)	3937±312	3498±274	3296±258	3549±277	3571±281	3494±252
D_E (Gy) Al centre	1671±308	1544±232	1491±185	1241±130	648±69	484±81
D_E (Gy) Ti-Li centre	1034±66	970±49	870±30	839±34	729±41	432±28
**Age (ka) Al centre**	**424±85**	**441±75**	**452±66**	**350±46**	**181±25**	**138±25**
**Age (ka) Ti-Li centre**	**264±27**	**279±26**	**266±23**	**238±21**	**206±20**	**124±12**
**Age (ka) Ti-H centre**	**-**	**-**	**-**	**-**	**-**	**48±10**
Measured Water (MW) content (wet weight %)	9±5	13±5	10±5	10±5	11±5	9±5
Age (ka) Al centre	365±72	404±68	398±58	307±39	161±21	131±24
Age (ka) Ti-Li centre	227±22	255±23	233±19	208±18	182±12	118±11
Age (ka) Ti-H centre	-	-	-	-	-	45±9
Ti-Li age ratio AW/MW	1.06	1.09	1.14	1.14	1.13	1.06

Table. S 4. ESR age estimates and dose rate components. Errors are 1 sigma.

Given the slow bleaching rates of its ESR signal, the Al centre is usually considered to provide by default a maximum possible chronology for the deposits. In contrast, the chronology derived from the Ti-Li centre represents most likely the best age estimate for the sediment deposition.

Sample MIN1403 collected from the bottom of the local sequence provides an age estimate of 264±27 ka, which should be interpreted as a maximum age constraint for the archaeological deposits located a few meters above. The two ages obtained for unit PM3 (samples VI1204 and VI1205) are internally consistent and cannot be distinguished from that of PM1. This suggest a rapid sediment deposition. The 2 samples collected from unit PM4 yield slightly younger results, although consistent at 2 sigma. Finally, the sample collected at the top of the sequence within PM5 provides a significantly younger result of 124±12 ka. For this sample, the Al age is consistent around 140 ka, but the Ti-H centre suggests a much younger age around 50 ka. From a methodological point of view this age should however be considered with caution given the poor goodness-of-fit achieved for the D_E determination.

Usually, the water content (WC) is one of the main source of uncertainty in ESR dating, as it is extremely complicated to evaluate its variability over time. ESR age estimates based on the measured WC of the sediment are indicated in Table S4. Although they are between 6% and 14% lower than those based on the assumed water content values, they nevertheless remain within error. This shows the relatively moderate impact of this parameter on the final age estimate. However, because the ESR samples were collected during relatively dry periods (October 2012 and August 2014) and at shallow depths from the section surface (<30 cm), the moisture measured in the laboratory most likely underestimates the long term WC. This is why we assumed higher values (20% for all samples, and 13% for MIN140) with large errors (±5%) for age calculations. These values range from 10 to 30% at a two sigma confidence level, which most likely cover most of the long term WC variability. Additionally, they are consistent with the values used for luminescence dating. Consequently, we consider the ESR age results based on assumed WC as the best estimates to constrain the chronology of the deposits at Porto Maior.

SI Luminescence dating

In total, four samples were collected for luminescence dating at the site of Porto Maior (Fig. 1). Two samples, PM16-3 and PM16-5, were collected from fluvial sediment layers located immediately below the excavated archaeological horizon, within lithostratigraphic unit PM3. The lowermost sample, PM16-3, was collected ~1.5 m below the archaeological layer and near the base of a massive to weakly bedded sandy deposit. Sample PM16-3 was also located ~20 cm above the contact with the underlying deposit (PM2), which is made up predominantly of gravels and cobbles supported by sand and silts. Sample PM16-5 was collected 105 cm above sample PM16-3 in the upper part of unit PM3, and was situated immediately below the archaeological horizon in level PM4. Two samples, PM16-4 and PM16-6, were collected from deposits overlying the excavated artefacts. Sample PM16-6 was taken ~30 cm below the upper contact of a red/orange silty clay fluvial deposit (PM4) and was located 50 cm above the archaeological layer. Sample PM16-4 was collected from the overlying unit (PM5), which is a 125 cm-thick deposit that sits unconformably on top of the fluvial sequence. The upper sections of this unit is of aeolian origin; it is composed of well-sorted and weakly bedded sandy silt and displays a different lithology and texture to the underlying fluvial deposits. Sample PM16-4 was collected 70 cm above the lower contact of unit PM5 and 10-15 cm above ESR sample MIN1401. No luminescence dating samples were collected from levels PM1 and PM2.

Sample preparation

Samples were collected by inserting 20 cm-long, opaque PVC tubes into cleaned vertical exposures. Sample tubes were immediately sealed with duct tape and wrapped in black plastic bags for safe storage and transportation. The 90-125 µm K-feldspar fraction of these samples was extracted under subdued red light conditions following standard procedures [20]. Sediment samples were initially sieved to isolate the fine sand fraction (90-300 µm), and treated with H_2O_2 and HCl to eliminate organics and carbonates, respectively. Heavy liquid separation was used to isolate K-feldspar grains in the 2.53 to 2.58 g/cm^3 density range. The 90-125 µm fraction was then sieved and etched with 10% hydrofluoric acid for 10 minutes to remove the outer 10 µm rind of each grain. The etched grains were then washed in 30% hydrochloric acid to remove any precipitated fluorides and re-sieved using a 63 µm sieve to eliminate any disaggregated grains. The 212-250 µm quartz fraction of sample PM16-4 were prepared following the same procedure, with the exception that heavy liquid densities of 2.62 g/cm^3 and 2.72 g/cm^3 were used for

separating feldspars and heavy minerals, respectively. The selected quartz grain fraction was etched with 48% hydrofluoric acid for 40 minutes. As with K-feldspars, samples were re-sieved with a 63 µm sieve after the etching procedure.

Environmental dose rate estimation

Dose rate assessments were made using a combination of in situ gamma spectrometry measurements and low-level beta counting (Table S5). Field gamma spectrometry measurements were performed at each luminescence dating sample position immediately after removal of the PVC tubes. Elemental concentrations of K, U and Th were determined from the field gamma spectra using the 'energy windows' method described in [21]. Additional sediment samples were collected from the area around each luminescence dating sample position for beta dose rate assessments (beta counting), water content evaluations, and high-resolution gamma-ray spectrometry (HRGS) measurements. Low-level beta counting was performed on dry and homogenised sediment using a Risø GM-25-2 beta counter [22]. HRGS measurements were used to investigate the state of secular equilibrium in the ^{238}U and ^{232}Th decay series. Daughter-parent isotopic ratios for ^{238}U, ^{226}Ra, ^{210}Pb, ^{228}Ra and ^{228}Th are consistent with unity at either 1σ or 2σ, indicating that the ^{238}U and ^{232}Th chains exhibit present-day secular equilibrium (Table S6). Cosmic-ray dose rates were calculated using the approach described in [18]. Internal dose rate contributions for K-feldspar grains have been estimated using an assumed internal ^{40}K content of 12.5 ± 0.5% [23] and ^{87}Rb content of 400 ± 100 ppm [24].

The beta, gamma and cosmic-ray dose rates have been corrected for estimated long-term water contents of each sample [25,26]. The present-day sediment water contents ranged between 10 and 19% of dry sediment weight (Table S13) but they are not considered to be entirely representative of long-term moisture conditions at the site because the sediment profiles had been exposed for 3 years in the excavation area (samples PM16-6 and PM16-4), and for more than 50 years in the abandoned railway trench (samples PM16-3 and PM16-5); and thus they had partially dried out prior to sampling. To determine more suitable long-term sediment moisture contents, we have adopted conservative estimates based on 50% present-day saturated water contents for each luminescence sample. A 1σ relative uncertainty of 20% has been assigned to the long-term moisture estimates to accommodate any potential variations in hydrologic conditions during burial (e.g., changes in effective sediment moisture between glacial and interglacial cycles). This approach yielded long-term sediment moisture contents of

20-29% for samples PM16-3, PM16-5 and PM16-6 (Table S5), which overlap with published values used in reliable, known-age luminescence dating studies of Middle Pleistocene fluvial and alluvial deposits from Spain (e.g., [27-30]). For sample PM16-4, which comes from the uppermost aeolian unit (PM5), the water content value used to calculate the final ages is based on values adopted in other luminescence dating studies of wind-blown (loess) sequences from Europe. These values range from 10% to 20%, and so we applied a value of 15 ± 5% for samples PM16-4 [31-37]. Table S13 compares the final pIR-IR ages obtained using our preferred estimates of the long-term water contents (which account for recent loss of moisture due to profile exposure) and the ages that would be obtained using the present-day (as measured) water content values of each sample. It can be observed that the corresponding ages for each sample are not statistically different at 2σ, and therefore we conclude that our age estimates and interpretations are not sensitive to our preferred choice of long-term water content. The adopted long-term moisture estimates, which are expressed as percentages of dry sediment weight, are also consistent with the long-term water contents for the ESR dating samples (20 ± 5%), which are expressed as percentages of wet sediment weight.

Instrumentation and equivalent dose (D_e) measurements and estimation

Post-infrared infrared stimulated luminescence (pIR-IR) measurements were carried out using a Risø TL/OSL-DA-20 reader equipped with a calibrated $^{90}Sr/^{90}Y$ β radiation source delivering a dose rate of ~0.106 Gy/s [38]. pIR-IR signals were stimulated using IR diodes (875 nm, maximum power of 166 mW/cm^2) at 90% power and measurements were performed on 90-125 μm K-feldspar grains mounted on 9.7 mm-diameter stainless steel discs; approximately 160 grains were placed on each disc. Blue emissions were detected using an EMI 9235QB photomultiplier fitted with a 4 mm-thick Schott BG39, 3 mm-thick Corning 7-59, and 4 mm-thick Schott GG400 filter pack. Multi-grain OSL measurements on quartz (i.e., dose recovery test measurements) were stimulated using blue LEDs (470 nm, maximum power 52 mW/cm^2) at 90% power. Single-grain OSL signals were stimulated with a 10 mW Nd:YVO4 single grain laser attachment emitting at 532 nm (maximum power of ~50 W cm^2). Ultraviolet emissions were measured through a 7.5 mm-thick U340 filter. Quartz grains with a diameter of 212-250 μm were measured in aluminium discs drilled with an array of 300 × 300 μm holes to ensure true single-grain resolution [28].

Equivalent dose (D_e) measurements were made on 160-grain K-feldspar aliquots using modified versions of the pIR-IR single-aliquot regenerative dose (SAR) protocols detailed by [39] and [40] (Table S7). As part of these protocols, pIR-IR stimulations are either

performed at 225 ^oC following a preheat of 250 ^oC for 60 s (pIR-IR_{225} signals) or at 290 ^oC following a preheat of 320 ^oC for 60 s (pIR-IR_{290} signals). pIR-IR signals are measured for the naturally accumulated dose (L_n) of each aliquot, as well as for a series of different sized laboratory doses (L_x). Each of the natural and regenerative dose pIR-IR measurements are subsequently normalised for sensitivity change using a fixed test dose pIR-IR measurement (T_x), and the sensitivity-corrected natural (L_n/T_n) is then interpolated onto the sensitivity-corrected L_x/T_x dose-response curve to obtain a D_e value. Following [41], pIR-IR signal measurements were made for 200 s and the IR diodes were switched on 10 s after reaching the desired measurement temperature to minimise unwanted isothermal TL contributions to the pIR-IR signal [42]. A high temperature IR wash was also added at the end of each SAR measurement cycle to minimise the effect of charge transfer on the sensitivity-corrected pIR-IR signal responses.

Multi-grain K-feldspar D_e values were calculated from the first 10 s of stimulation after subtracting a mean background count from the last 20 s of stimulation. Individual D_e values were included in the final age calculation if they passed the following quality assurance criteria: (i) the recycling ratio (i.e., sensitivity-corrected luminescence responses (L_x/T_x) for two identical regenerative doses) was consistent with unity at 2σ; (ii) the recuperation ratio, calculated as the ratio of the sensitivity-corrected 0 Gy dose point (L_0/T_x) to the sensitivity-corrected natural (L_n/T_n), was <5%; (iii) the sensitivity-corrected natural signal intercepted the sensitivity-corrected dose-response curve or it did not intercept the saturated part of the dose-response curve (i.e., the L_n/T_n value was equal to, or lower than, the I_{max} saturation limit of the dose-response curve at 2σ); (iv) the dose-response curve did not display anomalous properties (e.g., zero or negative responses with increasing dose) and resulted in good Monte Carlo fits.

Single-grain OSL D_e values were measured using the SAR protocol shown in Table S7. The most suitable preheat combination was determined through a dose recovery plateau test (see below). The regenerative and test dose OSL signals were measured for 2 s at 125 °C. D_e values were calculated by integrating the first 0.08 s of stimulation and subtracting a late-light background from the last 0.25 s. The applied single-grain OSL protocol follows the same principles as described for the SAR pIR-IR measurements. In addition to the quality assurance criteria outlined above for the pIR-IR signal, single-grain OSL D_e values were accepted when: (i) the luminescence signals were statistically distinguishable from background (the net intensity of the natural test dose signal, T_n, was >3σ above the late-light background signal); (ii) the net T_n signal had a relative error of <30%; (iii) the high-dose recycling ratio (i.e., sensitivity-corrected luminescence responses (L_x/T_x) for two identical high regenerative doses) was consistent with unity at

2σ; (iv) no contamination by feldspar grains or inclusions was detected using the OSL IR depletion ratio [43] (i.e., the ratio of the L_x/T_x values obtained for two identical regenerative doses measured with and without prior IR stimulation overlapped with unity at 2σ). Table S9 shows the proportion of accepted and rejected grains obtained after single-grain OSL measurements of sample PM16-4.

Individual D_e estimates are presented with their 1 standard error ranges (Table S10-S11), which have been derived from three sources of uncertainty: (i) a random uncertainty term arising from photon-counting statistics for each OSL measurement, calculated using equation 3 of [44]; (ii) an empirically determined instrument-reproducibility uncertainty of 0.5% for each multi-grain aliquot measurement and 1.9% for each single-grain measurement, calculated specifically for the reader used in this study; and (iii) a dose-response curve fitting uncertainty determined using 1,000 iterations of the Monte Carlo method implemented in Analyst [45].

pIR-IR dose recovery tests and signal characteristics

To determine the most suitable pIR-IR measurement and preheat conditions for the Porto Maior samples, we undertook dose recovery tests using the $pIR\text{-}IR_{225}$ and $pIR\text{-}IR_{290}$ SAR protocols shown in Table S7. Ten 160-grain K-feldspar aliquots of sample PM16-6 were prepared and placed under direct sunlight for 8 hrs to bleach their naturally accumulated pIR-IR signals. These bleached aliquots were then split into two batches (*n*=5 each) for the $pIR\text{-}IR_{225}$ and $pIR\text{-}IR_{290}$ dose recovery tests. For each batch of aliquots, two were left un-dosed to determine the residual (unbleached) D_e remaining after daylight bleaching, while the remaining three were given a laboratory dose of 700 Gy. The two batches of five aliquots were then separately measured with the $pIR\text{-}IR_{225}$ or $pIR\text{-}IR_{290}$ SAR protocol to determine their D_e values. Dose recovery (measured to given dose) ratios were calculated after subtracting the residual (unbleached) D_e of the un-dosed aliquots from the mean D_e obtained from the dosed aliquots (Table S8).

The $pIR\text{-}IR_{225}$ dose recovery ratio of sample PM16-6 overlaps with unity at 2σ (0.97 ± 0.02) and supports the suitability of this protocol for D_e determination. In contrast, the $pIR\text{-}IR_{290}$ signal yielded an inaccurate dose-recovery ratio at 2σ and overestimated the administered dose by ~29% (1.29 ± 0.04). These dose-recovery test results are consistent with trends observed in other pIR-IR studies of Middle Pleistocene deposits from northern Spain [30,41,46,47]. On the basis of these dose-recovery assessments, we have chosen to employ the $pIR\text{-}IR_{225}$ protocol for dating purposes at Porto Maior. The

pIR-IR$_{290}$ signal is not considered further for these samples as it does not satisfy the minimum dose-recovery requirements for SAR reliability.

OSL dose recovery tests were additionally undertaken on sample PM16-4 to determine the most suitable preheat combination for quartz single-grain OSL D_e measurements. Initial dose recovery tests were performed on 1500-grain aliquots of sample PM16-4 using a modified version of the SAR protocol shown in Table S7 (replacing 125°C green laser stimulations with 125°C blue LED stimulations for 60 s, and inserting a 50°C IR bleach for 40 s prior to each OSL measurement to remove any feldspar signal contamination). Dose recovery D_e measurements were made after bleaching the naturally-accumulated signal of the multi-grain aliquots with blue LEDs (two 50°C blue LED exposures of 1000 s, separated by a pause of 10,000 s), and administering a known laboratory dose of 50 Gy dose. A test-dose preheat of 160°C for 10 s was used for all measurements. Four regenerative-dose preheats were tested on batches of four aliquots (200°C for 10 s, 220°C for 10 s, 240°C for 10 s, 260°C for 10 s). The most suitable preheat combination for this sample involved a regenerative-dose preheat of 220°C for 10 s and a test-dose preheat of 160°C for 10 s (dose recovery ratio = 1.00 ± 0.02; recycling ratio = 1.00 ± 0.01) (Fig. S8). To ensure this preheat combination was equally suitable for single-grain OSL D_e determination, we repeated the dose recovery test on individual quartz grains using the SAR sequence shown in Table S7. The D_e values of 1000 grains were measured after their natural signals had been bleached using blue LEDs and a dose of 62.5 Gy had been administered. The mean recovered to given dose ratio obtained was 0.97 ± 0.02 and the overdispersion was 0%, confirming the suitability of the chosen preheat combination for this sample (Table S9; Fig. S8).

A representative pIR-IR$_{225}$ decay curve and sensitivity-corrected dose-response curve is shown in Fig. S9a-b**.** The pIR-IR$_{225}$ decay curves of these samples typically decrease by ~90% within the first 30 s of stimulation and are optimally fitted with a single saturating exponential plus linear function. All the D_e values were obtained from the region of the dose-response that was not in saturation when using this type of fitting function. All of the measured aliquots passed the SAR quality assurance criteria outlined in the previous section. Fig. S9c-d shows the OSL dose-response and decay curves obtained for a grain of sample PM16-4. The example shown represents a typical grain accepted after applying the SAR quality assurance criteria. The OSL signal is fast-decaying and reaches background within 0.6 s of stimulation, while the dose response curve is best fitted with an exponential function.

Residual dose assessments

In order to assess the bleaching properties of the pIR-IR_{225} signal for these samples, and to examine whether appropriate levels of signal resetting could have been experienced prior to burial, we performed a series of prolonged and controlled daylight exposure tests on samples PM16-4 and PM16-6. For this experiment, three K-feldspar aliquots of each sample were prepared and placed under direct sunlight for 15 days. The residual D_e values of these bleached aliquots were then measured using the pIR-IR_{225} SAR protocol shown in Table S7.

The mean pIR-IR_{225} residual doses for samples PM16-4 and PM16-6 are 0.90 ± 0.03 Gy and 3.57 ± 0.36 Gy, respectively (Table S10). These results provide good support for the potential bleachability of the pIR-IR_{225} signal for the fluvial and aeolian deposits being dated in this study. The measured residual doses of these two samples equate to 1.3% and 0.4% of their natural D_e values, and lie well within the existing 1σ uncertainties of the final age estimates. Given the inconsequential size of these empirical residual D_e values, and the unknown bleaching durations experienced by each of the dating samples prior to deposition, we have not considered an additional residual dose subtraction in the final $pIRIR_{225}$ age estimates.

Fading rates

To investigate the potential for athermal loss of K-feldspar pIR-IR_{225} signals over burial timescales we performed anomalous fading assessments on subsets of aliquots used to derive D_e values. For this purpose, three 160-grain aliquots of each sample were measured following the procedures of [48], which involved undertaking repeated SAR L_x/T_x measurements after different storage times of 0.17 – 30 h. Anomalous fading rates (*g*-value) normalised to 2 days were calculated as described in [49], and used to quantify the expected percentage of signal loss per decade of storage time.

The weighted-mean *g*-values for the Porto Maior samples range between 0.86 ± 0.23 %/decade and 1.22 ± 0.42 %/decade (Table S11), and the combined weighted average g-values for all individual aliquots (*n*=12) is 0.89 ± 0.09%/decade. These empirical fading rates are all within 2σ of 1%/decade, and are similar to those published previously for higher temperature pIR-IR_{290} signals (see summary in [41]) and athermally stable quartz OSL signals [50]. They are also consistent with pIR-IR_{225} *g*-values reported for a range of known-age Middle Pleistocene samples from northern Spain [30,41,46,47,51]. Such low g-values (on the order of <1-2%/decade) have been interpreted to be potential unreliable indicators of long-term fading rates and / or artefacts of laboratory procedures on the

basis of comparisons made with independent age control, observations of natural signal saturation, and measurements of similarly sized *g*-values for quartz [50,52,53]. Consequently, we do not consider the low g-values recorded here to be indicative of the need for pIR-IR age corrections. The consistency of the existing pIR-IR_{225} ages and the replicate single-grain OSL and quartz ESR ages at Porto Maior (Fig. 1) similarly does not support the need for additional fading corrections in this context.

D_e results and ages

Table S11 summarises the D_e values, overdispersion and pIR-IR_{225} ages obtained for the Porto Maior samples. The D_e distributions of each sample have been plotted as radial plots in Fig. S10. All samples have a moderate to low overdispersion value (i.e., the degree of scatter beyond the empirical D_e uncertainties) ranging between 5 and 22%, and are generally indicative of single dose populations (Table S11, Fig. S10). As such, the central age model (CAM; [54]) was used to derive the final pIR-IR D_e values for these samples.

The single-grain OSL D_e distribution of sample PM16-4 shows a moderate amount of scatter, but the overdispersion (29 ± 5%) is consistent at 2σ with values of ~20% commonly reported for well-bleached and undisturbed samples. The D_e distribution is not significantly skewed according to the weighted skewness test outlined by [55] and [56] (Table S12; Fig. S11). We interpret these D_e characteristics as indicative of sufficient bleaching prior to deposition, and have therefore used the CAM to derive the final single-grain D_e value for PM16-4 (60.8 ± 3.1 Gy).

The CAM D_e values for the lowermost three samples from units PM3-PM4 are much higher (850-1000 Gy) than for the uppermost sample collected from unit PM5 (<100 Gy). This trend is consistent with the presence of a distinct erosional contact between the fluvial deposits and capping aeolian unit. The pIR-IR_{225} ages for the two samples from unit PM3 are stratigraphically indistinguishable (PM16-3 = 259 ± 29 ka; PM16-5 = 268 ± 24 ka). These two ages are slightly older than, but within 2σ of, sample PM16-6 (231 ± 15 ka), which was collected from the overlying stratigraphic unit (PM4). Collectively, these ages indicate that the fluvial deposits found directly above and below the archaeological layer at Porto Maior were deposited during a time period likely encompassing the latter part of MIS 8 and beginning of MIS 7. The uppermost sample collected from unit PM5 produced pIR-IR_{225} and single-grain OSL ages of 17 ± 1 ka for both signals, which indicate that the wind-blown deposits overlaying the fluvial sediments at Porto Maior were deposited during MIS 2.

Sample	Sample depth (m)	Water content [a]	Mineral	Grain fraction (µm)	Environmental dose rate (Gy / ka) [b] Gamma dose rate [c]	Beta dose rate [d]	Cosmic dose rate [e]	Internal dose rate (U+Th) [f]	Internal dose rate (K + Rb) [g]	Total dose rate [h]
PM16-4	0.60	15.0	K-feldspar	90-125	1.49±0.05	1.99±0.10	0.17±0.02	0.06±0.03	0.43±0.03	4.14±0.19
PM16-4	0.60	15.0	Quartz	212-250	1.49±0.05	1.86±0.09	0.17±0.02	0.03±0.01		3.55±0.18
PM16-6	0.55	24.9	K-feldspar	90-125	1.28±0.05	1.75±0.09	0.15±0.02	0.06±0.03	0.43±0.03	3.69±0.20
PM16-5	1.55	19.8	K-feldspar	90-125	1.38±0.05	1.70±0.08	0.14±0.02	0.06±0.03	0.43±0.03	3.71±0.19
PM16-3	3.00	28.5	K-feldspar	90-125	1.39±0.05	1.80±0.09	0.11±0.01	0.06±0.03	0.43±0.03	3.79±0.22

[a] Water content used for calculating environmental dose rates, expressed as % of dry mass of sample and assigned a relative uncertainty of ± 20%. For samples PM16-3, PM16-5 and PM16-6 the long-term water contents are 50% of saturated values. For sample PM16-4 the long -term water content value was set to 15 ±5%.

[b] Radionuclide concentrations and specific activities have been converted to dose rates using the conversion factors given in [57] and [58], making allowance for beta-dose attenuation [15,59].

[c] Gamma dose rates were calculated from *in situ* measurements made at each sample position with a NaI:Tl detector using the 'energy windows' method detailed in [21].

[d] Beta dose rates were calculated using a Risø GM-25-5 low-level beta counter [22], after making allowance for beta dose attenuation due to grain-size effects and HF etching [15].

[e] Cosmic-ray dose rates were calculated according to [18] and assigned a relative uncertainty of ± 10%.

[f] Assumed internal (alpha plus beta) dose rate for K-feldspar grains, the Internal alpha and beta dose rate contributions from ^{238}U and ^{232}Th were calculated using assumed concentrations of 0.15 ± 0.03 ppm and 0.35 ± 0.07 ppm, respectively, based on modal values obtained by [60] and similar values obtained by [61-63]. An a-value of 0.09 ± 0.03 was used to estimate the internal alpha dose rate contributions from these ^{238}U and ^{232}Th concentrations based on published estimates obtained for a wide range of k-feldspar samples -e.g. [27,64-68] -. The assumed internal (alpha plus beta) dose rate for the quartz fractions is based on published ^{238}U and ^{232}Th measurements for etched quartz grains from a range of locations [60,69-71] and an alpha efficiency factor (a-value) of 0.04 ± 0.01 [64,72].

[g] Internal dose rate of feldspar grains arising from ^{40}K and ^{87}Rb concentrations were calculated from assumed values of 12.5 ± 0.5% [23] and 400 ± 100 ppm [24], respectively.

[h] Mean ± total uncertainty (68% confidence interval), calculated as the quadratic sum of the random and systematic uncertainties.

Table. S 5. Environmental dose rates obtained for the 90-125 µm K-feldspar grains at Porto Maior, Spain. Also shown is the dose rate for the 212-250 µm quartz grains of sample PM16-4.

Sample	Radionuclide specific activities (Bq/kg) [a, b]						Daughter: parent isotopic ratio		
	^{238}U	^{226}Ra	^{210}Pb	^{228}Ra	^{228}Th	^{40}K	^{226}Ra:^{238}U	^{210}Pb:^{226}Ra	^{228}Th:^{228}Ra
PM16-4	64.1 ± 7.9	59.2 ± 4.0	56.9 ± 6.5	67.2 ± 5.8	70.5 ± 5.7	571 ± 19	0.93 ± 0.13	0.96 ± 0.13	1.05 ± 0.12
PM16-6	94.4 ± 12.0	85.2 ± 5.8	97.3 ± 9.1	78.0 ± 7.0	69.2 ± 5.2	350 ± 19	0.90 ± 0.13	0.93 ± 0.12	0.89 ± 0.11
PM16-5	72.1 ± 9.2	61.8 ± 4.2	56.9 ± 6.5	73.8 ± 6.6	73.9 ± 5.7	487 ± 17	0.86 ± 0.12	0.92 ± 0.12	1.00 ± 0.12
PM16-3	86.6 ± 11.0	85.5 ± 5.8	85.1 ± 9.7	74.9 ± 6.8	77.0 ± 6.3	571 ± 21	0.99 ± 0.14	1.00 ± 0.13	1.03 ± 0.13

[a] Measurements made on dried and powdered sediment sub-samples of ~130 g. The specific activities of ^{238}U (determined from ^{235}U emissions after correcting for ^{226}Ra interference, and ^{234}Th emissions after correcting for ^{228}Ra interference), ^{226}Ra (derived from ^{214}Pb and ^{214}Bi emissions), ^{210}Pb, ^{228}Ra (derived from ^{228}Ac emissions), ^{228}Th (derived from ^{212}Pb, ^{212}Bi and ^{208}Tl emissions) and ^{40}K were measured for each sediment sample, and used to derive the daughter-to-parent isotope ratios for ^{226}Ra:^{238}U, ^{210}Pb:^{226}Ra and ^{228}Th:^{228}Ra shown in columns 8-10.

[b] Mean ± total uncertainty (68% confidence interval), calculated as the quadratic sum of the random and systematic uncertainties.

Table. S 6. Results obtained from high resolution gamma-ray spectrometry (HRGS) measurements of the ^{238}U and ^{232}Th decay chains performed on luminescence dating samples from Porto Maior (Spain). Values shown are the specific radionuclide activities (Bq kg^{-1}) and daughter-to-parent ratios.

Step	SAR pIR-IR_{225}	SAR pIR-IR_{290}	Step	SAR single-grain OSL	Signal
1	Dose (natural or laboratory)	Dose (natural or laboratory)	1	Dose (natural or laboratory)	
2	Preheat 1 (250ºC for 60 s)	Preheat 1 (320ºC for 60 s)	2[a]	IR stimulation (50ºC for 60 s)	
3	IR stimulation (50ºC for 200 s)	IR stimulation (50ºC for 200 s)	3	Preheat 1 (240ºC for 10 s)	
4	pIR-IR stimulation (225ºC for 200 s)	pIR-IR stimulation (290ºC for 200 s)	4	Single-grain OSL stimulation (green laser; 125ºC for 2 s)	L_x or L_n
5	Test dose (100 Gy)	Test dose (100 Gy)	5	Test dose (11 Gy)	
6	Preheat 2 (250ºC for 60 s)	Preheat 2 (320ºC for 60 s)			
7	IR stimulation (50ºC for 200 s)	IR stimulation (50ºC for 200 s)	6	Preheat 2 (160ºC for 10 s)	
8	pIR-IR stimulation (225ºC for 200 s)	pIR-IR stimulation (290ºC for 200 s)	7	Single-grain OSL stimulation (green laser; 125ºC for 2 s)	T_n or T_x
9	High temperature IR wash (at 290ºC for 100 s)	High temperature IR wash (at 325ºC for 100 s)			
10	Repeat measurement cycle for different sized regenerative doses	Repeat measurement cycle for different sized regenerative doses	8	Repeat measurement cycle for different sized regenerative doses	

[a] Step 2 is only included in the single-grain OSL SAR procedure when measuring the OSL IR depletion ratio (37).

Table. S 7. Single-aliquot regenerative-dose (SAR) protocol used in this study to measure the pIR-IR_{225} and pIR-IR_{290} dose recovery test and D_e values on multi-grain aliquots of K-feldspars. Also shown is the SAR sequence used for single-grain OSL measurements made on sample PM16-4.

Sample	Mineral/ Signal	Grains per disc	Grain size (µm)	Given dose (Gy)	Bleaching	Residual (non-dosed) assessment				Dose recovery test				
						accepted/ measured	Recycling ratio	Weighted mean D_e (Gy)	Over-dispersion (%)	accepted/ measured	Recycling ratio	Weighted mean D_e (Gy)	Over-dispersion (%)	Net measured/ given dose ratio
PM16-6	K-feldspar / pIR-IR_{225}	~360	90-125	700 ± 14	8 hours in daylight	2 / 2	0.99 ± 0.01	14 ± 2	16 ± 8	3 / 3	0.99 ± 0.01	691 ± 5	0 ± 0	0.97 ± 0.02
PM16-6	K-feldspar / pIR-IR_{290}	~360	90-125	700 ± 14	8 hours in daylight	2 / 2	0.98 ± 0.02	31 ± 1	1 ± 1	3 / 3	0.98 ± 0.01	934 ± 20	0 ± 0	1.29 ± 0.04

Table. S 8. Dose recovery test results for the pIR-IR_{225} and pIR-IR_{290} signals of sample PM16-6.

	Dose recovery test Single-grain OSL		Natural D_e Single-grain OSL	
**Sample name**	**PM16-4**		**PM16-4**	
Total number of grains measured	1000		1100	
	n	%	n	%
T_n <3σ background (non-luminescent grains)	614	61	695	63
Poor low-dose recycling ratio	64	6	64	6
Poor high-dose recycling ratio	36	4	45	4
Poor OSL IR depletion ratio	70	7	52	5
Recuperation (0 Gy L_x/T_x >5% of L_n/T_n)	0	0	0	0
Relative error on the net T_n signal >30%	107	11	129	12
Anomalous dose-response / unable to perform Monte Carlo fit [a]	25	3	30	2
Non-intercepting grains (L_n/T_n > dose-response curve saturation)	0	0	4	<1
Saturated grains (L_n/T_n ≈ dose-response curve saturation)	30	3	27	2
**Accepted grains (used for D_e determination)**	**54**	**5**	**54**	**5**

[a] Includes grains with zero or negative changes in L_x/T_x with increasing dose, and grains displaying very scattered L_x/T_x values that could not be successfully fitted with the Monte Carlo procedure.

Table. S 9. Single-grain OSL quality assurance statistics obtained for dose recovery and natural D_e measurements of sample PM16-4.

Sample	Mineral / Signal	Grains per disc	Grain size (µm)	Bleaching	Residual assessment			
					accepted/ measured	Recycling ratio	W-mean D_e (Gy)	Overdispersion (%)
PM16-4	K-feldspar / pIR-IR_{225}	~360	90-125	15 days in daylight	3 / 3	1.01 ± 0.01	0.90 ± 0.03	3 ± 4
PM16-6	K-feldspar / pIR-IR_{225}	~360	90-125	15 days in daylight	3 / 3	1.01 ± 0.01	3.57 ± 0.36	20 ± 9

Table. S 10. Residual equivalent dose values (D_e) for the pIR-IR_{225} signal of samples exposed to prolonged sunlight.

Sample	Accepted/ measured	Over-dispersion (%)	CAM D_e (Gy) [a]	Total dose rate (Gy / ka)	*g*-values (% / decade) [b]	Weighted skewness value [c]	Critical skewness 68% C.I. [c]	Critical skewness 95% C.I. [c]	Age (ka) [d]
PM16-4	6 / 6	5 ± 2	68.5 ± 1.5	4.14 ± 0.19	0.86 ± 0.23	0.14	1	2	17 ± 1
PM16-6	6 / 6	6 ± 2	851 ± 23	3.69 ± 0.20	0.95 ± 0.23	-0.01	1	2	231 ± 15
PM16-5	7 / 7	18 ± 5	994 ± 71	3.71 ± 0.19	1.01 ± 0.31	-0.22	0.93	1.85	268 ± 24
PM16-3	7 / 7	22 ± 7	981 ± 90	3.79 ± 0.22	1.22 ± 0.42	0.06	0.93	1.85	259 ± 29

[a] CAM = central age model of Galbraith [54].
[b] Laboratory fading rates were measured following the procedure suggested by [48]. The *g*-values were determined from repeated L_x/T_x measurements made after different storage times (ranging from 0.17 h to 30 h) using Eq. 4 of [49], and have been normalised to a measurement delay time of two days to enable direct comparisons with published values.
[c] Weighted skewness scores have been calculated on log-transformed D_e values using Eq. 7-8 [56]. Critical skewness scores have been calculated using Eq. 16 [55]. Critical skewness values are taken to be equivalent to the standard error of skewness score (68% C.I.) for multi-grain aliquot D_e datasets and twice the standard error of skewness score (95% C.I.) for single-grain D_e datasets, following the results of sensitivity analyses performed by [55] and [73].
[d] Mean ± total uncertainty (68% confidence interval), calculated as the quadratic sum of the random and systematic uncertainties. Total uncertainty includes a systematic component of ± 2% associated with laboratory beta-source calibration.

Table. S 11. D_e summary statistics, fading rates and final ages obtained for the Porto Maior luminescence dating samples. Data shown is for the pIR-IR_{225} signal measured on 160-grain aliquots containing K-feldspar grains.

Sample	Accepted/ measured	Over-dispersion (%)	CAM D_e (Gy) [a]	Total dose rate (Gy / ka)	Weighted skewness value [b]	Critical skewness 68% C.I. [b]	Critical skewness 95% C.I. [b]	Age (ka) [c]
PM16-4	54 / 1100	29 ± 5	60.8 ± 3.1	3.55 ± 0.18	-0.17	0.33	0.66	17 ± 1

[a] CAM = central age model of [54].

[b] Weighted skewness scores have been calculated on log-transformed D_e values using Eq. 7-8 [74]. Critical skewness scores have been calculated using Eq. 16 [55]. Critical skewness values are taken to be equivalent to the standard error of skewness score (68% C.I.) for multi-grain aliquot D_e datasets and twice the standard error of skewness score (95% C.I.) for single-grain D_e datasets, following the results of sensitivity analyses performed by [55] and [73].

[c] Mean ± total uncertainty (68% confidence interval), calculated as the quadratic sum of the random and systematic uncertainties. Total uncertainty includes a systematic component of ± 2% associated with laboratory beta-source calibration.

Table. S 12. D_e statistics and age obtained for sample PM16-4. Data shown is for single-grain OSL measurements made on 212-150 μm quartz grains.

Sample	Mineral	Grain fraction (µm)	Present-day water content (% dry weight)	Total dose rate (Gy/ka)	Age (ka)	Assumed long-term water content (% dry weight)	Total dose rate (Gy/ka)	Age (ka)
PM16-4	K-feldspar	90-125	9.7	4.34 ± 0.18	16 ± 1	15.0	4.14 ± 0.19	17 ± 1
PM16-4	Quartz	212-250	9.7	3.75 ± 0.18	16 ± 1	15.0	3.55 ± 0.18	17 ± 1
PM16-6	K-feldspar	90-125	13.5	4.06 ± 0.18	209 ± 12	24.9	3.69 ± 0.20	231 ± 15
PM16-5	K-feldspar	90-125	17.3	3.79 ± 0.18	263 ± 23	19.8	3.71 ± 0.19	268 ± 24
PM16-3	K-feldspar	90-125	18.8	4.10 ± 0.21	239 ± 26	28.5	3.79 ± 0.22	259 ± 29

Table. S 13. Ages obtained for the Porto Maior luminescence dating samples using the present-day ‘as measured’ water content values and the assumed long-term water content values. The latter have been used to derive the final age estimates and equate to 50% of the saturated water contents for samples PM16-3, PM16-5 and PM16-6, and a mean published value of 15 ± 5% for the uppermost aeolian sample PM16-4.

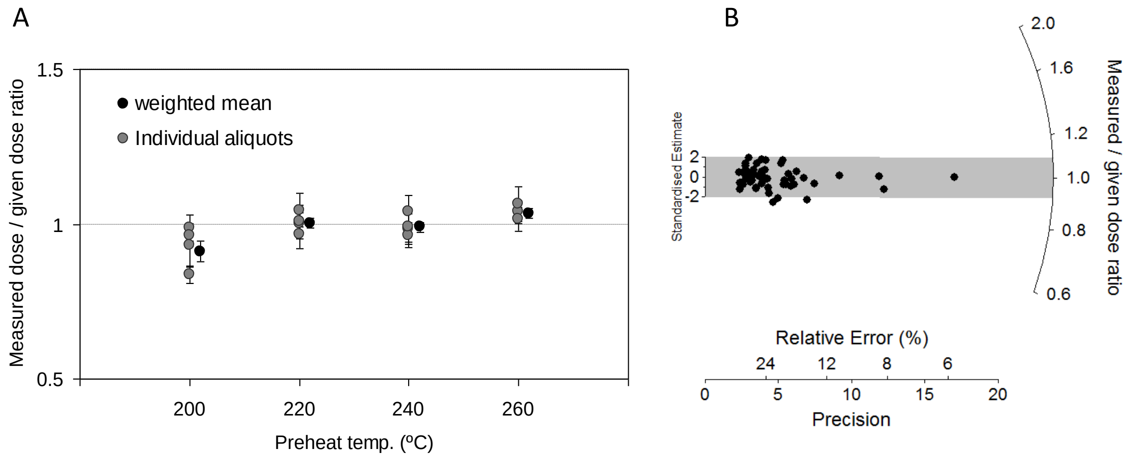

Fig. S 8. (A) Results for the OSL dose recovery test performed on sample PM16-4. Measurements were made on 1500-grain aliquots containing quartz grains and using a SAR protocol (see Table S7). The test-dose preheat used for this experiment was set to 160°C for 10 s for all measurements, while the regenerative dose preheat was varied between 200°C and 260°C (for 10 s) as shown. (B) Radial plot showing the single-grain OSL dose recovery test results obtained for sample PM16-4 using a regenerative-dose preheat of 220°C for 10 s and a test dose preheat of 160°C for 10 s. The grey bar is centred on a recovery dose ratio of 1 (the weighted mean measured to given dose ratio = 0.97 ± 0.02).

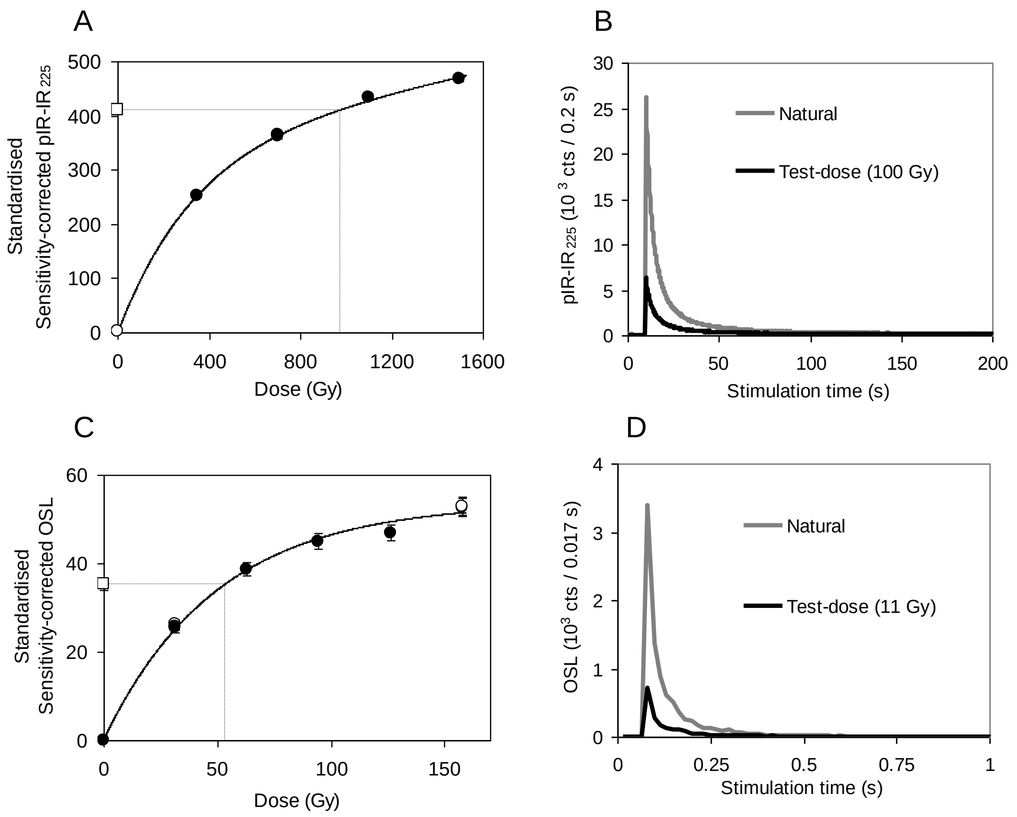

Fig. S 9. (A) Standardised sensitivity-corrected dose-response curve and (B) pIR-IR225 decay curves obtained for a 160-grain aliquot containing K-feldspar grains of sample PM16-5. (C) Standardised sensitivity-corrected dose-response curve and (D) corresponding OSL decay curves (first 1 s of the 2 s green laser stimulation) obtained for a quartz grain of sample PM16-4 that passed the SAR quality assurance criteria.

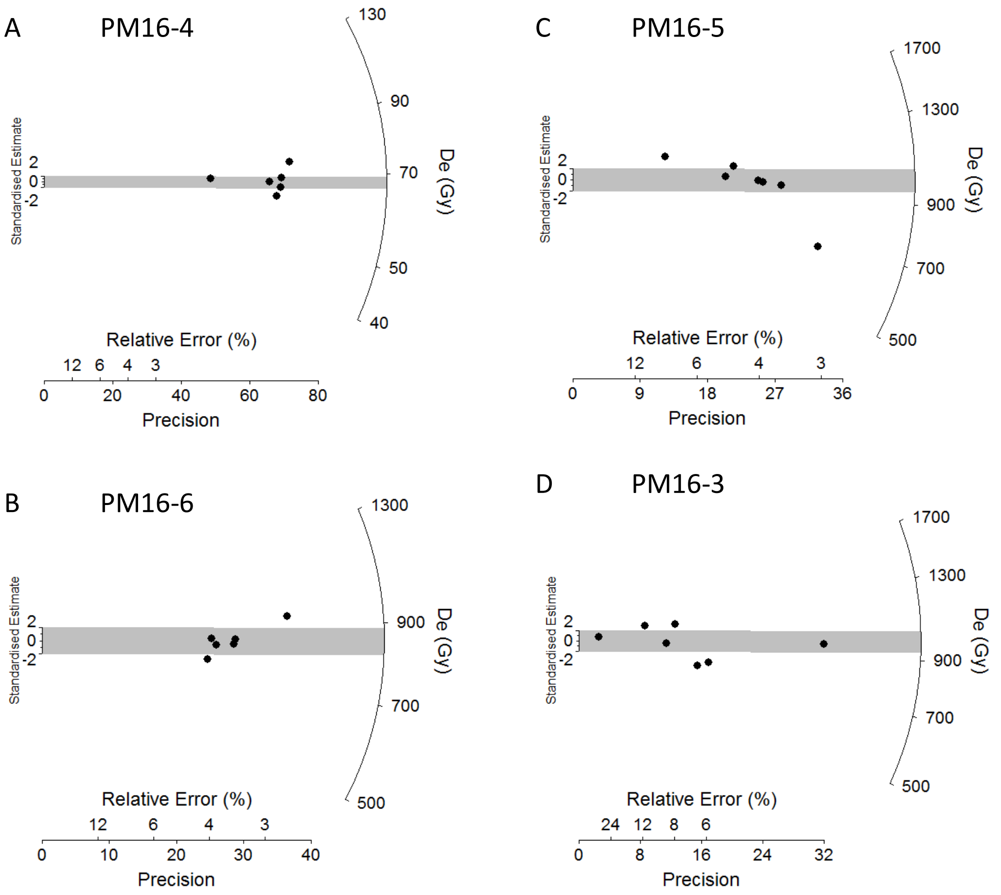

Fig. S 10. Radial plots showing 160-grain aliquot D_e values obtained for the Porto Maior K-feldspar samples using pIR-IR$_{225}$ protocol. The grey shaded band in each plot is centred on the weighted mean (CAM) D_e estimate.

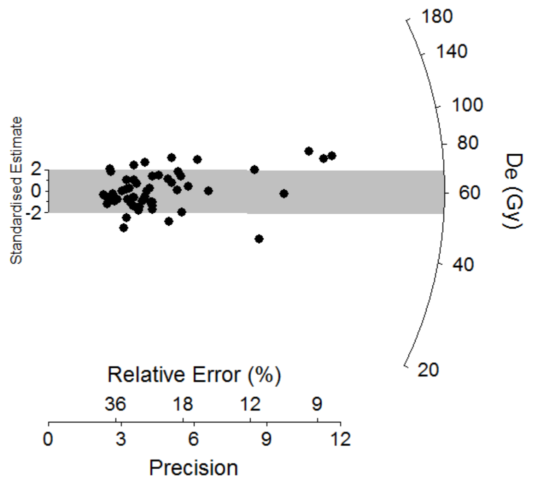

Fig. S 11. Single-grain OSL D_e distribution obtained for 212-250 µm quartz grains of sample PM16-4. The grey bar is centred on the CAM D_e (see Table S12 for details).

SI Site formation

Level PM4 has a thickness of ~80 cm and comprises fine sediments (22) that are indicative of a low energy sedimentation process (*Fsm* facies). The lithic material is located at the base of the level (Fig. 2, *Fig. S12-13*), resting on the top of the underlying level PM3. Taken together, 54.8 % of the lithic pieces from PM4 do not show signs of rolling or abrasion (R0), 25.2% show clear rolling (R1) and 20.0 % show intense rolling (R1-2 or R2) (*Fig. 14a*). Sub-dividing these statistics according to techno-typological composition reveals that the vast majority of LCTs do not have signs of fluvial abrasion (R0), while the rest of the tools show generalised and different intensity of rolling, of less abrasion (R1) to highest (R2) (*Table 1*).

This analysis allows us to identify two sets of lithic material with different taphonomic histories. The set displaying fluvial rolling (fluvial rolling assemblage; FRA) is composed essentially of flakes, waste, some cores and LCTs. The other set, which shows no fluvial abrasion (main assemblage; MA), consists primarily of LCTs (101 pieces made up of handaxes, cleavers and trihedral picks), LCT fragments (essentially handaxe or trihedral pick points) and some flakes, waste and cores. The MA corresponds to the main occupation level in PM4 and the FRA material would have been introduced by sedimentary processes that existed during the initial deposition of level PM4. Circular plots (*Fig. S14b*), dispersion parameters and statistical tests (Rayleigh and Kuiper) for unmodified pebbles and the MA material show uniform distributions without significant sedimentary organisations (*Table. S14*). FRA materials show a non-uniform distribution pattern, and are therefore indicative of hydraulic organization and disturbance.

Further evidence supporting the separation of the entire assemblage into sub-series comes from the size- and weight-range of the material (*Fig. S15-16*). The MA series is almost exclusively composed of large size pieces, while the FRA series shows a clear distribution toward smaller ranges. These differences relate to the degree of fluvial abrasion; the larger pieces are substantially less abraded than the smaller ones (*Fig. S15-16*).

Quantitative evaluation of the distribution patterns (*Fig. S17*), via the use of cluster analysis (k-means method[75,76]), revealed the identification of two statistically significant groups (*Fig. S17a*). These groups were analysed according to the type of series. We identify group 1 as being composed of pieces of smaller dimensions (flakes, cores, waste...), and group 2 primarily made of LCTs (*Fig. S17b-c*). The correlation between taphonomic and spatial pattern supports the separation in two different sub-series (MA, FRA series.

The sedimentary environment of level PM4, and the taphonomic conditions and the MA material fabric are compatible with an autochthonous position for the LCTs. This same interpretation applies to the large clasts, whose presence in this sedimentary context does not seem natural and would have to be related to the human activities developed in this level. The FRA material has likely been recycled from upstream or nearby deposits by sedimentary processes (potentially derived from units equivalent to PM3 at upstream localities), and are possibly not related to the main occupation of PM4.

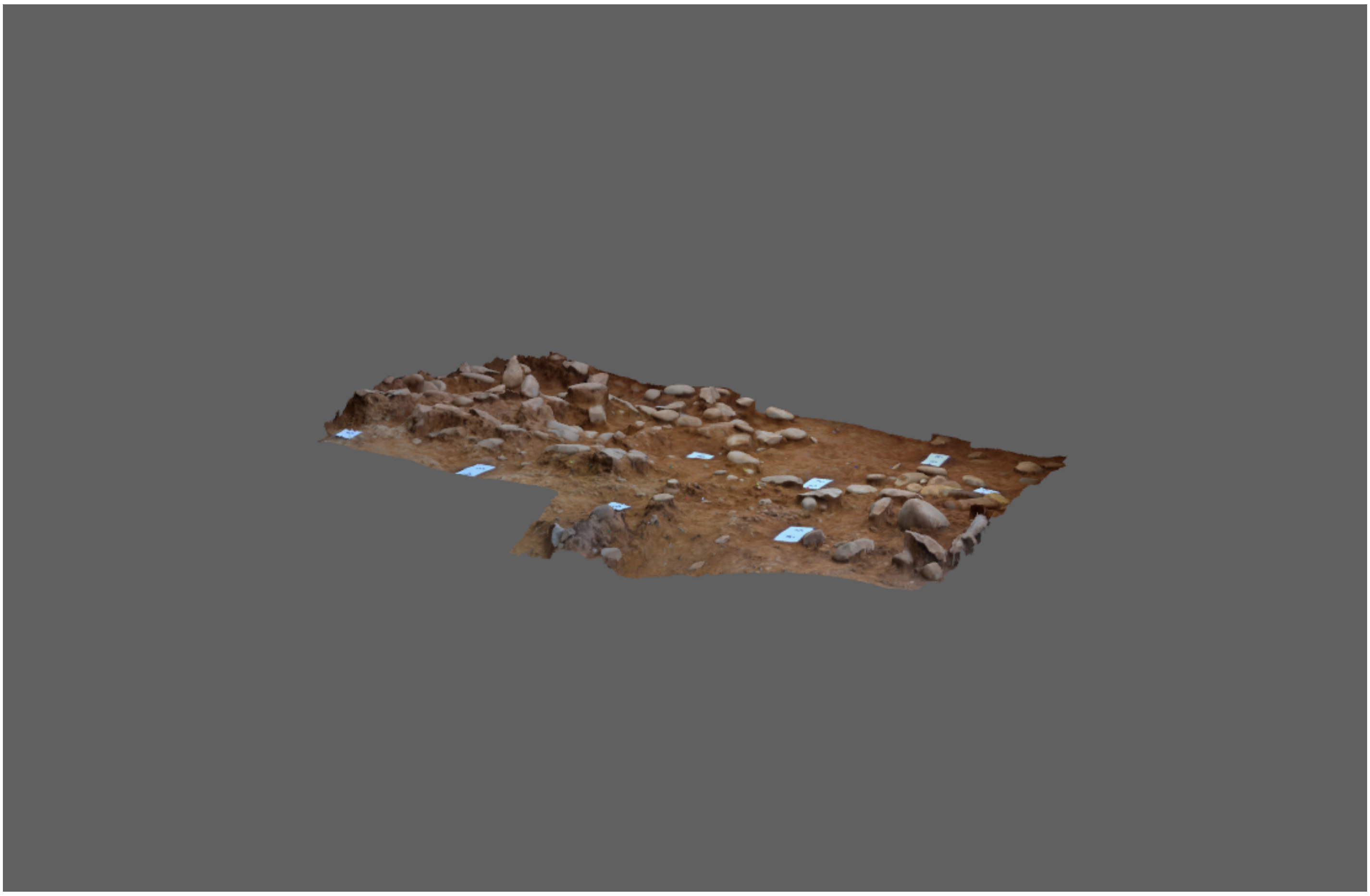

Fig. S 12. Interactive photogrammetric model of LCTs accumulation from level PM4 at Porto Maior, Spain. This model was created with software Agisoft PhotoScan Profesional 1.2.1.

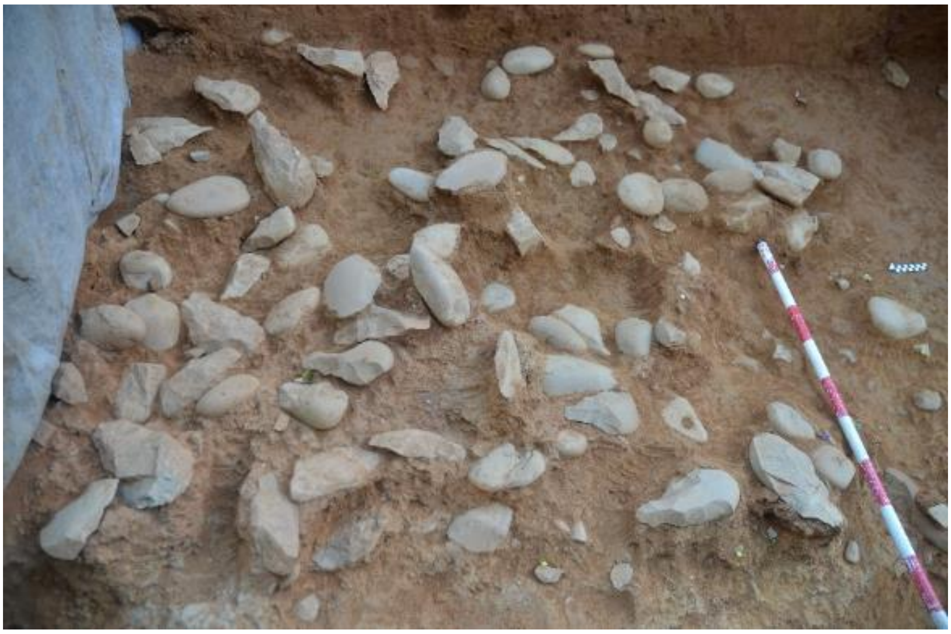

Fig. S 13. Detail of LCTs concentration showing the high number of handaxes, as well as some pebbles at the base of the layer, above level PM3. Photo by E. Méndez-Quintas

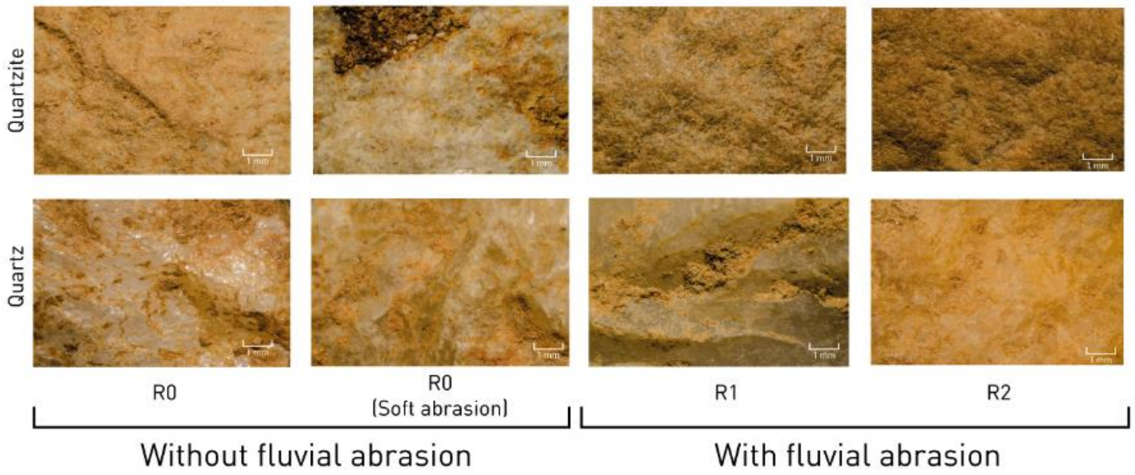

B

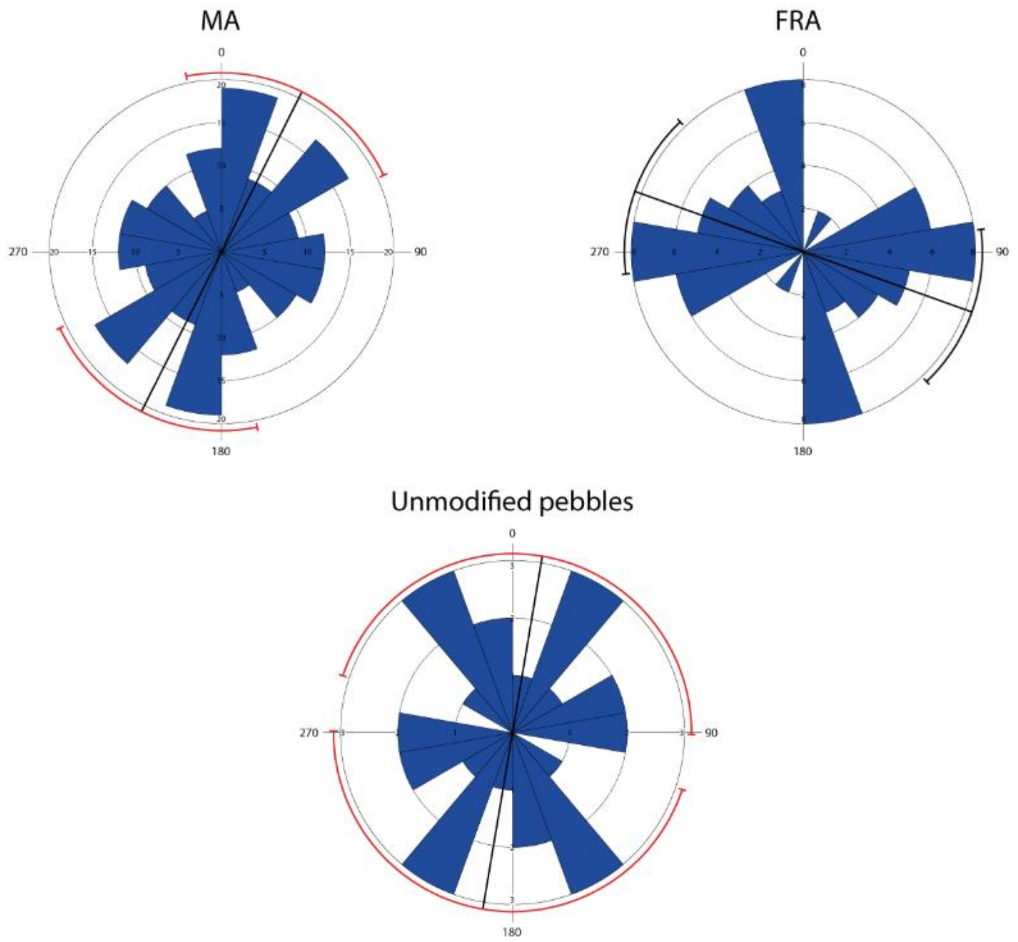

Fig. S 14. (A) Micro-photography of the different states of conservation (R0 does not have fluvial alteration, R1 have moderated alteration and R2 have severe alteration) for the industries from level PM4. (B) Circular histograms produced using the minimum bounding rectangle methods -MBR- [77] for MA, FRA and unmodified pebble assemblages (only items with elongation index > 1.6 and size > 5 cm). Note that the layer has a South slope. Drawing and photo by E. Méndez-Quintas.

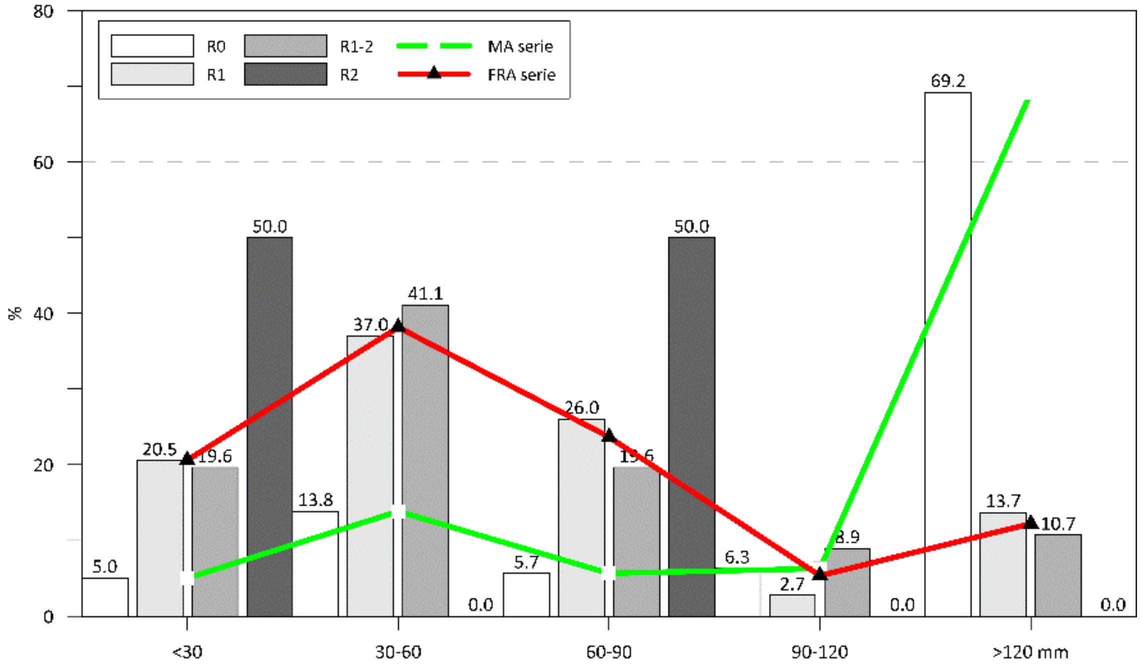

Fig. S 15. Size range and state of conservation for the industries from level PM4.

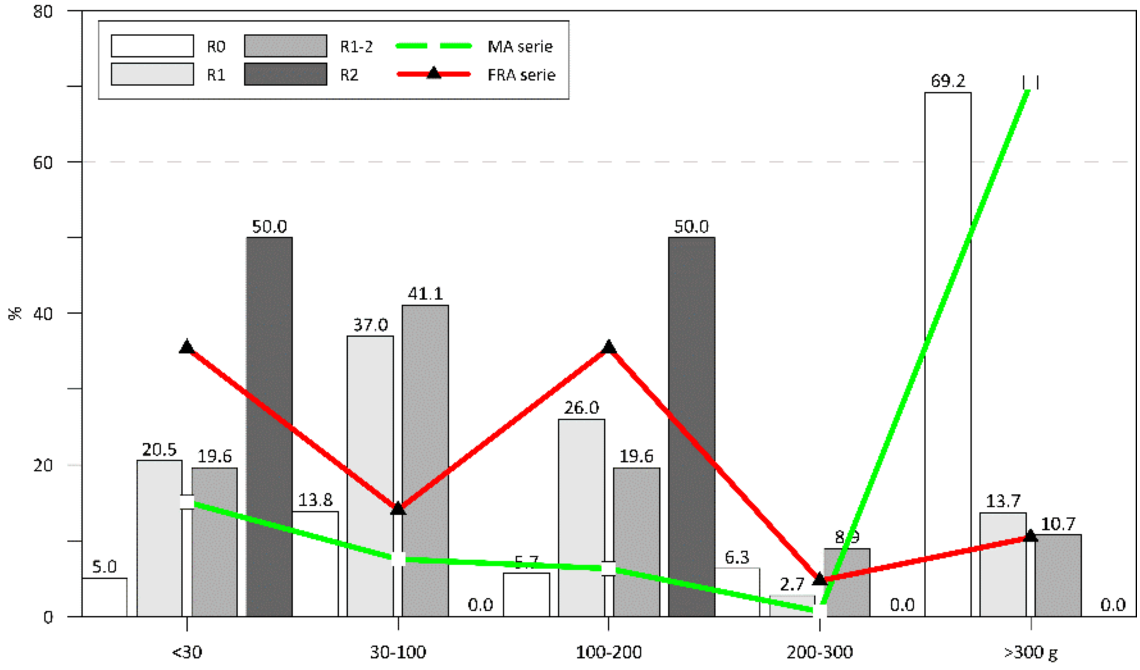

Fig. S 16. Weight range and state of conservation for the industries from level PM4.

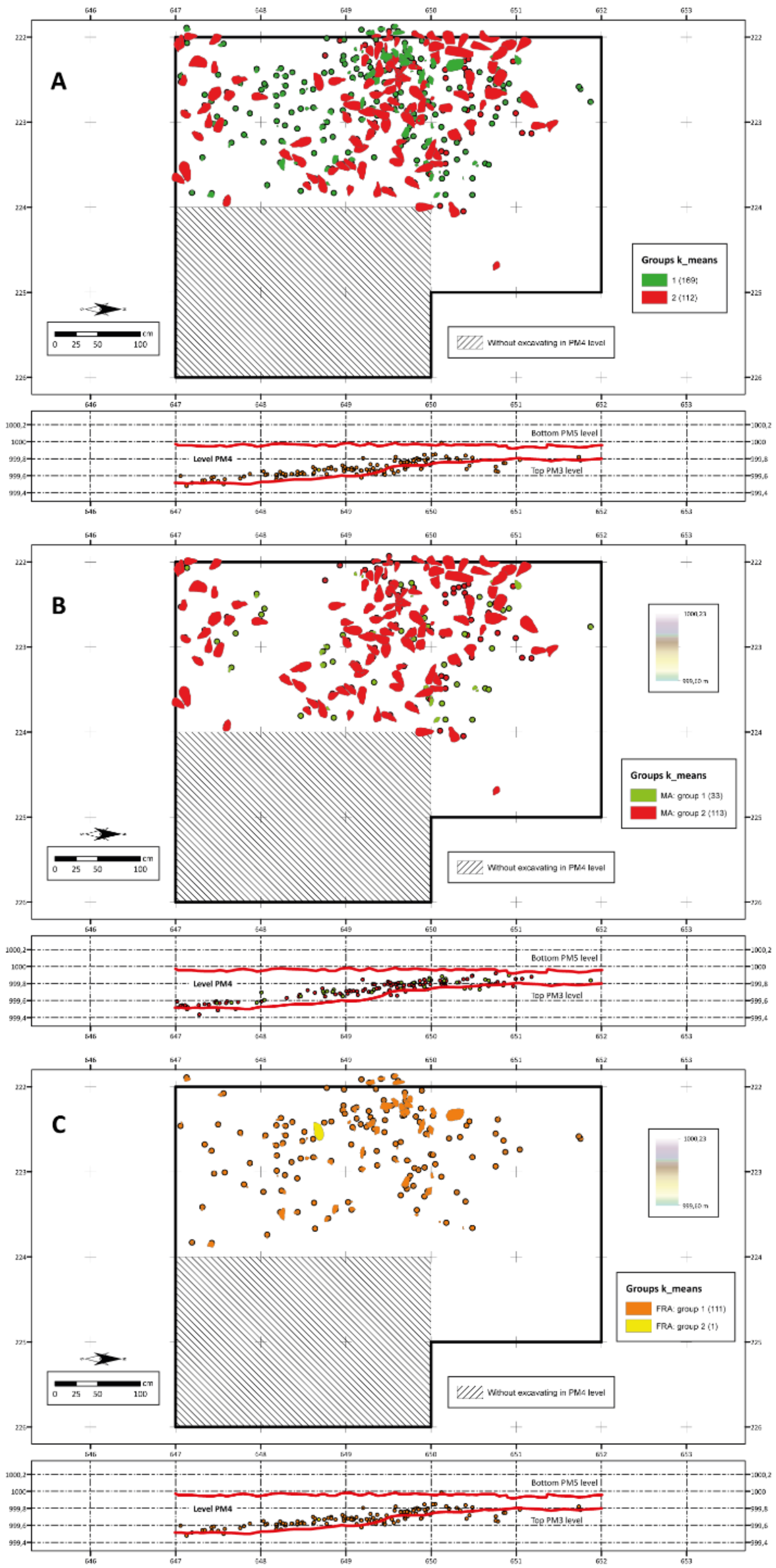
A
Groups k_means
1 (169)
2 (112)
Without excavating in PM4 level
Bottom PM5 level
Level PM4
Top PM3 level
B
Groups k_means
MA: group 1 (33)
MA: group 2 (113)
Without excavating in PM4 level
Bottom PM5 level
Level PM4
Top PM3 level
C
Groups k_means
FRA: group 1 (111)
FRA: group 2 (1)
Without excavating in PM4 level
Bottom PM5 level
Level PM4
Top PM3 level
0 25 50 100 cm
646 647 648 649 650 651 652 653
222 223 224 225 226
1000,2 1000 999,8 999,6 999,4

Fig. S 17. Grouping analysis (K-means) for the entire assemblage (A) and differentiated between the MA (B) and the FRA series (C). These maps were created with software ArcMap 10.4.1.

						Rayleigh´s Test		Kuiper's Test	
	n	Mean Vector (µ)	Mean Vector (r)	Concentration	Circular Variance	Z	p	V	p
Unmodified pebbles	15	9.554°	0.158	0	0.421	0.374	0.695	1.052	> 0.15
MA assemblage	105	26.642°	0.102	0.204	0.449	1.082	0.339	1.464	> 0.15
FRA assemblage	36	109.687°	0.243	0.501	0.379	2.125	0.119	1.759	< 0.05

Table. S 14. Dispersion parameters and statistical tests (Rayleigh and Kuiper) for the different assemblages of PM4 level. P values <0.05 indicate significant anisotropy (red) [78].

SI Technological features of the LCTs implements

The preferred blanks for the handaxes were pebbles (63.7 %), while the use of flakes as blanks was less frequent (25.0 %) (*Fig. S16-17)*. The presence of indeterminate blanks is low (8.7 %). The low percentages of flake blanks is related to the favourable size and shape of the pebbles, which eliminates the need for undertaking intermediate steps when obtaining a large flake. The analysed handaxes are almost always complete pieces (77.5 %) and, in limited cases, the extremities show signs of fracture (16.2 %) or reshaping (6.2 %). They show complete frontal and bilateral symmetry and their bases are mostly reserved (73.7%). Hard hammers were used exclusively in the initial shaping of the handaxes, and reshaping of the cutting edges can be observed in 68.7 % of the pieces. These were produced without the clear intervention of soft hammers. The number of scars that can be observed on the handaxes range between 5 and 39, with a mean of 17.2. The number of scars associated with reshaping is significantly less; these range between 3 and 25, with a mean of 7.9. This implies that, on average, 22.5 flakes were extracted in the complete conformation of these pieces (5 to 55 flakes). The mean volumetric dimension of the handaxes is 186.3 x 100.6 x 53.4 mm and the mean weight is 966.9 g (*Table. S16*). However, these values vary depending on the kind of handaxe, with the lanceolate having the largest dimensions (197.3 x 101.3 x 57.7 mm) (*Fig. S16*), followed by the transverse cutting edge (189.6 x 100.6 x 55.6 mm) and the amygdaloid (179.2 x 101.5 x 48.5 mm).

All cleavers were made on quartzite flakes. The configuration and reshaping of cleavers has been done exclusively with a hard hammer, with an average number of 9 scars per piece. The mean volumetric dimensions of the cleavers are 172.4 x 107 x 41.6 mm, and the mean weight is 891.3 g (*Table. S16*), which is generally smaller than the handaxes (186.3 x 100.6 x 53.4 mm and 966.9 g).

Trihedral picks usually are made of pebbles (50 %), even though flakes (25 %) and indeterminate supports (25 %) were also selected. On average, the initial volumetric reduction involved the extraction of 14.4 flakes and the reshaping of cutting edges required 14 extractions, which produced a total number of 19.2 scars per piece. Generally, trihedral picks are larger than handaxes and cleavers, with mean volumetric dimensions of 194.1 x 93.2 x 49.2 mm, and a mean weight of 899.9 g (*Table. S16*).

	Pieces		Weight (g.)	
	n	*%*	*n*	*%*
"Manuport"	0	0.0	0	0.0
Hammerstone/anvil	0	0.0	0	0.0
Flakes and flakes fragment	27	17.0	1270	1.1
Waste	8	5.0	1672	1.4
Cores	7	4.4	15669	13.3
Flake tools	5	3.1	763	0.6
LCTs and fragments	111	69.8	98037	83.0
Pebble tools	1	0.6	759	0.6
TOTAL	159		118170	

Table. S 15. Types of lithic implements in the MA assemblage, as well as the number of pieces and weight in each category.

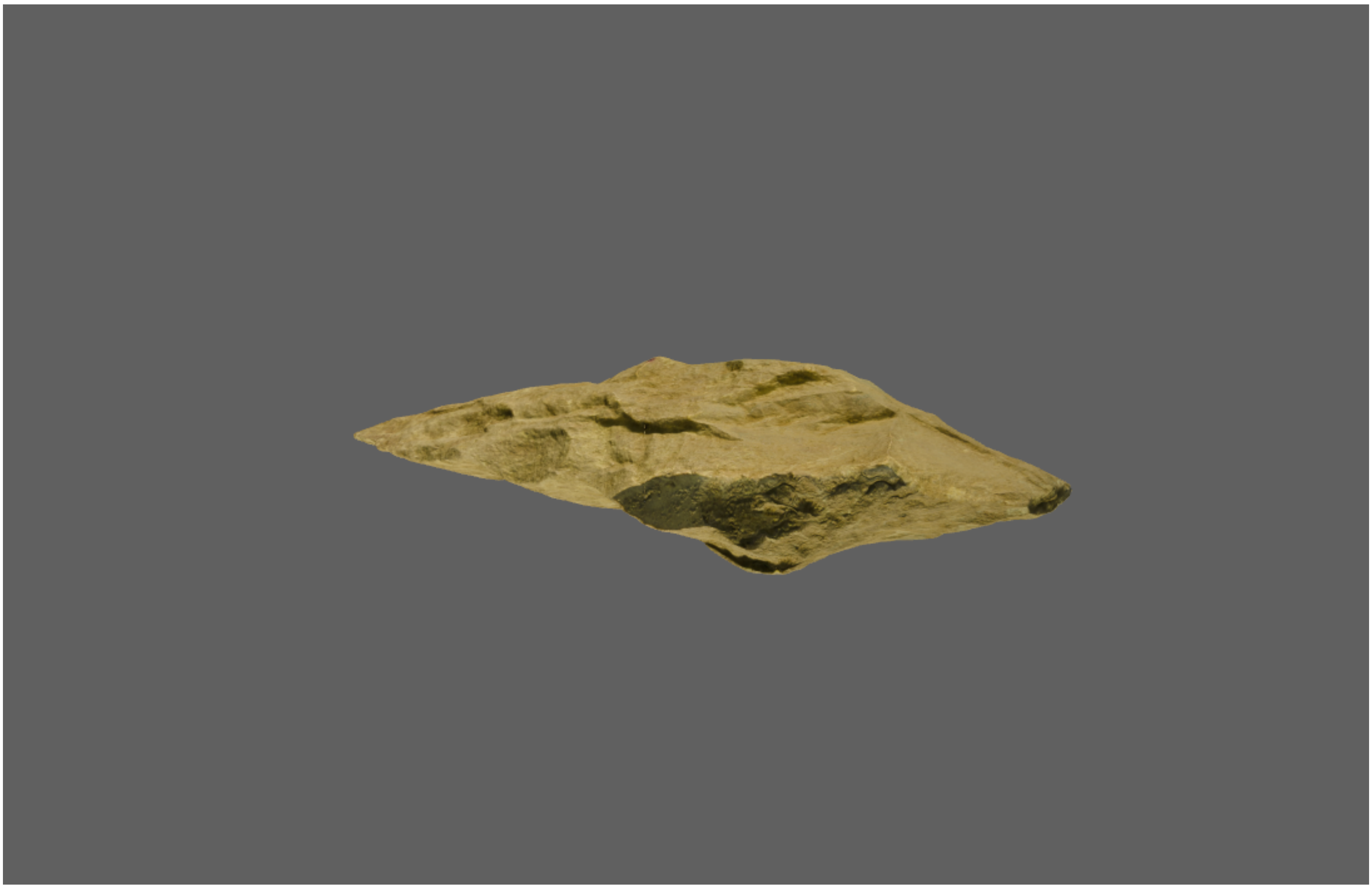

Fig. S 18. Interactive photogrammetric model of a lanceolate handaxe from level PM4. This model was created with software Agisoft PhotoScan Professional 1.2.1.

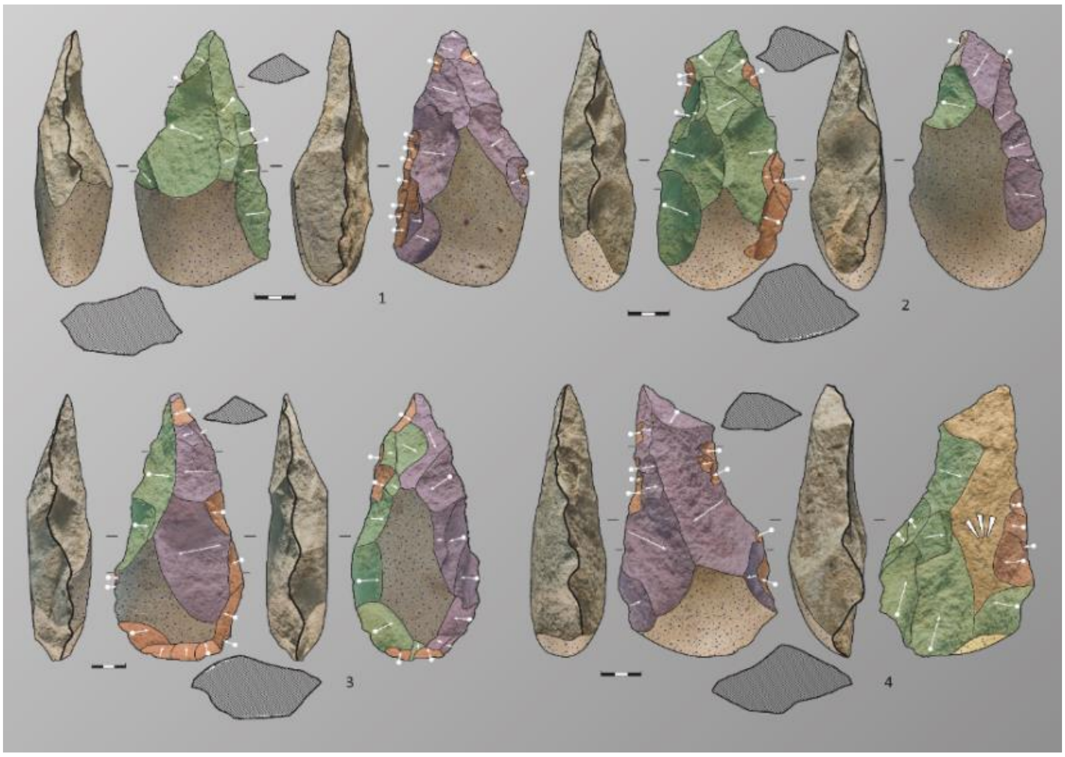

Fig. S 19. Examples of handaxes made on pebbles (1-3) or flakes (4). Drawing and photo by E. Méndez-Quintas.

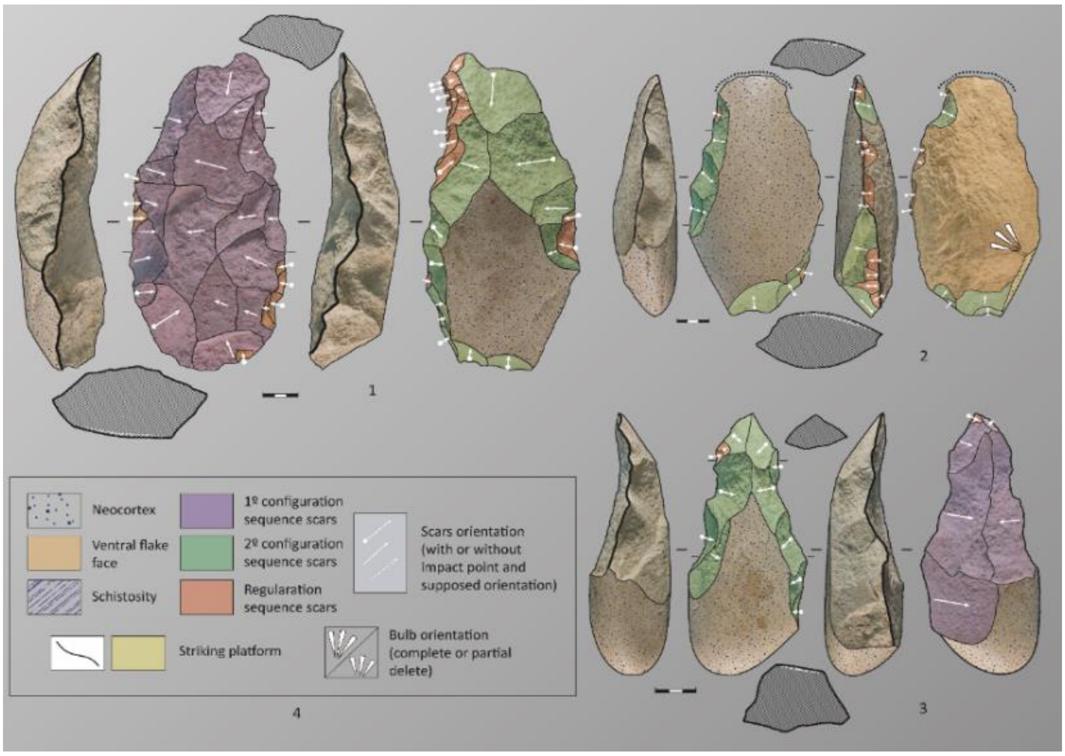

Fig. S 20. Handaxe on indeterminate blank (1), cleaver (2) and trihedral picks (3). (4) Legend of technical description for lithic industry shown. Drawing and photo by E. Méndez-Quintas.

Type	Length (mm)				Width (mm)				Thickness (mm)				Weight (g)			
	Min.	Max.	Mean	SD	Min.	Max.	Mean	SD	Min.	Max.	Mean	SD	Min.	Max.	Mean	SD
Handaxes (n=67)	82	254	186.4	32.5	63	126	100.6	12.8	30	74	53.4	10.7	141	2028	966.9	367.4
Cleavers (n=9)	119	254	172.4	44.6	84	137	107.0	20.7	32	57	41.7	9.2	394	1718	891.3	534.7
Trihedral picks (n=12)	128	274	194.1	39.7	69	115	93.3	15.5	27	67	49.3	12.9	352	1693	899.9	429.6

Table. S 16. Metric attributes for whole handaxes (without tip fractures), cleavers and trihedral picks from the MA collection at Porto Maior, Spain.

Site	Region	Chronology	Mean lenght LCTs	Range lenght LCTs	Mean weight LCTs	Range weight LCTs	References
Amanzi Springs	Africa	Middle Pleistocene?	115.9	93-250	782.0	250-3300	[79,80]
Cape Hanglink	Africa	Middle Pleistocene?	148.0	74-298	641.3	94-3080	[80]
DE-89B Olorgesaillie	Africa	900-700 ka	170.7	76-295	749.5	68-2327	[81,82]
Doornlaagate	Africa	Middle Pleistocene?	195.3	129-284	1142.8	360-2831	[80,83]
Eladsfoontein	Africa	~600 ka	123.9	62-212	371.0	60-1714	[80]
Isenya level VI	Africa	~700 ka	193.0	121-253	-	-	[84]
Isimila K-6	Africa	~300 ka	172.0	103-253	686.0	200-2900	[85,86]
Kalambo Falls B5 (level V)	Africa	~300 ka	171.0	61-360	648.0	88-3300	[87]
Kariandusi Upper	Africa	1000-780 ka	160.0	77-235	-	-	[88]
Kilombe GqJh 1 EH	Africa	>780 ka	152.0	-	-	-	[89,90]
Konso-Gardula 12-A1	Africa	~1400 ka	174.4	72-230	-	-	[91]
Konso-Gardula 8-A1	Africa	~1300 ka	168.0	88-280	-	-	[91]
Lepolosi (Peninj)	Africa	1500-1400 ka	105.9	70-186	817.4	293-1882	[92]
Montagu cave (level XI)	Africa	~600 ka	170.6	102-305	697.6	144-3030	[80,93]
Noolchalai (Peninj)	Africa	1500-1400 ka	155.3	97-250	834.1	104-2631	[94]
Olduvai: HK (Masek Beds)	Africa	600-400 ka	127.3	71-301	392.7	90-3101	[95]
Olduvai: WK (Bed IV)	Africa	>700 ka	129.7	54-190	490.1	92-1095	[95]
Olduvai: TK LF (Bed II)	Africa	~1400 ka	220.2	97-327	1453.9	219-3115	[96]
Olduvai: TK SF (Bed II)	Africa	~1400 ka	152.1	104-192	637.1	203-1045	[97]

Pniel 6	Africa	Middle Pleistocene?	113.7	85-207	360.6	91-922	[80]
Sidi Abderrahman: Cunnette	Africa	Middle Pleistocene?	114.3	61-199	306.4	60-997	[80]
Sidi Abderrahman: Grotte des Ours	Africa	Middle Pleistocene?	133.2	95-68	394.4	115-670	[80]
Sidi Abderrahman: STIC	Africa	Middle Pleistocene?	165.5	103-242	701.2	192-1504	[80]
Campsas (river Tarn)	Europe (France)	Middle Pleistocene	126.3	55-227	-	-	[98]
Gouzeacourt (level H)	Europe (France)	Late Middle Pleistocene	84.0	40-130	-	-	[99]
Boxgrove Q1/B	Europe (GB)	~400-300 ka	122.5	71-181	288.8	70-788	[80,100]
Corfe Mullen	Europe (GB)	Middle Pleistocene	121.2	56-183	344.4	41-1025	[80]
Cuxton	Europe (GB)	Middle Pleistocene	124.0	66-250	370.0	42-1233	[80,101,102]
Warren Hill	Europe (GB)	Middle Pleistocene	97.6	50-196	218.2	35-977	[80,103]
Ambrona LSC (AS1)	Iberian (Duero)	~450 ka					
Ambrona LSC (AS3)	Iberian (Duero)	~450 ka	129.4	87-169	435.0	139-533	[104]
Ambrona LSC (AS4)	Iberian (Duero)	~450 ka					
Burganes III	Iberian (Duero)	Middle Pleistocene?	142.6	118-184	723.3	340-1600	[105]
Calvarrasa I	Iberian (Duero)	Middle Pleistocene?	115.8	84-155	382.4	170-655	[105]
Galeria GII	Iberian (Duero)	~300 ka	117.8	-	-	-	[106]
Galeria GIII	Iberian (Duero)	~240 ka	108.2	-	-	-	[106]
Galisancho	Iberian (Duero)	Middle Pleistocene	127.2	72-226	579.7	85-1770	[107]
La Maya I +14 m	Iberian (Duero)	Middle Pleistocene	110.5	57-145	458.6	70-925	[105]
La Maya I +8 m	Iberian (Duero)	Middle Pleistocene	137.6	98-170	683.6	300-980	[105]
La Maya II	Iberian (Duero)	Middle Pleistocene	123.1	80-173	551.1	250-900	[105]
Portillo	Iberian (Duero)	Middle Pleistocene	127.0	59-164	580.3	50-1410	[105]
Torralba	Iberian (Duero)	~200 ka	111	58-178	-	-	[108]
Albalá	Iberian (Guadiana)	Middle Pleistocene	141.1	98-190	565.5	100-1010	[109]
El Martinete	Iberian (Guadiana)	Middle Pleistocene	144.2	100-186	635.8	160-1400	[109]
El Sotillo	Iberian (Guadiana)	Middle Pleistocene	89.1	56-167	-	-	[110]
Porzuna	Iberian (Guadiana)	Middle Pleistocene	133.7	52-236	470.6	35-2125	[111]
Casal do Azemel	Iberian (Lis)	Middle Pleistocene	122.2	60-195	364.0	35-1195	[112]
Arbo Fluvial	Iberian (Miño)	~150 ka	133.1	75-186	656.0	115-1330	[113]

Arbo OC1a	Iberian (Miño)	~150 ka	142.5	89-215	660.1	215-1330	[114]
Arbo OC2	Iberian (Miño)	~150 ka	142.7	78-203	595.2	281-1086	[114]
Chan do Cereixo	Iberian (Miño)	Middle Pleistocene	131.3	90-230	-	-	[115]
Gándaras de Budiño: fluvial level	Iberian (Miño)	Middle Pleistocene	110.5	72-170	432.6	110-1387	[116]
Gándaras de Budiño: lateral level	Iberian (Miño)	Middle Pleistocene	139.1	63-200	611.0	89-1486	[117]
**Porto Maior PM4 (MA excavation surface)**	**Iberian (Miño)**	**~240 ka**	**186.4**	**82-254**	**966.9**	**141-2028**	This paper
**Porto Maior PM4 (stratigraphic collection)**	**Iberian (Miño)**	**~240 ka**	**179.4**	**135-250**	**850.8**	**186-1774**	
Arenero Oxígeno	Iberian (Tajo)	Middle Pleistocene	121.9	41-220	-	-	[118]
El Sartalejo	Iberian (Tajo)	Middle Pleistocene	146.0	88-230	662.0	194-1696	[119,120]
Pinedo	Iberian (Tajo)	~300 ka	121.5	80-199	357.1	110-690	[121]
Puente Pino	Iberian (Tajo)	Middle Pleistocene	117.0	71-190	577.5	69-1725	[122]
San Isidro	Iberian (Tajo)	Middle Pleistocene	138.6	60-225	433.8	50-1345	[123]
Vale do Forno 3 (Milharõs)	Iberian (Tajo)	~150 ka	135.7	63-216	448.6	55-826	[124]
Gesher Benot Ya'aqov: Layer II-6 level 4	Near East	>750 ka	127.0	58-193	368.0	85-1100	[125]
K-30 ´Ubeidiya	Near East	~1300-1400ka	148.0	103-175	-	-	[126]

Table. S 17. Mean length and weight for handaxe collections from other Acheulean sites of Africa, Europe and the Near East.

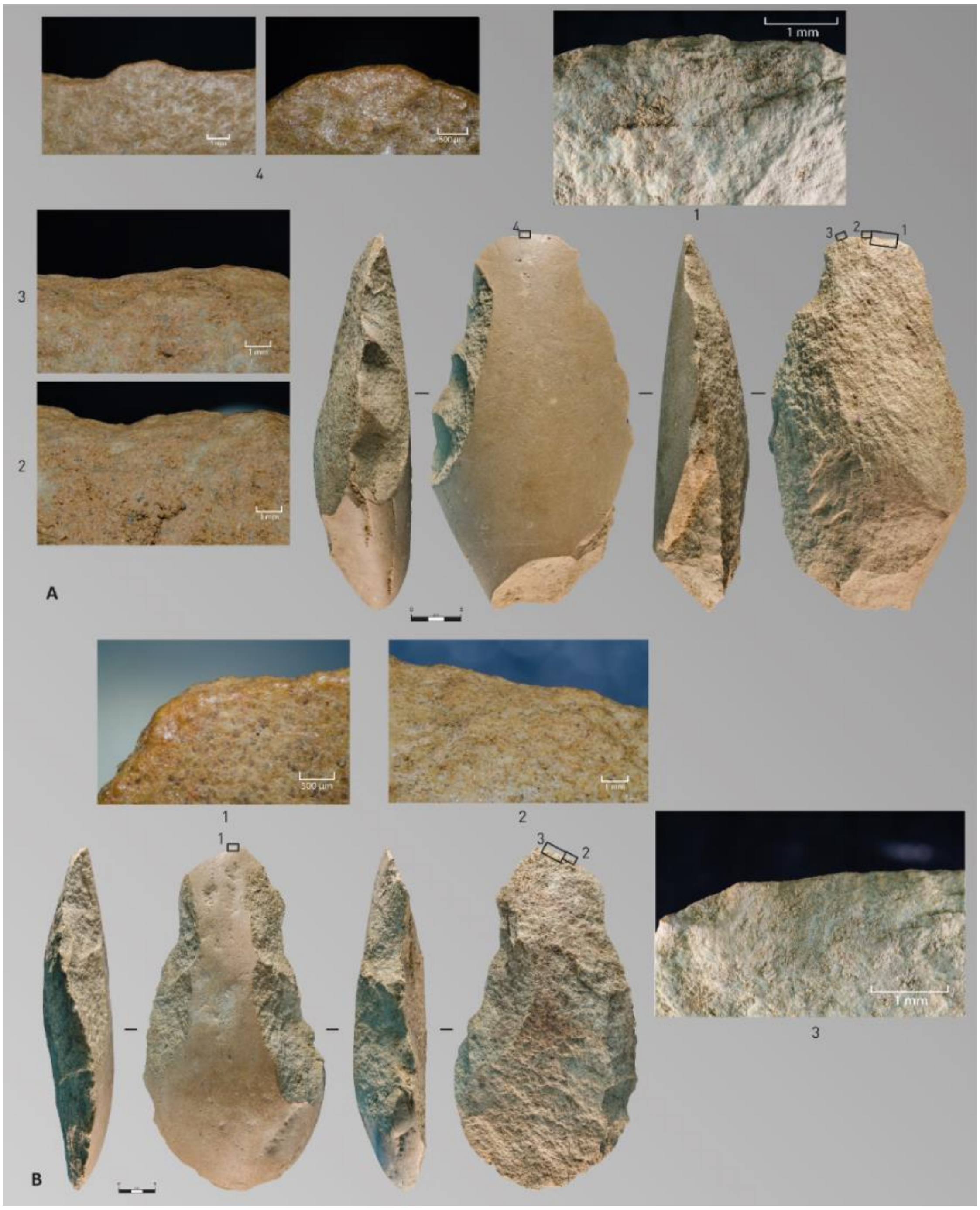

Fig. S 21. Examples of use-wear identified on the distal edges of two cleavers from level PM4: edge damage with micro flakes (A1, 3-4 and B2-3) and rounding (A4 and B1-2). Drawing and photo by E. Méndez-Quintas.

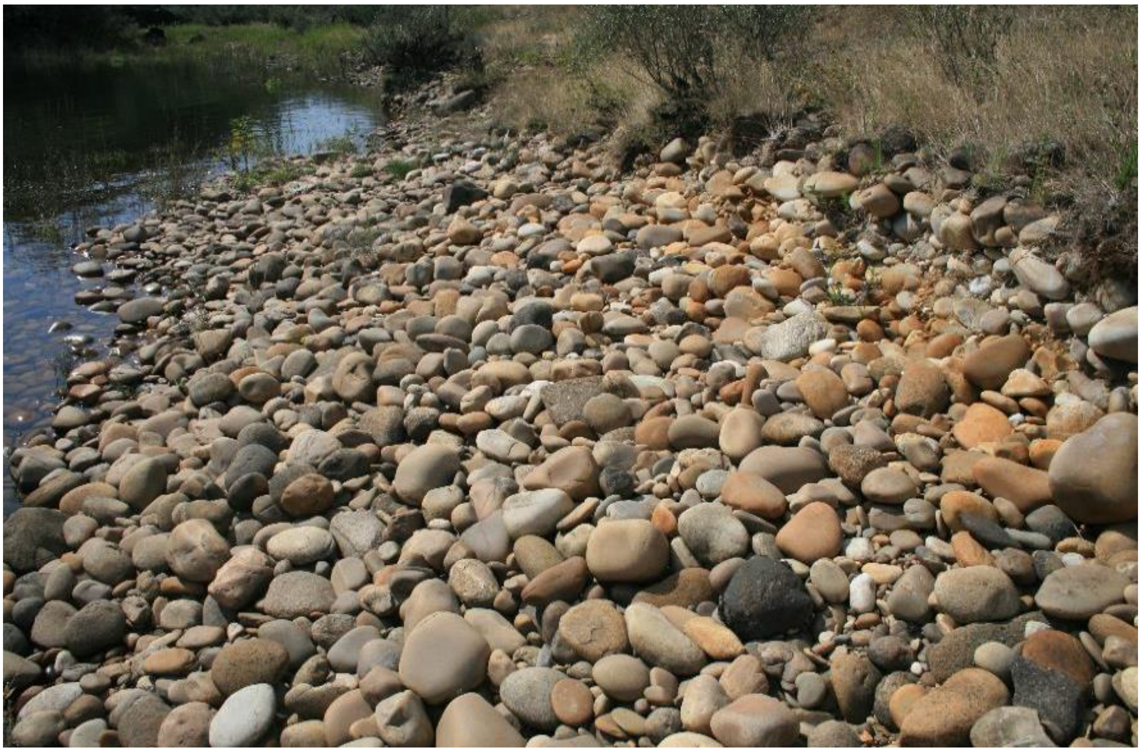

Fig. S 22. View of the present Miño floodplain in the vicinity of Porto Maior site showing the abundant presence of large quartzite pebbles and cobbles. Photo by E. Méndez-Quintas

SI Overview of extra-European sites with large LCT accumulations

Sites with large accumulations of LCTs are common in Africa and the Near East, but they were hitherto unknown in Europe (*Fig. S25; Table S17*). The most important West African LCT sites include TK in Olduvai, Noolchalai and Lepolosi in Peninj, Gadeb 8E, Garba I, Olorgesaillie, Kilombe, Kariandusi, Isenya and Kalambo Falls B5 [82,84,87,90,92,94,127-130]. In contrast, similar accumulations have not been identified so far in Northern Africa, although many Acheulean localities have been documented in this region [131,132]. In the Near East, similarly important LCT sites include level K 30 of ´Ubeidiya, Latamne and Layer II-6 Level 4 of Gesher Benot Ya'aqov [126,133,134].

Most of these sites are distinguished by large concentrations of LTCs, including 1503 LCTs in 151 m^2 at Isenya (Africa), and a density of 13,7 LCTs per m^2 at Benot Ya'aqov (Layer II 6 level 4) in the Neat East (Fig. 3; *Table S17*). Commonly, these implements are associated with variable percentages of flakes, cores and waste. However, it seems that the LCTs were configured elsewhere and were subsequently introduced to the site. Normally, the lithic assemblages are not found in direct stratigraphic relation with faunal

remains [82,135,136]. Where faunal remains do exist, they appear to be coincidental accumulations rather than the product of butchery practices.

These African and Near Eastern LCT sites comprise an extensive chronological range (1.3 Ma to 0.4 Ma), which spans almost the entire known duration of the African Acheulean. Generally, large LCT accumulations found in coarse fluvial facies, such as at Gadeb 8E, Garba I, Olorgesaillie DE-89, Kariandusi, Kalambo Falls B5 or ´Ubeidiya K 30, have been interpreted as potentially originating from natural sedimentary processes [137-140]. In other cases, such as TK in Olduvai, Noolchalai and Lepolosi in Peninj, or Layer II 6 Level 4 of Gesher Benot Ya'aqov, LCT accumulations are associated with low energy fluvial or lacustrine environments, and natural sedimentary processes have had little effect on accumulation of the archaeological record. At these sites, for which the lithic material has only been slightly affected by fluvial processes, the LCT accumulations are considered anthropic in origin [92,94,127].

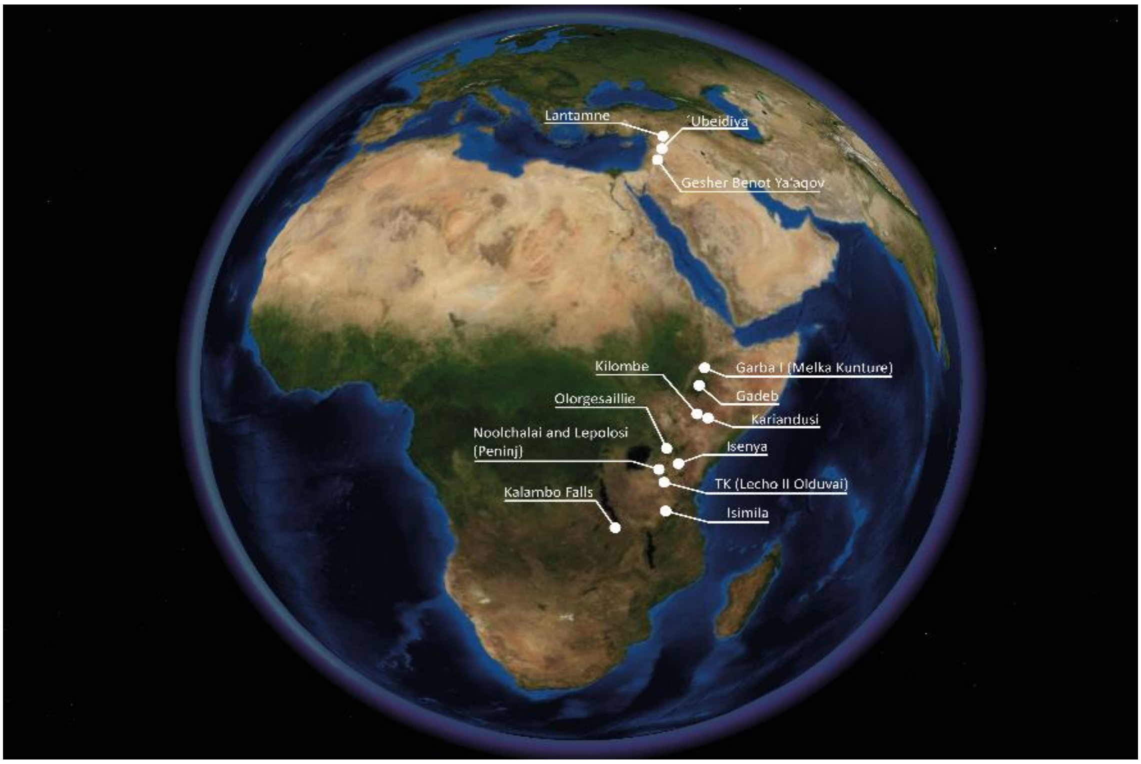

Fig. S 23. Location of the most important sites with large LCTs accumulations cited in the text. This map was created with software ArcGlobe 10.4.1.

Site	Region	Chronology	Geo. context	Alteration	Excavated area (m^2)	Artefacts	LCTs	% LCTs	% lithic m^2	% LCT m^2	Ref
DE-89B Olorgesaillie	Africa	900-700 ka	Fluvial channels	Yes	260	4751	523	11.01	18.27	**2.01**	[82]
Doornlaagte	Africa	Middle Pleistocene?	Fluvial lag	-	92,9	1920	118	6.15	20.67	**1.27**	[83]
Gadeb 8E	Africa	1500-1000 ka	Fluvial channels	Yes	95	20276	377	1.86	213.43	**3.97**	[128]
Grotte des Rhinocéros	Africa	~500 ka	Cave infill	No	70	3485	98	2.81	49.79	**1.40**	[141]
Isenya level V	Africa	~700 ka	Fluvial channels	Yes	151	1073	168	15.66	7.11	**1.11**	[84]
Isenya level VI	Africa	~700 ka	Fluvial channels	Yes	151	6394	1503	23.51	42.34	**9.95**	
Kalambo Falls B5 (level V)	Africa	~300 ka	Fluvial sands	Yes	20	539	94	17.44	26.95	**4.70**	[87,137]
Kariandusi Upper	Africa	1000-780 ka	Fluvial channels	Yes	27	715	171	23.92	26.48	**6.33**	[88]
Kilombe GqJh 1 EH	Africa	>780 ka	Fluvial channels	No?	25	1125	127	11.29	45.00	**5.08**	[90]
Lepolosi (Peninj)	Africa	1500-1400 ka	Deltaic floodplain	No	43	589	118	20.03	13.70	**2.74**	[92]
Melka Kunture: Garba I	Africa	600-400 ka	Fluvial channels	Yes	200	10000	-	-	50.00	**-**	[129]
Melka Kunture: Garba IVD	Africa	~1500 ka	Fluvial	Yes?	100	9821	23	0.23	98.21	**0.23**	[142]
Melka Kunture: Gombore II OAM	Africa	~780 ka	Fluvial	Yes?	35	1148	149	12.98	32.80	**4.26**	[143]
Montagu cave (level XI)	Africa	~600 ka	Cave infill	No	27	3322	118	3.55	123.04	**4.37**	[93]
Noolchalai (Peninj)	Africa	1500-1400 ka	Fluvial channels	Yes	38	583	58	9.95	15.34	**1.53**	[94]
Olduvai: WK (Bed IV)	Africa	>700 ka	Fluvial channels	Yes	150,5	10904	148	1.36	72.45	**0.98**	[144]
Olduvai: TK LF (Bed II)	Africa	~1400 ka	Loam channel facies	No	51,9	5805	85	1.46	111.85	**1.64**	[96]
Olduvai: TK SF (Bed II)	Africa	~1400 ka	Loam channel facies	No	45,3	1161	49	4.22	25.63	**1.08**	[97]
Cagny L´Epinette (level H)	Europe (France)	~300 ka	Fluvial terrace	No?	180	2249	24	1.07	12.49	**0.13**	[145]
Cagny-la-Garenne II (multi levels)	Europe (France)	~450 ka	Fluvial terrace	No?	60	9097	28	0.31	151.62	**0.47**	[146]
Ferme de l'Epinette	Europe (France)	~360 ka	Fluvial terrace	No?	2500	-	31	-	-	**0.01**	[147]
Gouzeacourt (level H)	Europe (France)	Late Middle Pleistocene	Karstic doline infill	No?	183	5729	282	4.92	31.31	**1.54**	[148]

Grande Vallée U5a	Europe (France)	~400 ka	Slope sediment	No?	15,5	11951	42	0.35	771.03	**2.71**	[149]
La Noira (level α)	Europe (France)	~700 ka	Fluvial	No?	100	341	58	17.01	3.41	**0.58**	[150,151]
Soucy 3P	Europe (France)	~350 ka	Fluvial terrace	No	700	6066	276	4.55	8.67	**0.39**	[152]
Boxgrove Q1/B (Middle unit)	Europe (GB)	~400-300 ka	Costal loam sediment	No	276	12813	321	2.51	46.42	**1.16**	[153]
Cuxton	Europe (GB)	Middle Pleistocene	Fluvial terrace	Yes	8	657	12	1.83	82.13	**1.50**	[101,102]
Guado San Nicola SU B	Europe (Italy)	~400-350 ka	Fluvial channels	No?	98	2018	85	4.21	20.59	**0.87**	[154]
Torre in Pietra (level m)	Europe (Italy)	~400 ka	Fluvial channels	Yes	200	536	34	6.34	2.68	**0.17**	[155]
Castel di Guido	Europe (Italy)	~300 ka	Lake facies (sands)	No?	1100	1131	99	8.75	1.03	**0.09**	[156,157]
Notarchirico A1-B	Europe (Italy)	~600 ka	Fluvial	Yes	10	41	9	21.95	4.10	**0.90**	[147,158]
Notarchirico B	Europe (Italy)	~600 ka	Fluvial	Yes	133	351	10	2.85	2.64	**0.08**	[147]
La Polledrara di Cecanibbio	Europe (Italy)	~300 ka	Fluvial channels	Yes	900	500	0	0.00	0.56	**0.00**	[159-161]
Ambrona LSC (AS1)	Iberian (Duero)	~450 ka	Aluvial fan	Yes	85	268	9	3.36	3.15	**0.11**	[104]
Ambrona LSC (AS3)	Iberian (Duero)	~450 ka	Lake shore	No	412	74	2	2.70	0.18	**0.00**	
Ambrona LSC (AS4)	Iberian (Duero)	~450 ka	Channel and overbank facies	No	580	353	1	0.28	0.61	**0.00**	
Galeria GII	Iberian (Duero)	~300 ka	Cave infill	Yes	124	1011	44	4.35	8.15	**0.35**	[162,163]
Galeria GIII	Iberian (Duero)	~240 ka	Cave infill	Yes	124	796	37	4.65	6.42	**0.30**	
La Maya I +14 m	Iberian (Duero)	Middle Pleistocene	Fluvial terrace	Yes	12	949	18	1.90	79.08	**1.50**	[105]

La Maya I +8 m	Iberian (Duero)	Middle Pleistocene	Fluvial terrace	Yes	8	506	9	1.78	63.25	**1.13**	[105]
Torralba	Iberian (Duero)	~200 ka	Fluvial terrace	Yes	2526	1529	216	14.13	0.61	**0.09**	[108]
Casal do Azemel	Iberian (Lis)	Middle Pleistocene	Colluvium	Yes	135	3654	545	14.92	27.07	**4.04**	[112]
Arbo OC1a	Iberian (Miño)	~150 ka	Fluvial sands	No	30	1292	62	4.80	43.07	**2.07**	[114]
Arbo OC2	Iberian (Miño)	~150 ka	Fluvial channels	Yes	8,7	1850	47	2.54	212.64	**5.40**	
Gándaras de Budiño: fluvial level	Iberian (Miño)	Middle Pleistocene	Fluvial terrace	No	25	1854	34	1.83	74.16	**1.36**	[116]
Gándaras de Budiño: lateral level	Iberian (Miño)	Middle Pleistocene	Alluvial fans	Yes	35	994	139	13.98	28.40	**3.97**	[117]
**Porto Maior PM4 (MA excavation surface)**	**Iberian (Miño)**	**~240 ka**	**Overbank facies**	**No**	**11,8**	**159**	**111**	**69.81**	**13.47**	**9.41**	This paper
Arganda I (Áridos I+II)	Iberian (Tajo)	~350 ka	Fluvial terrace	No	124	365	3	0.82	2.94	**0.02**	[164]
Arganda II (Valdocarros)	Iberian (Tajo)	~250 ka	Fluvial terrace	No	854	3009	69	2.29	3.52	**0.08**	[122]
Pinedo	Iberian (Tajo)	~300 ka	Fluvial terrace	Yes	25	5961	178	2.99	238.44	**7.12**	[121,165]
Puente Pino	Iberian (Tajo)	Middle Pleistocene	Fluvial terrace	No	7	579	23	3.97	82.71	**3.29**	[166]
Vale do Forno 3 (Milharõs)	Iberian (Tajo)	~150 ka	Alluvial fans	Yes	13	338	37	10.95	26.00	**2.85**	[124,167]
Gesher Benot Ya'aqov: Layer II-6 level 4	Near East	>750 ka	Lake shore	No	21	1845	227	12.30	87.86	**10.81**	[125]
K-30 ´Ubeidiya	Near East	~1300-1400ka	Fluvial channels	Yes	15	392	124	31.63	26.13	**8.27**	[126]
Latamne	Near East	Middle Pleistocene	Fluvial	Yes?	54	1831	99	5.41	33.91	**1.83**	[133]

Table. S 18. Principal characterises for the main Acheulean sites of Africa, Europe and the Near East.

SI References

1 Miall, A. D. *The Geology of Fluvial Deposits. Sedimentary Facies, Basin Analysis, and Petroleum Geology*. (Springer, 1996).

2 Schumm, S. A. *The fluvial system*. (Wiley Interscience, 1977).

3 Railsback, L. B., Gibbard, P. L., Head, M. J., Voarintsoa, N. R. G. & Toucanne, S. An optimized scheme of lettered marine isotope substages for the last 1.0 million years, and the climatostratigraphic nature of isotope stages and substages. *Quaternary Science Reviews* **111**, 94-106, doi:10.1016/j.quascirev.2015.01.012 (2015).

4 Duval, M. *et al.* Electron spin resonance dating of optically bleached quartz grains from the Middle Palaeolithic site of Cuesta de la Bajada (Spain) using the multiple centres approach. *Quaternary Geochronology* **37**, 82-96, doi:10.1016/j.quageo.2016.09.006 (2017).

5 Duval, M. & Guilarte, V. Assessing the influence of the cavity temperature on the ESR signal of the Aluminum center in quartz grains extracted from sediment. *Ancient TL* **30**, 11-16 (2012).

6 Toyoda, S., Voinchet, P., Falguères, C., Dolo, J. M. & Laurent, M. Bleaching of ESR signals by the sunlight: a laboratory experiment for establishing the ESR dating of sediments. *Applied Radiation and Isotopes* **52**, 1357-1362 (2000).

7 Toyoda, S. & Falgueres, C. The method to represent the ESR signal intensity of the aluminium hole center in quartz for the purpose of dating. *Advances in ESR Applications* **20**, 7-10 (2003).

8 Duval, M. & Guilarte, V. ESR dosimetry of optically bleached quartz grains extracted from Plio-Quaternary sediment: Evaluating some key aspects of the ESR signals associated to the Ti-centers. *Radiation Measurements* **78**, 28-41, doi:http://dx.doi.org/10.1016/j.radmeas.2014.10.002 (2015).

9 Forman, S. L., J., P. & Lepper, K. in *Quaternary Geochronology: methods and applications* (eds J. Sowers, J. Noller , & L. WR. Washington) 157-176 (American Geophysical Union, 2000).

10 Duval, M. Dose response curve of the ESR signal of the Aluminum center in quartz grains extracted from sediment. *Ancient TL* **30**, 1-9 (2012).

11 Duval, M. & Arnold, L. J. Field gamma dose-rate assessment in natural sedimentary contexts using LaBr3(Ce) and NaI(Tl) probes: A comparison between the “threshold” and “windows” techniques. *Applied Radiation and Isotopes* **74**, 36-45, doi:https://doi.org/10.1016/j.apradiso.2012.12.006 (2013).

12 Guérin, G., Mercier, N. & Adamiec, G. Dose-rate conversion factors: update. *Ancient TL* **29** (2011).

13 Vandenberghe, D., De Corte, F., Buylaert, J. P., Kučera, J. & Van den Haute, P. On the internal radioactivity in quartz. *Radiation Measurements* **43**, 771-775 (2008).

14 Yokoyama, Y., Falgueres, C. & Quaegebeur, J. P. ESR dating of quartz from quaternary sediments: First attempt. *Nuclear Tracks and Radiation Measurements* **10**, 921-928 (1985).

15 Brennan, B. J. Beta doses to spherical grains. *Radiation Measurements* **37**, 299-303, doi:https://doi.org/10.1016/S1350-4487(03)00011-8 (2003).

16 Brennan, B. J., Lyons, R. G. & Phillips, S. W. Attenuation of alpha particle track dose for spherical grains. *Nuclear Tracks and Radiation Measurements* **18**, 249-253 (1991).

17 Grün, R. A cautionary note: use of 'water content' and 'depth for cosmic ray dose rate' in AGE and DATA programs. *Ancient TL* **12**, 50-51 (1994).

18 Prescott, J. R. & Hutton, J. T. Cosmic ray contributions to dose rates for luminescence and ESR dating: Large depths and long-term time variations. *Radiation Measurements* **23**, 497-500 (1994).

19 Prescott, J. R. & Hutton, J. T. Cosmic ray and gamma ray dosimetry for TL and ESR. Nuclear Tracks. *Radiation Measurements* **14**, 223-227 (1988).
20 Aitken, M. J. *An Introduction to Optical Dating: The Dating of Quaternary Sediments by the Use of Photon-stimulated Luminescence*. (Oxford University Press, 1998).
21 Arnold, L. J., Duval, M., Falguères, C., Bahain, J. J. & Demuro, M. Portable gamma spectrometry with cerium-doped lanthanum bromide scintillators: Suitability assessments for luminescence and electron spin resonance dating applications. *Radiation Measurements* **47**, 6-18, doi:https://doi.org/10.1016/j.radmeas.2011.09.001 (2012).
22 Bøtter-Jensen, L. & Mejdahl, M. Assessment of beta dose-rate using a GM multicounter system. *Nuclear Tracks and Radiation Measurements* **14**, 187-191 (1988).
23 Huntley, D. J. & Baril, M. R. The K content of the K-feldspars being measured in optical dating or in thermoluminescence dating. *Ancient TL* **15**, 11-13 (1997).
24 Huntley, D. J. & Hancock, R. G. V. The Rb contents of the K-feldspar grains being measured in optical dating. *Ancient TL* **19**, 43-46 (2001).
25 Aitken, M. J. *Thermoluminescence Dating*. (Academic Press, 1985).
26 Readhead, M. L. Thermoluminescence dose rate and dating equations for the case of disequilibrium in the decay series. *Nuclear Tracks and Radiation Measurements* **13**, 197-207 (1987).
27 Berger, G. W. *et al.* Luminescence chronology of cave sediments at the Atapuerca paleoanthropological site, Spain. *Journal of Human Evolution* **55**, 300-311, doi:10.1016/j.jhevol.2008.02.012 (2008).
28 Arnold, L. J. *et al.* OSL dating of individual quartz 'supergrains' from the Ancient Middle Palaeolithic site of Cuesta de la Bajada, Spain. *Quaternary Geochronology* **36**, 78-101, doi:http://dx.doi.org/10.1016/j.quageo.2016.07.003 (2016).
29 Arnold, L. J., Demuro, M., Navazo, M., Benito-Calvo, A. & Pérez-González, A. OSL dating of Middle Palaeolithic Hotel California site, Sierra de Atapuerca, north-central Spain. *Boreas* **42**, 285-305 (2013).
30 Demuro, M., Arnold, L., Parés, J. M. & Sala, R. Extended-range luminescence chronologies suggest potentially complex bone accumulation histories at the Early-to-Middle Pleistocene palaeontological site of Huescar-1 (Guadix-Baza basin, Spain). *Quaternary International* **389**, 191-212, doi:10.1016/j.quaint.2014.08.035 (2015).
31 Derese, C., Vandenberghe, D., Paulissen, E. & Van den haute, P. Revisiting a type locality for Late Glacial aeolian sand deposition in NW Europe: Optical dating of the dune complex at Opgrimbie (NE Belgium). *Geomorphology* **109**, 27-35, doi:https://doi.org/10.1016/j.geomorph.2008.08.022 (2009).
32 Fitzsimmons, K. E., Hambach, U., Veres, D. & Iovita, R. The Campanian Ignimbrite Eruption: New Data on Volcanic Ash Dispersal and Its Potential Impact on Human Evolution. *PLOS ONE* **8**, e65839, doi:10.1371/journal.pone.0065839 (2013).
33 Wacha, L. & Frechen, M. The geochronology of the "Gorjanović loess section" in Vukovar, Croatia. *Quaternary International* **240**, 87-99, doi:https://doi.org/10.1016/j.quaint.2011.04.010 (2011).
34 Stevens, T., Marković, S. B., Zech, M., Hambach, U. & Sümegi, P. Dust deposition and climate in the Carpathian Basin over an independently dated last glacial–interglacial cycle. *Quaternary Science Reviews* **30**, 662-681, doi:https://doi.org/10.1016/j.quascirev.2010.12.011 (2011).
35 Schmidt, E. D., Frechen, M., Murray, A. S., Tsukamoto, S. & Bittmann, F. Luminescence chronology of the loess record from the Tönchesberg section: A comparison of using quartz and feldspar as dosimeter to extend the age range beyond the Eemian. *Quaternary International* **234**, 10-22, doi:https://doi.org/10.1016/j.quaint.2010.07.012 (2011).

36 Thiel, C., Horváth, E. & Frechen, M. Revisiting the loess/palaeosol sequence in Paks, Hungary: A post-IR IRSL based chronology for the 'Young Loess Series'. *Quaternary International* **319**, 88-98, doi:https://doi.org/10.1016/j.quaint.2013.05.045 (2014).
37 Vasiliniuc, Ş. *et al.* Testing the potential of elevated temperature post-IR IRSL signals for dating Romanian loess. *Quaternary Geochronology* **10**, 75-80, doi:https://doi.org/10.1016/j.quageo.2012.02.014 (2012).
38 Thomsen, K. J., Murray, A. S., Jain, M. & Bøtter-Jensen, L. Laboratory fading rates of various luminescence signals from feldspar-rich sediment extracts. *Radiation Measurements* **43**, 1474-1486 (2008).
39 Buylaert, J. P., Murray, A. S., Thomsen, K. J. & Jain, M. Testing the potential of an elevated temperature IRSL signal from K-feldspar. *Radiation Measurements* **44**, 560-565 (2009).
40 Thiel, C. *et al.* Luminescence dating of the Stratzing loess profile (Austria) – Testing the potential of an elevated temperature post-IR IRSL protocol. *Quarternary International* **234**, 23-31 (2011).
41 Arnold, L. J. *et al.* Evaluating the suitability of extended-range luminescence dating techniques over early and Middle Pleistocene timescales: Published datasets and case studies from Atapuerca, Spain. *Quaternary International* **389**, 167-190, doi:https://doi.org/10.1016/j.quaint.2014.08.010 (2015).
42 Wang, X. L. & Wintle, A. G. Investigating the contribution of recuperated TL to post-IR IRSL signals in a perthitic feldspar. *Radiation Measurements* **49**, 82-87 (2013).
43 Duller, G. A. Distinguishing quartz and feldspar in single grain luminescence measurements. *Radiation Measurements* **37**, 161-165 (2003).
44 Galbraith, R. F. A note on the variance of a background-corrected OSL count. *Ancient TL* **20**, 49-51 (2002).
45 Duller, G. A. Assessing the error on equivalent dose estimates derived from single aliquot regenerative dose measurements. *Ancient TL* **25**, 15-24 (2007).
46 Arnold, L. J. *et al.* Luminescence dating and palaeomagnetic age constraint on hominins from Sima de los Huesos, Atapuerca, Spain. *Journal of Human Evolution* **67**, 85-107, doi:10.1016/j.jhevol.2013.12.001 (2014).
47 Demuro, M. *et al.* New Luminescence Ages for the Galería Complex Archaeological Site: Resolving Chronological Uncertainties on the Acheulean Record of the Sierra de Atapuerca, Northern Spain. *PLOS ONE* **9**, e110169, doi:10.1371/journal.pone.0110169 (2014).
48 Auclair, M., Lamothe, M. & Huot, S. Measurement of anomalous fading for feldspar IRSL using SAR. *Radiation Measurements* **37**, 487-492 (2003).
49 Huntley, D. J. & Lamothe, M. Ubiquity of anomalous fading in K-feldspars and the measurement and correction for it in optical dating. *Canadian Journal of Earth Sciences* **38**, 1093-1106 (2001).
50 Buylaert, J.-P. *et al.* A robust feldspar luminescence dating method for Middle and Late Pleistocene sediments. *Boreas* **41**, 431-451 (2012).
51 Arsuaga, J. L. *et al.* Neandertal roots: Cranial and chronological evidence from Sima de los Huesos. *Science* **344**, 1358-1363, doi:10.1126/science.1253958 (2014).
52 Vasiliniuc, Ş. *et al.* Testing the potential of elevated temperature post-IR IRSL signals for dating Romanian loess. *Quaternary Geochronology* **10**, 75-80, doi:http://dx.doi.org/10.1016/j.quageo.2012.02.014 (2012).
53 Roberts, H. M. Testing Post-IR IRSL protocols for minimising fading in feldspars, using Alaskan loess with independent chronological control. *Radiation Measurements* **47**, 716-724, doi:http://dx.doi.org/10.1016/j.radmeas.2012.03.022 (2012).
54 Galbraith, R. F., Roberts, R. G., Laslett, G. M., Yoshida, H. & Olley, J. M. Optical dating of single and multiple grains of quartz from Jinmium Rock Shelter, Northern Australia: Part

1, experimental design and statistical models. *Archaeometry* **41**, 339-364, doi:10.1111/j.1475-4754.1999.tb00987.x (1999).

55 Bailey, R. N. & Arnold, L. J. Statistical modelling of single grain quartz De distributions and an assessment of procedures for estimating burial dose. *Quaternary Science Reviews* **25** (2006).

56 Arnold, L. J. & Roberts, R. G. Optically stimulated luminescence (OSL) dating of perennially frozen deposits in north-central Siberia: OSL characteristics of quartz grains and methodological considerations regarding their suitability for dating. *Boreas* **40**, 389-416 (2011).

57 Guérin, G. M., N. & Adamiec, G. Dose-rate conversion factors: update. *Ancient TL* **29**, 5-8 (2011).

58 Readhead, M. L. Absorbed dose fraction for 87Rb β particles. *Ancient TL* **20**, 25-28 (2002).

59 Mejdahl, V. Thermoluminescence dating: beta-dose attenuation in quartz grains. *Archaeometry* **21**, 61-72 (1979).

60 Mejdahl, V. Internal radioactivity in quartz and feldspar grains. *Ancient TL* **5**, 10-17 (1987).

61 Huntley, D. J. & Clague, J. J. Optical dating of tsunami-laid sands. *Quaternary Research* **46**, 171-140 (1996).

62 Huntley, D. J. & Lian, O. B. in *Holocene Climate and Environmental Change in the Palliser Triangle: A Geoscientific Context for Evaluating the Impacts of Climate Change on the Southern Canadian Prairies* (eds D.S. Lemmen & R. A. Vance) 211-222 (. Geological Survey of Canada, Ottawa, Bulletin 534, 1999).

63 Alappat, L. *et al.* Chronology of Cauvery Delta sediments from shallow subsurface cores using elevated-temperature post-IR IRSL dating of feldspar. *Geochronometria* **37**, 37-47 (2010).

64 Rees-Jones, J. Optical dating of young sediments using fine-grain quartz. *Ancient TL* **13**, 9-14 (1995).

65 Lang, A. & Wagner, G. A. Infrared stimulated luminescence dating of Holocene colluvial sediments using the 410 nm emission. *Quaternary Science Reviews* **16**, 393-396 (1997).

66 Banerjee, D., Murray, A. S., Bøtter-Jensen, L. & Lang, A. Equivalent dose estimation using a single aliquot of polymineral fine grains. *Radiation Measurements* **33**, 73-94 (2001).

67 Lang, A. *et al.* High-resolution chronologies for loess: comparing AMS 14C and optical dating results. *Quaternary Science Reviews* **22**, 953-959 (2003).

68 Feathers, J. K., Casson, M. A., Schmidt, A. H. & Chithamboet, M. L. Application of pulsed OSL to polymineral fine-grained samples. *Radiation Measurements* **47**, 201-209 (2012).

69 Bowler, J. M. *et al.* New ages for human occupation and climate change at Lake Mungo, Australia. *Nature* **421**, 837-840 (2003).

70 Jacobs, Z., Duller, G. A. T., Wintle, A. G. & Henshilwood, C. S. Extending the chronology of deposits at Blombos Cave, South Africa, back to 140 ka using optical dating of single and multiple grains of quartz. *Journal of Human Evolution* **51**, 255-273 (2006).

71 Pawley, S. M. *et al.* Age limits on Middle Pleistocene glacial sediments from OSL dating, north Norfolk, UK. *Quaternary Science Reviews* **27**, 1363-1377 (2008).

72 Rees-Jones, J. & Tite, M. S. Optical dating results for British archaeological sediments. *Archaeometry* **39**, 177-187 (1997).

73 Arnold, L. J., Bailey, R. M. & Tucker, G. E. Statistical treatment of fluvial dose distributions from southern Colorado arroyo deposits. *Quaternary Geochronology* **2**, 162-167, doi:http://dx.doi.org/10.1016/j.quageo.2006.05.003 (2007).

74 Arnold, L. J. & Roberts, R. G. Stochastic modelling of multi-grain equivalent dose (De) distributions: Implications for OSL dating of sediment mixtures. *Quaternary Geochronology* **4**, 204-230 (2009).

75 Sánchez-Romero, L., Benito-Calvo, A., Pérez-González, A. & Santonja, M. Assessment of Accumulation Processes at the Middle Pleistocene Site of Ambrona (Soria, Spain). Density and Orientation Patterns in Spatial Datasets Derived from Excavations Conducted from the 1960s to the Present. *PLOS ONE* **11**, e0167595, doi:10.1371/journal.pone.0167595 (2016).
76 de la Torre, I. & Wehr, K. Site formation processes of the early Acheulean assemblage at EF-HR (Olduvai Gorge, Tanzania). *J Hum Evol*, doi:10.1016/j.jhevol.2017.07.002 (2017).
77 de la Torre, I. & Benito-Calvo, A. Application of GIS methods to retrieve orientation patterns from imagery; a case study from Beds I and II, Olduvai Gorge (Tanzania). *Journal of Archaeological Science* **40**, 2446-2457, doi:10.1016/j.jas.2013.01.004 (2013).
78 Fisher, N. I. *Statistical Analysis of Circular Data*. (Cambrigde University Press, 1993).
79 Deacon, H. The Acheulian occupation at Amanzi Springs Uitenhage district, Cape Province. *Annals of the Cape Provincial Museums* **8**, 89-189 (1970).
80 Marshall, G., Dupplaw, D., Roe, D. A. & Gamble, C. Lower Palaeolithic technology, raw material and population ecology. doi:https://doi.org/10.5284/1000354 (2002).
81 Noll, M. P. *Component of Acheulean assemblage variability a Olorgesailie, Kenya*, University of Illinois, (2000).
82 Isaac, G. L. *Olorgesailie: archeological studies of a Middle Pleistocene lake basin in Kenya*. (University of Chicago Press, 1977).
83 Mason, R. J. in *Actas del V Congreso Panafricano de Prehistoria y de estudio del Cuaternario* (ed VV.AA) 187-188 (1966).
84 Roche, H., Brugal, J., Lefèvre, D., Ploux, S. & Texier, J. P. Isenya: état des recherches sur un nouveau site acheuléen d'Afrique orientale. *The African Archaeological Review* **6**, 27-55 (1988).
85 Hansen, C. L. & Keller, C. M. Environment and activity patterning at Isimila Korongo, Iringa District, Tanzania: A preliminary report. *American Anthropologist, New Series* **73**, 1201-1211 (1971).
86 Howell, F. C., Cole, G. H. & Kleindienst, M. R. in *4th PanAfrican Congress Proceedings* 44-81 (1962).
87 Clark, J. D. *Kalambo Falls prehistoric site Volume III*. (Cambridge University Press, 2001).
88 Gowlett, J. A. J. & Crompton, R. H. Kariandusi: Acheulean morphology and the question of allometry. *African Archaeological Review* **12**, 3-42 (1994).
89 Crompton, R. H. & Gowlett, J. Allometry an multidimensional forma in acheulean bifaces from Kilombe, Kenya. *Journal of Human Evolution* **25** (1993).
90 Gowlett, J. A. J. (B) Kilombe-an Acheulian site complex in Kenya. *Geological Society, London, Special Publications* **6**, 337-360, doi:10.1144/gsl.sp.1978.006.01.24 (1978).
91 Beyene, Y., Asfaw, B., Sano, K. & Suwa, G. in *The University of Tokyo Bulletin 48* 187 (Tokyo, 2015).
92 Diez-Martín, F. *et al.* Early Acheulean technology at Es2-Lepolosi (ancient MHS-Bayasi) in Peninj (Lake Natron, Tanzania). *Quaternary International* **322-323**, 209-236, doi:10.1016/j.quaint.2013.08.053 (2014).
93 Keller, C. Montagu Cave in prehistory. *University of California Archaeological Records* **28**, 1-150 (1973).
94 Diez-Martín, F. *et al.* Reassessment of the Early Acheulean at EN1-Noolchalai (Ancient RHS-Mugulud) in Peninj (Lake Natron, Tanzania). *Quaternary International* **322-323**, 237-263, doi:10.1016/j.quaint.2013.10.011 (2014).
95 Roe, D. in *Olduvai Gorge Volume 5. Excavations in Beds II, IV and Masek beds, 1968-1971* (eds M. D. Leakey & D Roe) 146-234 (Cambridge University Press, 1994).
96 Santonja , M. *et al.* in *The Emergence of the Acheulean in East Africa* (eds M. Mussi & R. Gallotti) (Vertebrate Paleobiology and Paleoantropology. Springer, 2016).

97 Rubio-Jara, S. *et al.* Site function and lithic technology in the Acheulean technocomplex: a case study from Thiongo Korongo (TK), Bed II, Olduvai Gorge, Tanzania. *Boreas*, doi:10.1111/bor.12275 (2017).
98 Tavoso, A. *Le Paléolitique Inférieur et moyen du Haut-Languedoc. Gisements des terrasses alluviales du Tarn, du Dadou, de l'Agout, du Sor et du Fresquel*. (1978).
99 McPherron, S. *A reduction model for variability in acheulian biface morphology*, University of Pennsylvania, (1994).
100 Roberts, M. B. & Parfitt, S. A. *Boxgrove : a Middle Pleistocene hominid site at Eartham Quarry, Boxgrove, West Sussex*. (English Heritage, 1999).
101 Cruse, R. J. Further investigation of the acheulian site of Cuxton. *Archaeology Cantiana* **104**, 39-81 (1987).
102 Tester, P. J. An acheulian site at Cuxton. *Archaeology Cantiana* **80**, 30-60 (1965).
103 Hardaker, T. The artefacts from the present land surface at the Palaeolithic site of Warren Hill, Suffolk, England. *Proceedings of the Geologists' Association* **123**, 692-713, doi:10.1016/j.pgeola.2012.05.009 (2012).
104 Santonja, M. *et al.* Ambrona revisited: The Acheulean lithic industry of the Lower Stratigraphic Complex. *Quaternary International* (2016).
105 Santonja, M. & Perez Gonzalez, A. *Las industrias paleolíticas de la Maya I en su ámbito regional*. (Excavaciones Arqueológicas en España 135, 1984).
106 García-Medrano, P., Olle, A., Mosquera, M., Cáceres, I. & Carbonell, E. The nature of technological changes: The Middle Pleistocene stone tool assemblages from Galería and Gran Dolina-subunit TD10.1 (Atapuerca, Spain). *Quaternary International* **368**, 92-111, doi:10.1016/j.quaint.2015.03.006 (2015).
107 Santonja, M. & Querol, M. A. Estudio de industrias del paleolítico Inferior procedentes de una terraza del Tormes (Galisancho, Salamanca). *Zephyrus* **26-27**, 97-109 (1976).
108 Sánchez-Cervera, B., Santonja Gómez, M., Pérez-González, A., Domínguez-Rodrigo, M. & Sánchez-Romero, L. La industria lítica del yacimiento achelense de Torralba (Soria, España). Colecciones marqués de Cerralbo y Howell. *Trabajos de Prehistoria* **72**, 41-63, doi:10.3989/tp.2015.12143 (2015).
109 Querol, M. A. & Santonja, M. in *Homenaje al prof. Martín Almagro Basch* Vol. 1 83-93 (Ministerio de Cultura, 1983).
110 Ciudad Serrano, A., García Serrano, R., Caballero Klink, A. & FRancia Villajos, A. Materiales paleolíticos de El Sotillo. *Estudios y Monografías. Museo de Ciudad Real* **8**, 1-72 (1983).
111 Cabrera Gómez, M. I. Los bifaces del yacimiento de Porzuna. *Oretum* **II**, 7-51 (1986).
112 Cunha Ribeiro, J.-P. *O acheulense no centro de Portugal: o Vale do Lis. Contribuicão para uma abordagem tecno-tipológica das suas indústrias líticas e problemática do seu contexto cronoestratigráfico* Tesis Toctoral thesis, Universidade de Lisboa, (1999).
113 Méndez Quintas, E., Santonja, M., Pérez-González, A., Ledo Bernárdez, M. & Serodio Domínguez, A. La industria lítica del yacimiento achelense de Arbo (Pontevedra). Variables del paleolítico antiguo de Galicia en el contexto peninsular. *Zephyrus* (In press).
114 Méndez Quintas, E. *et al.* An example of final Middle Pleistocene acheulean site in the river Miño basin (NW Iberian Peninsula). *PLOS ONE* (In press).
115 Vidal Encinas, J. M. La industria lítica de la estación achelense de Portavedra: Gondomar (Pontevedra). *El Museo de Pontevedra* **XXXV**, 55-85 (1981).
116 Méndez-Quintas, E. El yacimiento achelense de As Gándaras de Budiño. La industria en facies fluviales. *Complutum* **18**, 27-45 (2007).
117 Méndez-Quintas, E. La industria lítica de las facies coluviales del yacimiento achelense de As Gándaras de Budiño. El Locus V en las excavaciones de Vidal Encinas. *Zephyrus* **LXIII**, 41-61 (2008).

118 Rus, I. Arenero de oxígeno: bifaces, hendedores y triedros conservados en el Museo Arqueológico Nacional. *Trabajos de Prehistoria* **38**, 39-67 (1981).
119 Moloney, N. Lithic production and raw material explotation at the Middle Pleistocene site of El Sartalejo, Spain. *Papers from the Institute of Archaeology (PIA)* **3**, 11-22 (1992).
120 Santonja, M. *Yacimiento achelense de El Sartalejo (Valle de Alagón, Cáceres): estudio preliminar*. (1985).
121 Querol, M. A. & Santonja, M. El yacimiento achelense de Pinedo (Toledo). *Excavaciones Arqueológicas en España* **103** (1979).
122 Rubio-Jara, S., Panera, J., Rodríguez-de-Tembleque, J., Santonja, M. & Pérez-González, A. Large flake Acheulean in the middle of Tagus basin (Spain): Middle stretch of the river Tagus valley and lower stretches of the rivers Jarama and Manzanares valleys. *Quaternary International* **411**, 349-366, doi:10.1016/j.quaint.2015.12.023 (2016).
123 Santonja, M. Los bifaces del cerro de San Isidro (Madrid) conservados en el Museo Arqueologico Nacional. Intento de datación de una terraza de + 30 m. del Manzanares. *Revista de Archivos, Bibliotecas y Museos* **80**, 147-184 (1977).
124 Raposo, L., Carreira, J. R. & Salvador, M. in *I Reunião do Quaternário Ibérico, Lisboa 1985* Vol. II 41-60 (Grupo de Trabalho Português para o Estudo do Quaternário e Grupo Español de Trabajo del Quaternario, 1985).
125 Goren-Inbar, N., Sharon , G., Alperson-Afil, N. & Herzlinger, G. *The Acheulian Site of Gesher Benot Ya'aqov Vol IV: The Lithic Assemblages*. (Springer, 2016).
126 Bar-Yosef, O. & Goren-Inbar, N. in *Quedem* Vol. 34 266 (Institute of Archaeology. The Hebrew University of Jerusalem., 1993).
127 Santonja, M. *et al.* Technological strategies and the economy of raw materials in the TK (Thiongo Korongo) lower occupation, Bed II, Olduvai Gorge, Tanzania. *Quaternary International* **322–323**, 181-208, doi:10.1016/j.quaint.2013.10.069 (2014).
128 Clark, J. D. Transitions: Homo erectus and the Acheulian: the Ethiopian sites of Gadeb and the Middle Awash. *Journal of Human Evolution* **16**, 809-826 (1987).
129 Chavaillon, J. & Piperno, M. 712 (Istituto Italiano di Preistoria e Protostoria, 2004).
130 Shipton, C. Taphonomy and Behaviour at the Acheulean Site of Kariandusi, Kenya. *African Archaeological Review* **28**, 141-155, doi:10.1007/s10437-011-9089-1 (2011).
131 Raynal, J.-P. *et al.* Hominid Cave at Thomas Quarry I (Casablanca, Morocco): Recent findings and their context. *Quaternary International* **223-224**, 369-382 (2010).
132 Raynal, J. P., Sbihi Alaoui, F. Z., Geraads, D., Magoga, L. & Mohi, A. The earliest occupation of North-Africa: the Moroccan perspective. *Quarternary International* **75**, 65-75 (2001).
133 Clark, J. D. Acheulian Occupation Sites in the Middle East and Africa: A Study in Cultural Variability. *American Anthropologist* **68**, 202-229, doi:10.1525/aa.1966.68.2.02a001010 (1966).
134 Goren-Inbar, N. & Saragusti, I. An Acheulian Biface Assemblage from Gesher Benot Ya'aqov, Israel: indications of African affinities. *Journal of Field Archaeology* **23**, 15-30. (1996).
135 Díez-Martín, F. & Eren, M. in *Stone Tools and Fossil Bones: Debates in the Archaeology of Human Origins* (ed M. Dominguez- Rodrigo) 310-358 (Cambridge University Press, 2012).
136 Potts, R., Behrensmeyer, A. K. & Ditchfield, P. Paleolandscape variation and Early Pleistocene hominid activities: Members 1 and 7, Olorgesailie Formation, Kenya. *Journal of Human Evolution* **37**, 747-788, doi:10.1006/jhev.1999.0344 (1999).
137 Schick, K. Geoarchaeological analysis of an acheulean site at Kalambo Falls, Zambia. *Geoarchaeology* **7**, 1-26, doi:10.1002/gea.3340070102 (1992).
138 Petraglia, M. D. & Potts, R. Water Flow and the Formation of Early Pleistocene Artifact Sites in Olduvai Gorge, Tazania. *Journal of Anthropological Archaeology* **13**, 228-254 (1994).

139 Walter, M. J. & Trauth, M. H. A MATLAB based orientation analysis of Acheulean handaxe accumulations in Olorgesailie and Kariandusi, Kenya Rift. *Journal of Human Evolution* **64**, 569-581, doi:10.1016/j.jhevol.2013.02.011 (2013).
140 Shea, J. J. Artifact abrasion, fluvial processes, and "living floors" from the Early Paleolithic site of ʼUbeidiya (Jordan Valley, Israel). *Geoarchaeology* **14**, 191-207 (1999).
141 Raynal, J. P. & Mohib, A. 306 (Institut National des Sciences de l'Archéologie et du Patrimoine, Ministère de la Culture, 2017).
142 Gallotti, R. An older origin for the Acheulean at Melka Kunture (Upper Awash, Ethiopia): Techno-economic behaviours at Garba IVD. *Journal of Human Evolution* **65**, 594-620, doi:http://dx.doi.org/10.1016/j.jhevol.2013.07.001 (2013).
143 Gallotti, R. *et al.* The Early Middle Pleistocene Site of Gombore II (Melka Kunture, Upper Awash, Ethiopia) and the Issue of Acheulean Bifacial Shaping Strategies. *African Archaeological Review* **27**, 291-322, doi:10.1007/s10437-010-9083-z (2010).
144 Leakey, M. D., Roe, D. A. & Callow, P. *Olduvai Gorge. Vol.5, Excavations in Beds III, IV and the Masek Beds, 1968-1971*. (Cambridge University Press, 1994).
145 Tuffreau, A., Lamotte, A. & Marcy, J.-L. Land-Use and Site Function in Acheulean Complexes of the Somme Valley. *World Archaeology* **29**, 225-241 (1997).
146 Tuffreau, A. in *Volumen 6 de Publications du CERP* 239 (2001).
147 Nicoud, E. in *Documents Prehistoriques 32* 309 (Bibliothèque des Écoles Française d´Athènes et de Rome, 2013).
148 Tuffreau, A., Lamotte, A. & Goval, E. Les industries acheuléennes de la France septentrionale. *L'Anthropologie* **112**, 104-139, doi:10.1016/j.anthro.2008.01.003 (2008).
149 Hérisson, D. *et al.* Between the northern and southern regions of Western Europe: The Acheulean site of La Grande Vallée (Colombiers, Vienne, France). *Quaternary International* **411**, 108-131, doi:10.1016/j.quaint.2015.12.100 (2016).
150 Despriee, J. *et al.* The Middle Pleistocene site of La Noira at Brinay (Cher): morphosedimentary context, geochronology, archaeological data. *Quaternaire* **28**, 31-48 (2017).
151 Despriee, J. *et al.* The Acheulean site of la Noira (Centre region, France): Characterization of materials and alterations, choice of lacustrine millstone and evidence of anthropogenic behaviour. *Quaternary International* **411**, 144-159, doi:10.1016/j.quaint.2015.12.101 (2016).
152 Lhomme, V. Tools, space and behaviour in the Lower Palaeolithic: discoveries at Soucy in the Paris basin. *Antiquity* **81**, 536 (2007).
153 Pope, M. *The significance of biface-rich assemblages: An examination of behavioural controls on lithic assemblage formation in the Lower Palaeolithic.*, University of Southampton, (2002).
154 Peretto, C. *et al.* The Middle Pleistocene site of Guado San Nicola (Monteroduni, Central Italy) on the Lower/Middle Palaeolithic transition. *Quaternary International* **411**, 301-315, doi:http://dx.doi.org/10.1016/j.quaint.2015.11.056 (2016).
155 Villa, P. *et al.* The Acheulian and Early Middle Paleolithic in Latium (Italy): Stability and Innovation. *PLoS ONE* **11**, e0160516, doi:10.1371/journal.pone.0160516 (2016).
156 Boschian, G. & Saccà, D. Ambiguities in human and elephant interactions? Stories of bones, sand and water from Castel di Guido (Italy). *Quaternary International* **214**, 3-16, doi:10.1016/j.quaint.2009.10.016 (2010).
157 Mariani-Costantini, R. Taphonomy of the fossil hominid bones from the Acheulean site of Castel di Guido near Rome, Italy. *Journal of Human Evolution* **41**, 211-225, doi:10.1006/jhev.2001.0492 (2001).
158 Piperno, M. & Tagliacozzo, A. The elephant butchery area at the Middle Pleistocene site of Notarchirico (Venosa, Basilicata, Italy). *La Terra Degli Elefanti. Consiglio Nazionale delle Ricerche, Rome*, 230-236 (2001).

159 Pereira, A. *et al.* 40 Ar/ 39 Ar and ESR/U-series data for the La Polledrara di Cecanibbio archaeological site (Lazio, Italy). *Journal of Archaeological Science: Reports* **15**, 20-29, doi:10.1016/j.jasrep.2017.05.025 (2017).

160 Santucci, E. *et al.* Palaeoloxodon exploitation at the Middle Pleistocene site of La Polledrara di Cecanibbio (Rome, Italy). *Quaternary International* **406**, 169-182, doi:http://dx.doi.org/10.1016/j.quaint.2015.08.042 (2016).

161 Anzidei, A. P. *et al.* Ongoing research at the late Middle Pleistocene site of La Polledrara di Cecanibbio (central Italy), with emphasis on human-elephant relationships. *Quaternary International* **255**, 171-187, doi:10.1016/j.quaint.2011.06.005 (2012).

162 García-Medrano, P., Cáceres, I., Olle, A. & Carbonell, E. The occupational pattern of the Galería site (Atapuerca, Spain): A technological perspective. *Quaternary International*, doi:10.1016/j.quaint.2015.11.013 (2016).

163 Díez Fernández-Lomana, J. C., Rosas, A. & Carbonell, E. 390 (Junta de Castilla y León, Consejería de Educación y Cultura, 1999).

164 Santonja, M., López Martínez, L. & Pérez-González, A. in *Arqueología y Paleoecología Arqueología y Paleoecología* 365 (Diputación Provincial de Madrid, 1980).

165 López-Recio, M. *et al.* Geocronología de los yacimientos achelenses de Pinedo y Cien Fanegas (Valle del Tajo) e implicaciones en la evolución fluvial en el entorno de Toledo (España). *Estudios Geológicos* **71**, e029, doi:10.3989/egeol.41816.340 (2015).

166 Rodríguez de Tembleque Moreno, J. M. Yacimiento de Puente Pino: nuevas perspectivas en el estudio del Paleolítico inferior de la Península Ibérica. *Zona Arqueológica* **4**, 440-451 (2004).

167 Cunha, P. P. *et al.* New data on the chronology of the Vale do Forno sedimentary sequence (Lower Tejo River terrace staircase) and its relevance as a fluvial archive of the Middle Pleistocene in western Iberia. *Quaternary Science Reviews* **166**, 204-226, doi:10.1016/j.quascirev.2016.11.001 (2017).
